# Brain Abnormalities in Children Exposed Prenatally to the Pesticide Chlorpyrifos

**DOI:** 10.1001/jamaneurol.2025.2818

**Published:** 2025-08-18

**Authors:** Bradley S. Peterson, Sahar Delavari, Ravi Bansal, Siddhant Sawardekar, Chaitanya Gupte, Howard Andrews, Lori A. Hoepner, Wanda Garcia, Frederica Perera, Virginia Rauh

**Affiliations:** 1 Institute for the Developing Mind, Children’s Hospital Los Angeles, Los Angeles, California; 2 Department of Psychiatry, Keck School of Medicine at the University of Southern California, Los Angeles; 3 Department of Psychiatry, Columbia Presbyterian Medical Center & New York State Psychiatric Institute, New York; 4 Department of Environmental and Occupational Health Sciences, SUNY Downstate School of Public Health, Brooklyn, New York; 5 Heilbrunn Department of Population and Family Health, Mailman School of Public Health, Columbia University, New York, New York; 6 Columbia Center for Children’s Environmental Health, New York, New York; 7 Mailman School of Public Health, Columbia University, New York, New York

## Abstract

**Importance:**

Chlorpyrifos (CPF) is one of the most widely used insecticides throughout the world. Preclinical and clinical studies have suggested that prenatal CPF exposure is neurotoxic, but its effects on the human brain are unknown.

**Objective:**

To identify the associations of prenatal CPF exposure with brain structure, function, and metabolism in school-aged children.

**Design, Setting, and Participants:**

This prospective, longitudinal pregnancy cohort study was conducted from January 1998 to July 2015, with data analysis from February 2018 to November 2024 in a community in northern Manhattan and South Bronx, New York. Of 727 pregnant women of African American or Dominican descent in the original community cohort, 512 had CPF levels measured at delivery. Offspring 6 years and older were approached for magnetic resonance imaging (MRI) scanning.

**Exposure:**

Prenatal CPF exposure.

**Main Outcomes and Measures:**

Anatomical MRI measures of cortical thickness and local white matter volumes, diffusion tensor imaging indices of tissue microstructure, MR spectroscopy indices of neuronal density, arterial spin labeling measures of regional cerebral blood flow, and cognitive performance measures. Prespecified hypotheses before data collection included CPF-related structural abnormalities in frontotemporal cortices, basal ganglia, and white matter pathways interconnecting them, and reduced neuronal density.

**Results:**

Participants included 270 youths (123 boys and 147 girls) aged 6.0 to 14.7 years (mean [SD] age, 10.38 [1.12] years) with self-identified Dominican or African American mothers. Progressively higher prenatal CPF exposure levels associated significantly in childhood with progressively thicker frontal, temporal, and posteroinferior cortices; reduced white matter volumes in the same regions; higher fractional anisotropy and lower diffusivity in internal capsule white matter; lower regional blood flow throughout the brain; lower indices of neuronal density in deep white matter tracts; and poorer performance on fine motor (β, −0.30; *t*_261_ = −5.0; *P* < .001) and motor programming (β, −0.27; *t*_261_ = −4.36; *P* < .001) tasks.

**Conclusions and Relevance:**

Prenatal CPF exposure was associated with altered differentiation of neuronal tissue into cortical gray and white matter, increased myelination of the internal capsule, poorer motor performance, and profoundly impaired neuronal metabolism throughout the brain. CPF is known to increase oxidative stress and inflammation and in turn impair mitochondrial functioning, neuronal development, and maturation of the oligodendrocyte precursor cells responsible for axonal myelination. These molecular and cellular effects of CPF likely account at least in part for the observed associations of CPF with poorer long-term brain and motor outcomes.

## Key Points

**Question:**

Is prenatal exposure to the pesticide chlorpyrifos (CPF) associated with disturbances in brain development?

**Findings:**

In this cohort study, progressively higher prenatal exposure to CPF was associated significantly with progressively greater alterations in brain structure, function, and metabolism and progressively poorer measures of motor speed and motor programming in a community sample of 270 youths aged 6 to 14 years.

**Meaning:**

Prenatal CPF exposure was associated with long-term alterations in brain development and behavior.

## Teaser

This cohort study evaluates the association between brain abnormalities and prenatal exposure to chlorpyrifos.

## Introduction

Chlorpyrifos (CPF) (0,0-diethyl-0-3,5,6-trichloro-2-pyridyl phosphorothioate), a chlorinated organophosphate, is one of the most widely used insecticides throughout the world.^1^ Because it is commonly used for agricultural purposes in the US, CPF is nearly ubiquitous in nonorganic fruits, vegetables, and grains and in outdoor air and dust samples near agricultural areas.^2^ Residential use was banned in the US in 2001, although it remains common throughout the world.^3^

CPF enters the bloodstream through ingestion, skin contact, or inhalation.^4^ In pregnant women, it crosses the placenta to reach the fetal blood stream,^5,6^ where concentrations are up to 4-fold higher than in maternal tissues,^7,8^ then it crosses the fetal blood-brain barrier to enter the brain. Rodent models have shown that prenatal and neonatal exposure to CPF and other organophosphates, at exposures below the threshold for inhibition of cholinesterase that is toxic to insects, can inhibit the generation of neurons and glia, interfere with neuronal differentiation and the generation of axons and synapses, disrupt axonal transport and synaptic transmission, and promote cell death.^9,10,11,12^ CPF exposure early in development is more toxic to glia than neurons,^13,14,15,16^ initially producing excess cellular extensions and swelling, then later a diffuse astrogliosis.^13,17^ Molecular mediators of these CPF effects include oxidative stress,^18^ neuroinflammation,^19^ impaired mitochondrial functioning, and altered responses to neurotrophins.^20^

In rodent models, these molecular and cellular effects of prenatal CPF exposure in subtoxic doses produce learning and memory problems, motor hyperactivity, and anxiety- and depressive-like behaviors in adulthood.^21^ In humans, prenatal exposures to CPF and other organophosphate pesticides have been associated with fetal growth restriction and smaller head size,^22^ lower birth weight,^23^ abnormal newborn reflexes,^24,25^ and neurodevelopmental symptoms in toddlers that include autism spectrum disorder,^26,27^ inattention,^26,28^ and lower overall intelligence.^29,30,31^

Herein we report the effects of prenatal CPF exposure on long-term brain development in 270 youths aged 6 to 14 years who were drawn from our prospective, longitudinal pregnancy cohort at the Columbia Center for Children’s Environmental Health. Based on prior findings that prenatal CPF exposure affects higher-order cognitive processes (intelligence, attention, executive functioning, and socialization), our hypothesis was that prenatal CPF exposure would associate significantly with brain measures in regions that support these capacities—frontal and temporal cortices, basal ganglia, and the white matter pathways that connect them.

## Methods

### Study Design

This was a prospective, longitudinal pregnancy cohort study assessing the effects of prenatal CPF exposure on offspring brain structure, metabolism, and function in middle childhood. From January 1998 to July 2006, 727 pregnant women self-identifying as African American or Dominican and residing in northern Manhattan, New York, were recruited via community prenatal clinics.^32^ Inclusion criteria were age 18 to 35 years; registered at New York Presbyterian Medical Center or Harlem Hospital prenatal clinics by gestational week 20; no diabetes, hypertension, or HIV; and no current tobacco or illicit drug use. When beginning study recruitment in 2014, 523 remained active participants, and 512 had completed prenatal questionnaires. Magnetic resonance imaging (MRI) scanning was offered to youths aged 6 years and older who did not have MRI contraindications or metallic dental braces. MRI data were obtained in 332 children at ages 6.0 to 14.7 years, with 270 having either umbilical cord or maternal plasma CPF levels available at birth and high-quality, usable MRI data. MRI data were collected from April 2007 to July 2015, with data analysis from February 2018 to November 2024. Parents provided informed written consent; children provided assent. The institutional review boards at Columbia University and New York State Psychiatric Institute approved this study. We followed the Strengthening the Reporting of Observational Studies in Epidemiology (STROBE) reporting guideline.

### Maternal Prenatal Assessments

During pregnancy, mothers completed questionnaires on demographic characteristics, educational background, material hardship,^33^ and home environment.^34^ These characteristics were stable over follow-up (eMethods in Supplement 1). Mothers were residents in a northern Manhattan neighborhood of almost exclusively Latino and African American households. They self-identified race and ethnicity in response to 2 questions: (1) Are you Latino/Hispanic (yes/no) and (2) What is your race (American Indian/Alaska Native; Asian; Black or African American; Native Hawaiian/Other Pacific Islander; White). Race and ethnicity data were collected and reported to assess the representativeness of the sample.

### CPF Exposure

As previously described,^35^ exposure to CPF was mostly attributable to indoor residential spraying for pests, which was prevalent in this inner-city neighborhood before residential use was banned in 2001. Recruitment continued through 2006. CPF exposure levels dropped progressively after the ban, when CPF-containing products were pulled off the market and home supplies were exhausted, replaced largely by carbamate-based pesticides.

### Prenatal Exposure Estimates

Maternal blood (30-35 mL) was collected within 1 day postpartum, and umbilical cord blood (30-60 mL) was obtained at delivery. CPF plasma levels were measured at the US Centers for Disease Control and Prevention using isotope dilution gas chromatography high-resolution mass spectrometry^36^ in 2 batches, with slightly different limits of detection (LOD): 0.25 pg/g and 0.36 pg/g. Primary analyses assigned the respective LOD as the CPF exposure for those participants. Sensitivity analyses replaced LOD values with LOD/2, a common practice.^37^ In another study, CPF concentrations correlated significantly between maternal and cord plasma (*r*, 0.76; *P* < .001).^6^ If umbilical cord levels were unavailable, maternal plasma values were substituted using the regression: ln(newborn CPF) = 0.46 + 0.61 × ln(maternal CPF). We also estimated prenatal exposure to 2 forms of air pollution: (1) polycyclic aromatic hydrocarbon (PAH) in the third trimester and (2) average daily particulate matter of 2.5 µm or less (PM_2.5_) exposure throughout pregnancy (eMethods in Supplement 1).

### Youth Behavioral Assessments

Youths underwent a psychometric assessment at the time of MRI scanning. Domains included sensorimotor, visuomotor, and visuospatial functioning, motor programming, fine and gross motor dexterity, attention, impulsivity, cognitive flexibility, and general intelligence. Mothers and youths completed surveys of youths’ social, emotional, and behavioral symptoms (eMethods in Supplement 1).

### Brain Imaging

Anatomical MRI evaluated cortical thickness and local white matter volumes at the cerebral surface. Diffusion tensor imaging (DTI) measured the direction and rate of water diffusion, which are influenced by tissue microstructure. DTI scalar indices included fractional anisotropy (FA), representing the directional preference of diffusion, and average diffusion coefficient (ADC), representing the average rate of diffusion over 3 spatial directions. Arterial spin labeling (ASL) measured regional cerebral blood flow (rCBF). Magnetic resonance spectroscopic imaging (MRSI) measured brain metabolite concentrations, including *N*-acetyl-l-aspartate (NAA), an index of healthy neuron density (eFigure 1 in Supplement 1).

Detailed pulse sequences, quality control, and image processing methods are in the eMethods in Supplement 1. Investigators and technicians evaluating MRI and behavioral data were blind to CPF exposure levels and participant characteristics.

### Statistical Analyses

#### Covariates in All Models

All analyses covaried for child age at the time of scan (eFigure 2 in Supplement 1), sex, race and ethnicity (self-identified Dominican Hispanic or African American), maternal education (completed high school or not), material hardship during pregnancy, and home environmental stress (eMethods in Supplement 1). Bivariate correlations for covariates are in eTable 1 in Supplement 1.

#### Exposure Associations With Brain Measures

To test a priori hypotheses, we applied a general linear model at each voxel of the brain template, in each imaging modality separately:

Imaging measure = β_0_ + β_1_ × CPF + β_2_ × age + β_3_ × sex + β_4_ × race/ethnicity + β_5_ × maternal education + β_6_ × material hardship + β_7_ × home stress + ε

**Figure 1.**
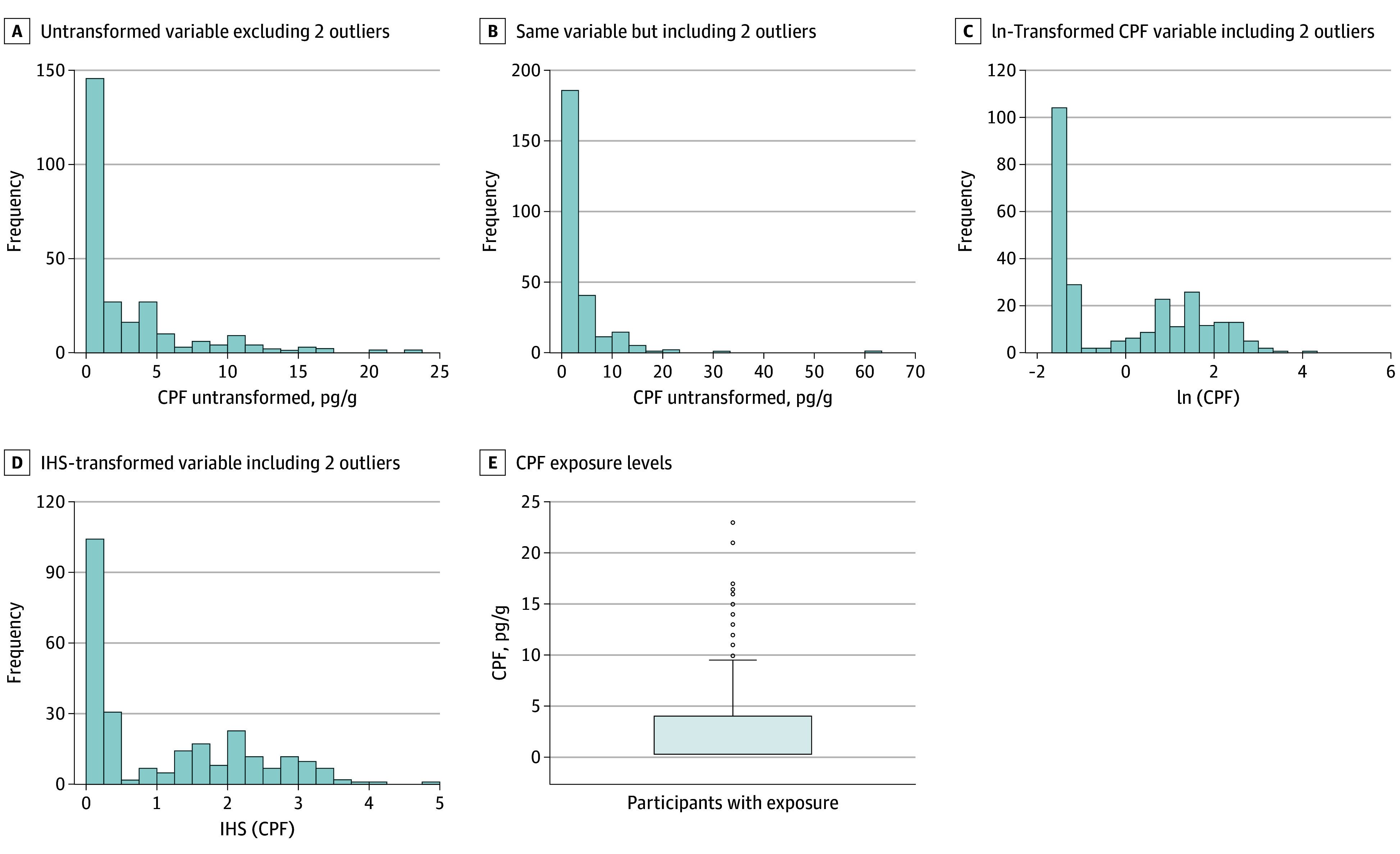
Distributions of Chlorpyrifos (CPF) Exposure Variables The transformed variables were used in sensitivity analyses. The 2 outliers in panel D were CPF levels of 32 and 63 pg/g, 5 and 10.4 SD from the mean, respectively. The data in D were used for all primary analyses. IHS indicates inverse hyperbolic sine.

The dependent imaging measure variable for anatomical analyses was cortical thickness or local white matter volume. For DTI, it was FA or ADC in white matter (eMethods in Supplement 1). For ASL, it was rCBF, and for MRSI, it was NAA concentration. The independent variable was the untransformed CPF exposure estimate (Figure 1). Two youths with outlier values (5 and 10.4 SD from the mean) were excluded from primary analyses.

#### Sensitivity Analyses

We included as additional covariates: (1) ln-transformed PAH and PM_2.5_ values to account for air pollution effects and (2) maternal age. We also replaced LOD values with LOD/2. We included the 2 participants with outlier CPF values but reduced outlier influences using either ln-transformed or inverse hyperbolic sine (IHS)–transformed^38^ CPF values.

#### Moderating Effects

We assessed whether participant age or sex moderated associations of CPF exposure with brain measures by testing significance of age × exposure and sex × exposure interactions.

#### Associations of Prenatal Exposures With Behavioral Outcomes

We assessed these using the following regression:

Behavioral measure = β_0_ + β_1_ × CPF + β_2_ × age + β_3_ × sex + β_4_ × race/ethnicity + β_5_ × maternal education + β_6_ × material hardship + β_7_ × home stress + ε

Given the numerous statistical tests, we considered 2-tailed *P* values less than .0005 significant.

#### Brain-Based Mediation

At each brain voxel, we used 3 regression equations to assess whether MRI measures (M) mediated significant associations of CPF levels (X) with behavioral scores (Y): (1) Y = c_1_X + e_1_, where c_1_ is the total effect; (2) M = αX + e_2_; and (3) Y = c_2_X + bM + e_3_, where c_2_ is the direct effect of X with Y and α × b is the indirect effect of X on Y, mediated by M. Statistical significance of the mediated effect α × b was assessed using the Sobel test (eMethods in Supplement 1).

#### Correction of MRI Statistical Maps for Multiple Comparisons

We used a parametric, cluster size-based familywise error rate (FWER) with a cluster-defining threshold of 2.0 to correct for multiple comparisons across all image voxels^39^(eMethods in Supplement 1).

#### Connectomics

We explored associations of CPF exposure with structural and functional connectivity using graph theoretical (GT) measures (eMethods in Supplement 1). For DTI, these included density, local efficiency, diameter, small worldness, characteristic path length, average clustering coefficient, and rich club-k25 (eTable 2 in Supplement 1). For resting-state functional MRI (rs-fMRI), these included community structure, density, global efficiency, diameter, radius, characteristic path length, and transitivity (eTable 3 in Supplement 1). Linear regression in SPSS. version 21 (IBM) assessed associations with each GT measure entered separately as the dependent variable, CPF level the independent variable, and covariates listed above.

## Results

### Participants

These were 270 youths (123 boys and 147 girls) aged 6.0 to 14.7 years (mean [SD] age, 10.38 [1.12] years) who had prenatal CPF levels and at least 1 usable MRI modality (264 anatomical of 270 attempted; 204 DTI of 244 attempted; 176 ASL of 210 attempted; 213 MRSI of 235 attempted;168 rs-fMRI of 233 attempted). Mothers self-identified as Dominican or African American. They had similar demographic profiles, and their offspring had similar long-term cognitive outcomes, as others in the cohort (Table).

**Table.** Sociodemographic Characteristics of the Study Sample

Characteristic^a^	Study sample scanned^b^		All other original cohort members not scanned^c^		Comparison	
	No.	Mean (SD)	No.	Mean (SD)	Test statistic	*P* value
Maternal education, y	270	12.9 (2.36)	455	12.66 (2.91)	*t*_723_ = −1.34	.18
Maternal age	270	25.4 (5.11)	456	25.02 (4.84)	*t*_724_ = −1.01	.32
Quality of home environment	270	39.5 (6.05)	456	39.27 (5.28)	*t*_724_ = −0.53	.60
Child age at MRI scan	270	10.38 (1.12)	NA	NA	NA	NA
WISC						
Verbal comprehension	261	96.64 (11.80)	212	95.81 (13.30)	*t*_471_ = −0.72	.47
Perceptual reasoning	261	97.94 (13.44)	212	97.07 (14.14)	*t*_471_ = −0.69	.49
Working memory	261	98.81 (12.94)	212	97.63 (14.15)	*t*_471_ = −0.95	.34
Processing speed	261	94.44 (13.40)	212	92.09 (12.89)	*t*_471_ = −1.92	.06
Full scale IQ	261	96.42 (12.36)	212	94.84 (13.72)	*t*_471_ = −1.31	.19
Prenatal chlorpyrifos, pg/g	270	3.11 (5.70)	241	2.45 (3.95)	*t*_509_ = −1.53	.13
Prenatal PAH, ng/m^3^	260	3.35 (4.05)	426	3.52 (7.79)	*t*_684_ = 0.34	.74
Maternal race and ethnicity, No. (%)^d^	270	NA	456	NA	χ^2^_1_ = 2.36	.13
African American	104	(38.5)	150	(32.9)	NA	NA
Dominican	166	(61.5)	306	(67.1)	NA	NA
Child sex, No. (%)	270		456			
Male	123	(45.5)	227	(50)	χ^2^_1_ = 1.21	.27
Female	147	(54.5)	229	(50)		
Handedness, No. (%)	262		127			
Right dominant	236	(90.0)	115	(90.6)	χ^2^_1_ = 0.02	.88
Left dominant	26	(10.0)	12	(9.4)		
Material hardship, No. (%)	270		456			
0 Unmet needs	162	(60.0)	275	(60.3)	χ^2^_2_ = 4.64	.10
1 Unmet need	57	(21.1)	72	(15.8)		
>2 Unmet needs	51	(18.9)	109	(23.9)		
Prenatal ETS in the home, No. (%)	270	NA	456	NA	NA	NA
Yes	92	(34.1)	172	(37.7)	χ^2^_1_ = 0.97	.32
No	178	(65.9)	284	(62.3)		

Abbreviations: ETS, environmental tobacco smoke; MRI, magnetic resonance imaging; NA, not applicable; PAH, polycyclic aromatic hydrocarbons; WISC, Wechsler Intelligence Scale for Children.

^a^ The measures are described in detail in the eMethods in Supplement 1.

^b^ Participants aged 6 to 14.7 years who had chlorpyrifos levels and usable data in at least 1 MRI modality compared to all other participants in the original parent cohort.

^c^ Participants did not undergo imaging either because they had not reached the eligible age window during the MRI study period, they were no longer cohort members at the time of the MRI study, or they declined MRI scanning.

^d^ Race and ethnicity data were collected via self-report and reported because to assess the representativeness of the sample.

### Anatomical MRI

**Figure 2.**
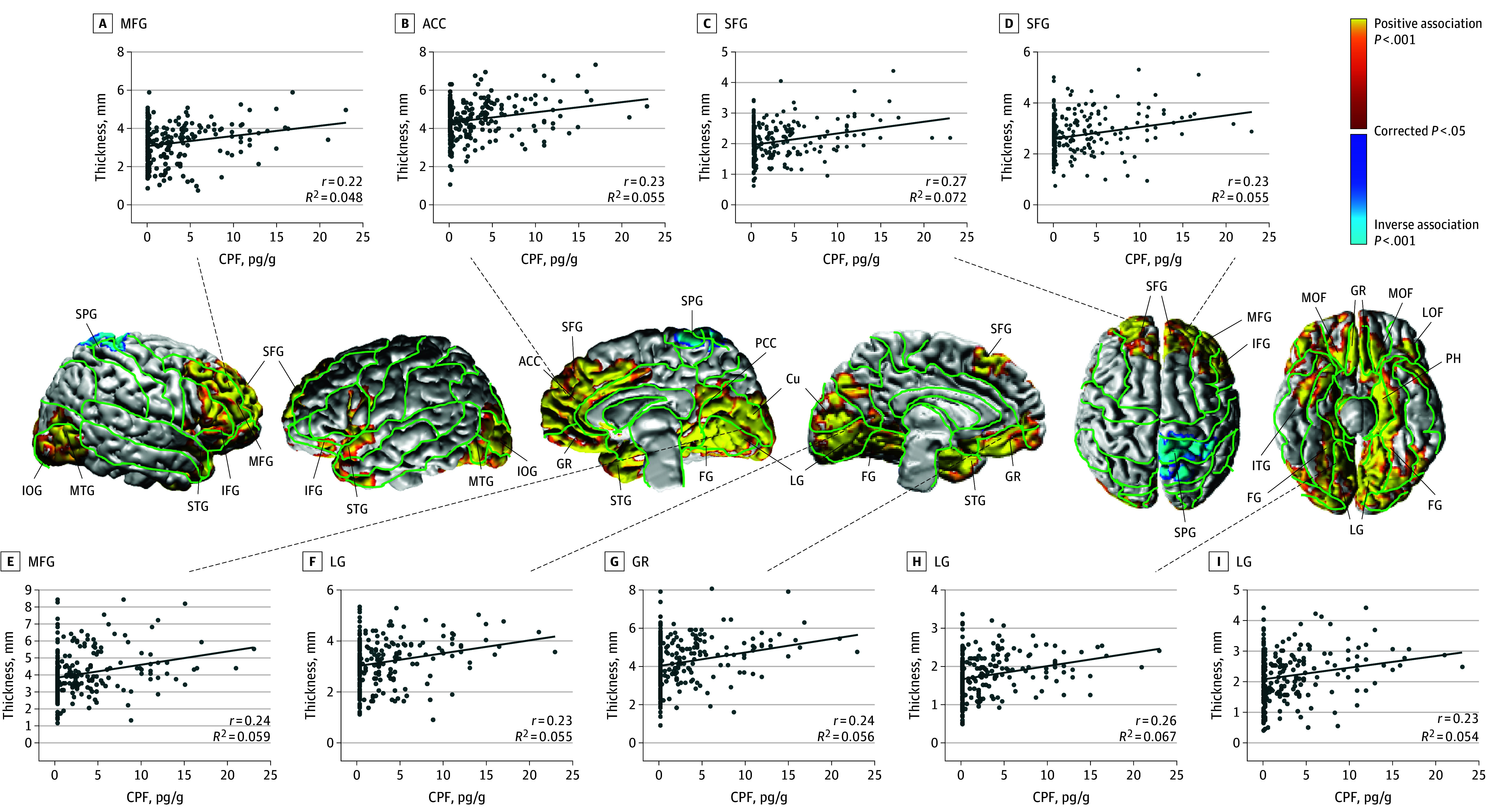
Statistical Maps for Prenatal Chlorpyrifos (CPF) Exposure Associations With Cortical Thickness A regression model tested exposure effects at each point on the cerebral surface: cortical thickness = β_0_ + β_1_ × cpf + β_2_ × age + β_3_ × sex + β_4_ × ethnicity + β_5_ × maternal education + β_6_ × material hardship + β_7_ × home stress + ε. The number of participants for this analysis was 262 (mean [SD] age, 10.87 [1.07] years; 120 boys and 142 girls). The statistical significance (cluster size familywise error rate [FWER]–corrected *P* values) of the associations of exposure with cortical thickness at each point on the surface of the brain are color-coded. Only *P* values that survived cluster-size FWER correction are plotted. Views of the brain, shown left to right, are right lateral, left lateral, right mesial, left mesial, dorsal, and ventral. Anatomical magnetic resonance imaging (MRI) measures were sampled at representative points as indicated, and scatterplots for the association of CPF exposure with those measures are shown for each sampled point. Anatomical measures were adjusted for participant age at MRI scan, sex, race, ethnicity, maternal education, material hardship during pregnancy, and home stress at child age 3 years. ACC indicates anterior cingulate cortex; Cu, cuneus; FG, fusiform gyrus; GR, gyrus rectus; IFG, inferior frontal gyrus; IOG, inferior occipital gyrus; IPL, inferior parietal lobule; ITG, inferior temporal gyrus; LOF, lateral orbitofrontal gyrus; LG, lingual gyrus; MFG, middle frontal gyrus; MOF, middle orbitofrontal gyrus; MTG, middle temporal gyrus; PCC, posterior cingulate cortex; SFG, superior frontal gyrus; SPG, superior parietal gyrus; STG, superior temporal gyrus.

CPF exposure associated significantly and positively with cortical thickness in frontal regions (superior frontal gyrus [SFG], middle frontal gyrus [MFG], inferior frontal gyrus [IFG], anterior cingulate cortex [ACC], gyrus rectus [GR], middle orbitofrontal gyrus [MOF], lateral orbitofrontal gyrus [LOF]), temporal regions (superior temporal gyrus [STG], middle temporal gyrus [MTG], inferior temporal gyrus [IFT], parahippocampus [PH]), and posteroinferior regions (posterior cingulate cortex [PCC], cuneus [Cu], inferior occipital gyrus [IOG], lingual gyrus [LG], fusiform gyrus [FG]), and inversely with cortical thickness in the dorsal parietal region (superior parietal gyrus [SPG]) (Figure 2). CPF exposure also associated inversely with local white matter volumes in frontal regions (ACC, MOF, and GR), temporal regions (STG and PH), and posteroinferior regions (inferior parietal lobule [IPL], PCC, Cu, FG, and LG) (eFigure 3 in Supplement 1).

### DTI

**Figure 3.**
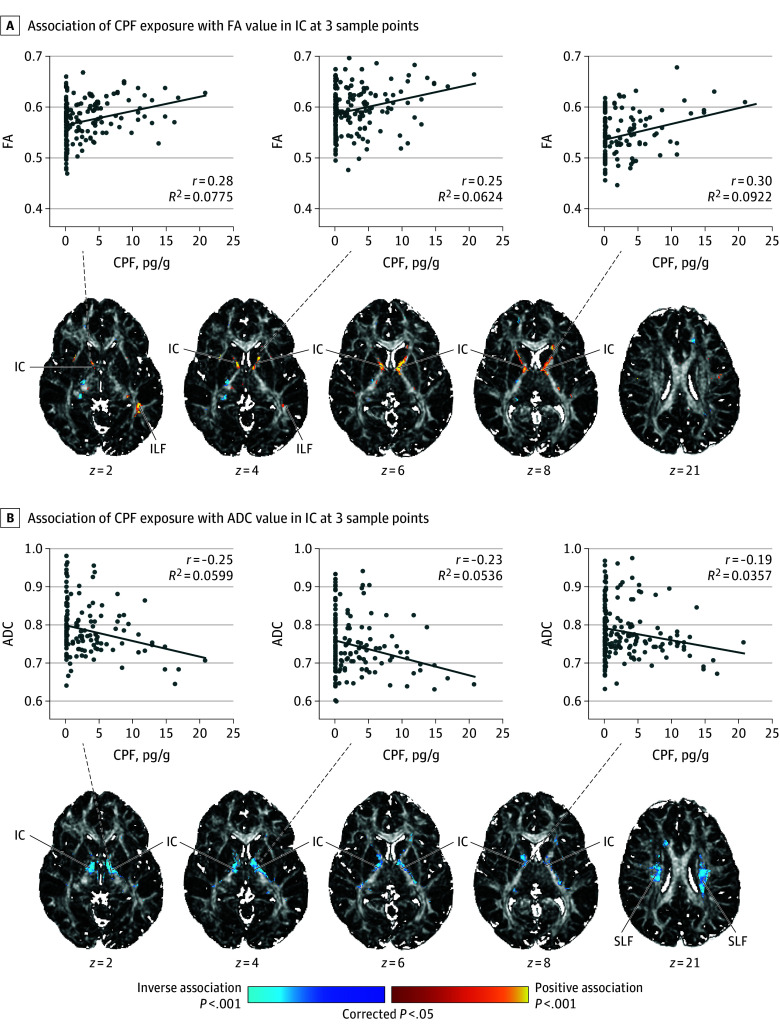
Statistical Map of Chlorpyrifos (CPF) Prenatal Exposure Effects on Diffusion Tensor Imaging (DTI) Measures in White Matter The regression model that tested CPF exposure effects at each white matter voxel was DTI measure = β_0_ + β_1_ × CPF + β_2_ × age + β_3_ × sex + β_4_ × race/ethnicity + β_5_ × maternal education + β_6_ × material hardship + β_7_ × home stress + ε, with DTI measure either fractional anisotropy (FA) or average diffusion coefficient (ADC). The statistical significance (cluster size familywise error rate [FWER]–corrected *P* values) of the associations of exposure with cortical thickness at each point on the surface of the brain are color-coded. Only *P* values that survived cluster-size FWER correction are plotted. The number of participants in this analysis was 202 (mean [SD] age, 10.78 [1.33] years; 89 boys and 113 girls). The *z* values below each column represent the *z* coordinate in Talairach space. FA and ADC values were sampled at representative points, and scatterplots for the association of CPF exposure with the DTI values at those points are shown. DTI values are adjusted for participant age at magnetic resonance imaging scan, sex, race, ethnicity, maternal education, material hardship during pregnancy, and home stress at child age 3 years. IC indicates internal capsule; ILF, inferior longitudinal fasciculus; SLF, superior longitudinal fasciculus.

CPF exposure associated positively with FA and inversely with ADC values in the internal capsule [IC], especially its anterior limb and genu for FA and genu and posterior limb for ADC (Figure 3; eFigures 4-5 in Supplement 1). Reduced ADC values derived from reduced diffusion in both the axial and radial directions of the long axis of IC fiber bundles (eFigure 6 in Supplement 1).

### ASL

**Figure 4.**
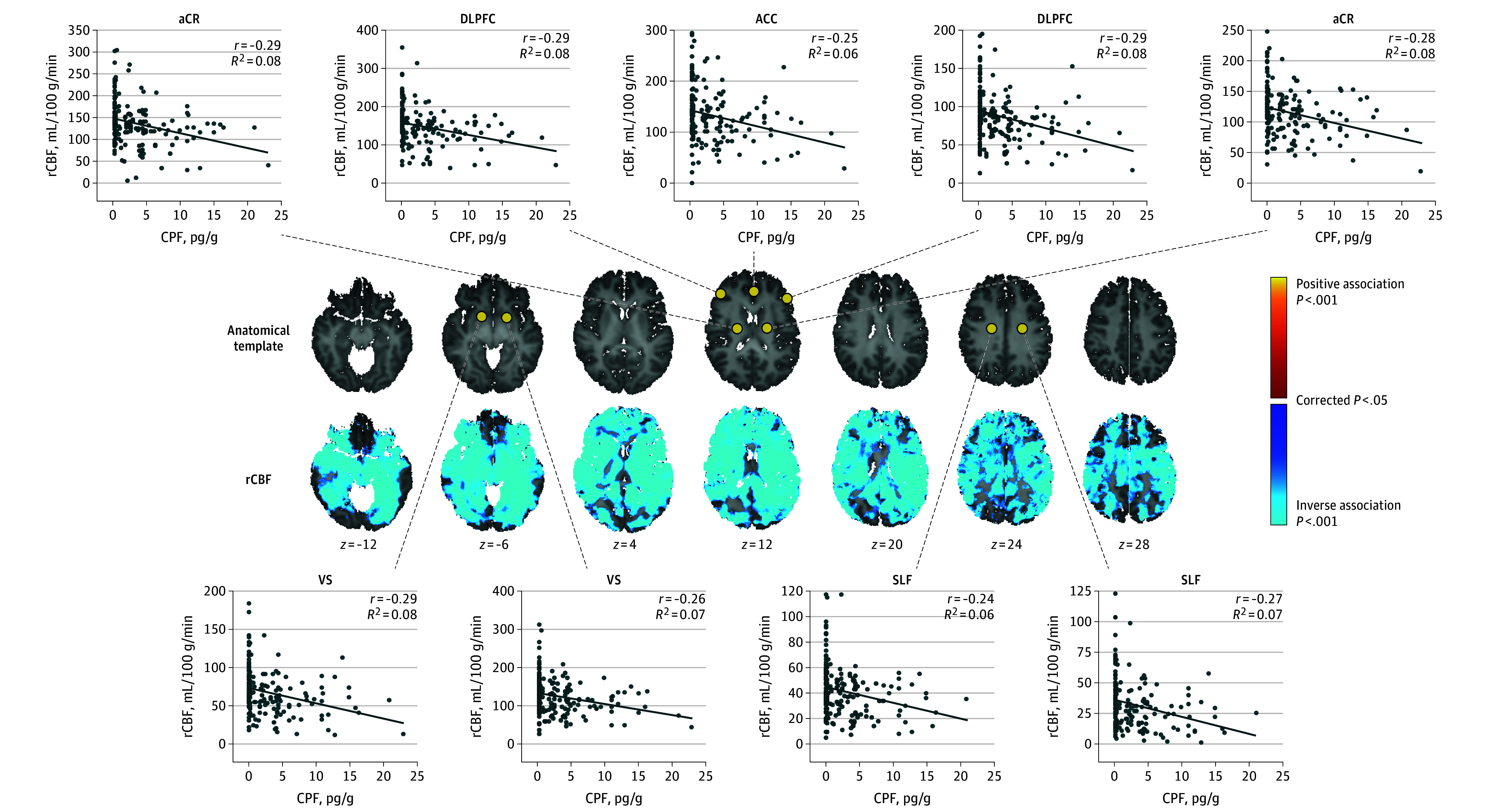
Statistical Maps of Prenatal Chlorpyrifos (CPF) Exposure Associations With Arterial Spin Labeling (ASL) Regional Cerebral Blood Flow (rCBF) Measures The regression model that tested CPF exposure effects voxelwise was rCBF = β_0_ + β_1_ × CPF + β_2_ ×age + β_3_ × sex + β_4_ × race/ethnicity + β_5_ × maternal education + β_6_ × material hardship + β_7_ × home stress + ε. The statistical significance (cluster size familywise error rate [FWER]–corrected *P* values) of the associations of exposure with cortical thickness at each point on the surface of the brain are color-coded. Only *P* values that survived cluster-size FWER correction are plotted. The number of participants for ASL was 175 (mean [SD] age, 10.89 [1.24] years; 75 boys and 100 girls). The *z* values below each column represent the *z* coordinate in Talairach space. rCBF data were sampled at representative points. Scatterplots showing the association of CPF exposure with those measures are shown for those points. rCBF values are adjusted for participant age at magnetic resonance imaging scan, sex, race, ethnicity, maternal education, material hardship during pregnancy, and home stress at child age 3 years. Because the significant associations for rCBF involved so much of the brain, we show the T1-weighted anatomical template brain for the corresponding slices and the locations where we sampled the ASL data immediately above the statistical maps. aCR indicates anterior corona radiata; IFO, inferior fronto-occipital fasciculus; Ins, insula; PTR, posterior thalamic radiation; SLF, superior longitudinal fasciculus.

CPF exposure associated inversely with rCBF values across most brain regions (Figure 4; eFigure 7 in Supplement 1).

### MRSI

CPF exposure associated inversely with NAA concentrations in scattered foci within deep white matter tracts (inferior frontal-occipital fasciculus [IOF], posterior thalamic radiation [PTR], anterior corona radiata [aCR]) and in gray matter of the insular cortex [Ins] (eFigures 8-9 in Supplement 1). Findings for other metabolites were sparse (eFigure 10 in Supplement 1). When normalizing metabolite concentrations to creatine, CPF associated positively with Glx concentrations in the anterior cingulate cortex (eFigure 11 in Supplement 1).

### Moderation Effects

Neither age nor sex significantly moderated the association of CPF with MRI measures in any modality (not shown).

### Behavioral Outcomes

CPF values associated significantly and inversely with fine motor speed (finger-tapping: β, −0.30; *t*_261_ = −5.0; *P* < .0001) and motor programming (finger-sequencing: β, −0.27; *t*_261_ = −4.36; *P* < .0001) in both hands, disproportionately in the nondominant hand (β, −0.13; *t*_261_ = −2.10; *P* < .04) and disproportionately for motor programming (β, −0.16; *t*_261_ = −2.57; *P* < .01) (eFigure 12 in Supplement 1). No other behavioral or symptom measures met the significance threshold (eTables 4-5 in Supplement 1).

### Sensitivity Analyses

Covarying for PM_2.5_ and PAH concurrently (eFigures 13-15 in Supplement 1) or for maternal age (eFigures 16-19 in Supplement 1) had negligible influences on our findings, as did use of LOD/2 (eFigures 20-23 in Supplement 1) or ln- and IHS-transformed CPF values that included outliers (eFigures 24-27 in Supplement 1**)**.

### Mediation

Brain measures did not significantly mediate CPF associations with motor speed or programming.

### Connectomics

No DTI or rs-fMRI GT measure associated significantly with CPF exposure (eTable 6 in Supplement 1).

## Discussion

In this cohort study, progressively higher prenatal CPF exposure levels associated significantly with progressively greater alterations in brain measures in each MRI modality, suggesting that prenatal exposure may produce enduring disturbances in brain structure, function, and metabolism in direct proportion to exposure level. Sensitivity analyses showed that including 2 exposure outliers and controlling for prenatal exposure to air pollution had minimal influence on our findings. CPF associations with MRI measures did not vary significantly with participant age or sex. Progressively greater CPF exposure associated significantly with progressively poorer fine motor and motor programming skills, though brain measures did not significantly mediate these associations.

### Cortical Thickness and White Matter Surface

Progressively higher CPF exposures associated with progressively thicker frontal, temporal, and posteroinferior cortices and thinner dorsoparietal cortex. Higher exposure also associated with progressively smaller local white matter volumes in locations generally similar to cortical thickening, suggesting that prenatal CPF exposure may have shifted the gray-white matter interface, possibly via either exposure-related changes in lamination of the cortex or altered myelination of underlying white matter axons.^40^

Frontal thickening included dorsolateral prefrontal, anterior cingulate, and orbitofrontal cortices, which support working memory, sustained attention, executive functioning, and cognitive flexibility. Posterior thickening included the cuneus and posterior cingulate, lingual, fusiform, and parahippocampal gyri, which support self- and other-awareness, autobiographical and episodic memory, and high-level visual processing.

#### Tissue Microstructure

Progressively higher CPF exposure associated with higher FA and lower ADC values in the internal capsule, likely representing either increased axon packing density or increased myelination of IC axons.^41,42^ These fibers interconnect frontal and motor cortices with the basal ganglia, thalamus, cranial nerve nuclei, and spinal cord to support higher-order executive functions and movements of the head, face, and upper and lower extremities.^43,44^

#### Blood Flow

Progressively higher prenatal CPF exposure levels associated with progressively lower rCBF values across most gray and white matter regions, suggesting that CPF exposure is associated with widespread and longstanding reductions in brain metabolism. Preclinical studies have shown that CPF exposure produces widespread reductions in adenylyl cyclase signaling,^45^ impairs mitochondrial functioning,^46^ and reduces neurotransmitter signaling,^47^ all of which would reduce metabolism and blood flow.

#### Neuronal Density

Higher CPF exposure levels associated with lower NAA concentrations in scattered foci within deep white matter and insular cortex gray matter. NAA is present primarily in neuronal mitochondria and is considered an index of healthy neuron density.^48,49,50^ Lower neuronal density in white matter suggests that the observed lower ADC values (and higher FA) likely represent excess myelination, not higher axon packing density.

#### Behavior

Prenatal CPF levels associated significantly with poorer fine motor and motor planning scores, consistent with CPF-related DTI disturbances in motor circuits. The paucity of CPF-associated findings in other behavioral domains, despite the large spatial expanse of MRI findings, suggests that MRI is more sensitive than behavioral measures for detecting exposure-related brain disturbances. Behavioral measures are inherently noisy, varying with participant attention, motivational state, and practice, and they are shaped by myriad past environmental and experiential influences. Moreover, MRI measures may be more specific in localizing effects within the brain, whereas behavioral measures comprise multiple subprocesses and do not map onto a single brain region or single neural circuit. Additionally, the cognitive and behavioral processes supported by brain regions associated with CPF exposure have not been measured extensively or at all in our study or prior studies.

#### Model of Pathogenesis

In this same cohort, we previously reported remarkably similar findings in association with prenatal exposure to air pollution^51^ (a shift of cortical gray-white boundary, increased FA and lower ADC values in the IC, and widespread reduced rCBF). However, the CPF and air pollution findings are independent of one another: correlation of exposure values was negligible (*r* < 0.03), and CPF findings were unchanged when covarying for PM_2.5_ and PAH exposures.

Similar findings across neurotoxicants with widely differing chemical compositions suggests that a final common pathway mediates their effects on brain development. Preclinical studies show that inflammation and oxidative stress mediate many of the cellular effects of CPF,^52^ PM_2.5_,^53^ and PAH.^54^ Inflammation and oxidative stress during fetal brain development impair mitochondrial functioning,^55,56,57^ which in turn produces more inflammation and oxidative stress, creating a vicious cycle of inflammation and metabolic dysfunction.^55,58^ Enduring mitochondrial damage would account for widespread blood flow reductions observed across all three exposures, and it would be consistent with the lower neuronal density detected in deep white matter.

Inflammation, oxidative stress, and mitochondrial dysfunction are highly toxic to preoligodendrocytes,^58,59^ which degenerate and trigger compensatory proliferation of oligodendrocyte progenitors to supernormal levels, though many new preoligodendrocytes fail to differentiate into myelinating oligodendrocytes (eDiscussion in Supplement 1). An excess number of myelinating oligodendrocytes could produce the higher FA and lower ADC values we observed in the IC and deep white matter. Dysmyelination can further disrupt axonal maturation and activity-dependent maturation of cortical gray matter,^55,59^ which could in turn produce the altered gray-white matter interface we observed. Consistent with this proposed model of pathogenesis, a prior study showed that CPF administered to developing rats during the peak of glial cell replication (PN11-14) reduced brain-side glial cell densities, followed by a subsequent rebound in glial cells by PN30.^60^ Similarly, CPF exposure in newborn rats (PN11-14) activated inflammatory pathways, increased microglia and astrocytes in the substantia nigra, and reduced dopaminergic neuron numbers in adulthood.^61^ CPF-induced inflammation and oxidative stress likely also have direct toxic effects on neuronal development, either by killing neurons or disrupting their proliferation, differentiation, and apoptosis.^55,62^

### Clinical Implications

Scatterplots were without evidence of an exposure threshold below which associations were absent. The CPF associations reported here are likely to be shared by other organophosphates, which have similar molecular and cellular effects,^63,64,65^ and perhaps by a wide range of other chemically diverse neurotoxicants that produce neuroinflammation and oxidative stress,^66^ thus offering a potential future therapeutic target for reducing or preventing their adverse effects.^67,68,69^ Because the effects of prenatal CPF and air pollution exposures are independent and additive within the same neural systems, efforts to limit exposure to one should include efforts to limit exposure to the other.

### Limitations

This study has limitations. Because our sample comprised urban Dominican and African American women and their offspring, our findings may not generalize entirely to other populations. Selection bias was possible in terms of which participants of our cohort we succeeded in contacting and who agreed to participate with MRI scanning, although participants in this study were comparable to the rest of the cohort in terms of sociodemographic characteristics and long-term cognitive outcomes, suggesting that selection bias was limited. We did not measure or control for postnatal CPF exposure^70^ and cannot exclude the possibility that it contributed to our findings. We also did not measure exposure to other insecticides that can co-occur with CPF exposure, nor did we assess the moderating effects of genetic polymorphisms that influence the rates of organophosphate metabolism.^30,71,72^ Additionally, this is a naturalistic observational study of associations that may not represent causation.

## Conclusions

Prenatal CPF exposure was associated with altered differentiation of neuronal tissue into cortical gray and white matter, increased myelination of the internal capsule, brainwide impaired metabolism, and poor motor performance that endured into late childhood and early adolescence, likely as a result of CPF-induced oxidative stress and inflammation. Because other pesticides also induce oxidative stress and inflammation, minimizing prenatal and early life exposure to these chemicals is likely important for optimal childhood brain development.

## Notes

Supplement 1. eMethods eDiscussion eTable 1. Correlation Matrix for Model Variables eTable 2. Correlation Matrix for DTI Graph Theoretical Measures eTable 3. Correlation Matrix for rs-fMRI Graph Theoretical Measures eTable 4. Association of CPF Exposure with Neuropsychological Test Scores eTable 5. Associations of CPF Exposure with Symptom Severity Scores eTable 6. Association of CPF Exposure Levels with DTI and rs-fMRI Graph Theoretical Measures eFigure 1. Image Quality in Each MRI Modality eFigure 2. Age Distributions by Participant Sex eFigure 3. Statistical Maps of Prenatal CPF Exposure Associations with White Matter Surface eFigure 4. CPF Associations with FA Values -- All Slices for Tissue-Specific White Matter Maps eFigure 5. CPF Associations with ADC Values -- All Slices for Tissue-Specific White Matter Maps eFigure 6. CPF Prenatal Exposure Effects on Axial and Radial Diffusivity in White Matter rCBF eFigure 7. All Slices for CPF Exposure Associations with rCBF eFigure 8. Statistical Map of Prenatal CPF Exposure Associations with NAA Levels eFigure 9. All Slices for CPF Exposure Associations with NAA Levels eFigure 10. Association of Prenatal CPF Levels with All Metabolite Concentrations eFigure 11. Associations with Metabolite Concentrations Normalized to Creatine eFigure 12. Scatterplot for FingerTapping Task eFigure 13. Anatomical Measures eFigure 14. DTI and rCBF Measures eFigure 15. MRS Metabolites eFigure 16. Anatomical MRI eFigure 17. DTI Measures eFigure 18. rCBF eFigure 19. Metabolite Concentrations eFigure 20. Anatomical MRI eFigure 21. DTI Measures eFigure 22. rCBF eFigure 23. Metabolite Concentrations eFigure 24. Cortical Thickness and White Matter Measures eFigure 25. DTI Measures eFigure 26. rCBF eFigure 27. Metabolite Concentrations

Supplement 2. Data sharing statement
