# Supplementary material for: Brain Abnormalities in Children Exposed Prenatally to the Pesticide Chlorpyrifos
Source: JAMA Neurol. 2025 Aug 18;82(10):1057–68. doi: 10.1001/jamaneurol.2025.2818 (PMC12362277; doi:10.1001/jamaneurol.2025.2818)
Supplement: Supplement 1. — eMethods eDiscussion eTable 1. Correlation Matrix for Model Variables eTable 2. Correlation Matrix for DTI Graph Theoretical Measures eTable 3. Correlation Matrix for rs-fMRI Graph Theoretical Measures eTable 4. Association of CPF Exposure with Neuropsychological Test Scores eTable 5. Associations of CPF Exposure with Symptom Severity Scores eTable 6. Association of CPF Exposure Levels with DTI and rs-fMRI Graph Theoretical Measures eFigure 1. Image Quality in Each MRI Modality eFigure 2. Age Distributions by Participant Sex eFigure 3. Statistical Maps of Prenatal CPF Exposure Associations with White Matter Surface eFigure 4. CPF Associations with FA Values -- All Slices for Tissue-Specific White Matter Maps eFigure 5. CPF Associations with ADC Values -- All Slices for Tissue-Specific White Matter Maps eFigure 6. CPF Prenatal Exposure Effects on Axial and Radial Diffusivity in White Matter rCBF eFigure 7. All Slices for CPF Exposure Associations with rCBF eFigure 8. Statistical Map of Prenatal CPF Exposure Associations with NAA Levels eFigure 9. All Slices for CPF Exposure Associations with NAA Levels eFigure 10. Association of Prenatal CPF Levels with All Metabolite Concentrations eFigure 11. Associations with Metabolite Concentrations Normalized to Creatine eFigure 12. Scatterplot for FingerTapping Task eFigure 13. Anatomical Measures eFigure 14. DTI and rCBF Measures eFigure 15. MRS Metabolites eFigure 16. Anatomical MRI eFigure 17. DTI Measures eFigure 18. rCBF eFigure 19. Metabolite Concentrations eFigure 20. Anatomical MRI eFigure 21. DTI Measures eFigure 22. rCBF eFigure 23. Metabolite Concentrations eFigure 24. Cortical Thickness and White Matter Measures eFigure 25. DTI Measures eFigure 26. rCBF eFigure 27. Metabolite Concentrations [file jamaneurol-e252818-s001.pdf]

Supplemental Online Content

Peterson BS, Delavari S, Bansal R, et al. Brain abnormalities in children exposed prenatally to the pesticide chlorpyrifos. *JAMA Neurol*. Published online August 18, 2025. doi:10.1001/jamaneurol.2025.2818

eMethods

eDiscussion

eTable 1. Correlation Matrix for Model Variables

eTable 2. Correlation Matrix for DTI Graph Theoretical Measures

eTable 3. Correlation Matrix for rs-fMRI Graph Theoretical Measures

eTable 4. Association of CPF Exposure with Neuropsychological Test Scores

eTable 5. Associations of CPF Exposure with Symptom Severity Scores

eTable 6. Association of CPF Exposure Levels with DTI and rs-fMRI Graph Theoretical Measures

eFigure 1. Image Quality in Each MRI Modality

eFigure 2. Age Distributions by Participant Sex

eFigure 3. Statistical Maps of Prenatal CPF Exposure Associations with White Matter Surface

eFigure 4. CPF Associations with FA Values -- All Slices for Tissue-Specific White Matter Maps

eFigure 5. CPF Associations with ADC Values -- All Slices for Tissue-Specific White Matter Maps

eFigure 6. CPF Prenatal Exposure Effects on Axial and Radial Diffusivity in White Matter rCBF

eFigure 7. All Slices for CPF Exposure Associations with rCBF

eFigure 8. Statistical Map of Prenatal CPF Exposure Associations with NAA Levels

eFigure 9. All Slices for CPF Exposure Associations with NAA Levels

eFigure 10. Association of Prenatal CPF Levels with All Metabolite Concentrations

eFigure 11. Associations with Metabolite Concentrations Normalized to Creatine

eFigure 12. Scatterplot for FingerTapping Task

© 2025 Peterson BS et al. *JAMA Neurology*.

eFigure 13. Anatomical Measures

eFigure 14. DTI and rCBF Measures

eFigure 15. MRS Metabolites

eFigure 16. Anatomical MRI

eFigure 17. DTI Measures

eFigure 18. rCBF

eFigure 19. Metabolite Concentrations

eFigure 20. Anatomical MRI

eFigure 21. DTI Measures

eFigure 22. rCBF

eFigure 23. Metabolite Concentrations

eFigure 24. Cortical Thickness and White Matter Measures

eFigure 25. DTI Measures

eFigure 26. rCBF

eFigure 27. Metabolite Concentrations

This supplemental material has been provided by the authors to give readers additional information about their work.

© 2025 Peterson BS et al. *JAMA Neurology*.

SUPPLEMENTAL METHODS **Page #**
Assessment of Maternal Characteristics....3
Additional Prenatal Exposure Estimates
Geospatial Modeling-Based Estimates of Prenatal PM2.5 Exposure....3
Personalized Measures of Prenatal PAH Exposure....3
Cotinine Levels....3
Exposure to Second-Hand Tobacco Smoke....4
Youth Assessments
Neuropsychological Tests....4
Mental Health Symptom Ratings....4
MRI Scanning....4
MRI Pulse Sequences....4
Image Processing Methods....5
Anatomical Image Processing....5
Quality Control....5
Preprocessing....5
Cortical Thickness Measurement....6
Deformation-Based Measures of Brain Morphology....6
Overview....6
Spatial Co-Registration....6
Identification of Point Correspondences Across Brain Surfaces....6
Calculation of Distances Used in Statistical Modeling
Selection of the Template Brain....6
Consideration of Alternative Deformation-Based Metrics....7
DTI Processing....7
Quality Control....7
Maps of DTI Scalar Indices....7
Spatial Normalization of DTI Maps....8
Tissue-Specific Maps....8
MPCSI Processing....8
Quality Control....8
Signal Processing....8
Normalization of Metabolite Levels....8
Correcting Partial Volume Effects....9
Spatial Normalization of MPCSI Datas....9
Effects of Saturation Bands....9
Processing of Arterial Spin Labeling Data....10
Quality Control....10
Resting-State fMRI Processing....10
Quality Control....10

SUPPLEMENTAL STATISTICAL ANALYSES....10
Sensitivity Analysis Using the Inverse Hyperbolic Sine Transformation....10
Statistical Parametric Mapping and Multiple Comparison Corrections....11
Tests for Brain Measures Mediating Significant CPF-Behavior Associations....11
DTI Brain Network Construction for Graph Theoretical Measures....11
Connectome (Graph Theoretical) Measures....12
rs-fMRI Graph Theory Measures....14

SUPPLEMENTAL DISCUSSION
Additional Relation of Findings to Animal Models....15
Somatotopy of the Internal Capsule....15
Additional Discussion of Inflammation and Oxidative Stress....15
Comment on Behavioral Findings....15

SUPPLEMENTAL REFERENCES....16

© 2025 Peterson BS et al. *JAMA Neurology*.

SUPPLEMENTAL TABLES........23
eTable 1: Correlation Matrix for Model Variables........24
eTable 2: Correlation Matrix for DTI Graph Theoretical Measures........25
eTable 3: Correlation Matrix for rs-fMRI Graph Theoretical Measures........27
eTable 4: Association of CPF Exposure with Neuropsychological Test Scores........28
eTable 5: Associations of CPF Exposure with Symptom Severity Scores........32
eTable 6: Association of CPF Exposure Levels with DTI and rs-fMRI Graph Theoretical Measures........33

SUPPLEMENTAL FIGURES........34
eFigure 1: Image Quality in Each MRI Modality........35
eFigure 2: Age Distributions by Participant Sex........36
eFigure 3: Statistical Maps of Prenatal CPF Exposure Associations with White Matter Surface........37
eFigure 4: CPF Associations with FA Values -- All Slices for Tissue-Specific White Matter Maps........38
eFigure 5: CPF Associations with ADC Values -- All Slices for Tissue-Specific White Matter Maps........39
eFigure 6: CPF Prenatal Exposure Effects on Axial and Radial Diffusivity in White Matter rCBF........40
eFigure 7: All Slices for CPF Exposure Associations with rCBF........41
eFigure 8: Statistical Map of Prenatal CPF Exposure Associations with NAA Levels........42
eFigure 9: All Slices for CPF Exposure Associations with NAA Levels........43
eFigure 10: Association of Prenatal CPF Levels with All Metabolite Concentrations........44
eFigure 11: Associations with Metabolite Concentrations Normalized to Creatine........45
eFigure 12: Scatterplot for FingerTapping Task........46
SENSITIVITY ANALYSES
Air Pollution: Covarying for Prenatal PAH and PM_{25} Exposure........47
eFigure 13: Anatomical Measures........48
eFigure 14: DTI and rCBF Measures........49
eFigure 15: MRS Metabolites........50
Covarying for Maternal Age at Birth........51
eFigure 16: Anatomical MRI........52
eFigure 17: DTI Measures........53
eFigure 18: rCBF........54
eFigure 19: Metabolite Concentrations........55
Replacing LOD with LOD/2 Values........56
eFigure 20: Anatomical MRI........57
eFigure 21: DTI Measures........58
eFigure 22: rCBF........59
eFigure 23: Metabolite Concentrations........60
CPF Variable Transformations........61
eFigure 24: Cortical Thickness and White Matter Measures........62
eFigure 25: DTI Measures........63
eFigure 26: rCBF........64
eFigure 27: Metabolite Concentrations........65

© 2025 Peterson BS et al. *JAMA Neurology*.

ONLINE-ONLY METHODS

Assessment of Maternal Characteristics

A skilled bilingual interviewer administered a questionnaire during the final trimester to collect demographic, residential, income, and education information, as well as health and environmental data. We also posed questions about methods used for pest control during pregnancy.[1] We used a common 4-item measure of material hardship[2] during the prenatal assessment to assess the mother's unmet basic needs in the areas of food, housing, utilities, and clothing).[2] Maternal characteristics were assessed during the prenatal period and at youth age 11-12 years, and their stability over this period was highly significant (material hardship: Pearson X^2=42.44, p<.001; income: r=.350, p<.001; maternal years of education: r=.636, p<.001).

When the children reached 3 years, we used the total score from the HOME[3,4] observational inventory as a measure of the overall physical (cleanliness, safety, healthfulness) and relational (parental responsiveness) quality of the home environment. The HOME variable was missing in 14 participants, and for them the variable was multiply imputed using a random hot deck procedure,[5] with results aggregated across the imputed datasets.

Geospatial Modeling-Based Estimates of Prenatal PM2.5 Exposure

We geocoded the residential addresses of each pregnant participant and then used spatiotemporal modeling to estimate average daily PM2.5 exposure throughout pregnancy, as previously described.[6] The model integrated air pollution data from two primary sources -- the New York City Community Air Survey (NYCCAS) and the Environmental Protection Agency's Air Quality System (AQS). NYCCAS provided data from 60-150 locations across New York City, converted from biweekly to daily averages. We also included daily data from regulatory monitors of the Department of Environmental Conservation, which collect data on an every-day or every-third-day schedule, to capture true daily air quality variation in our models. Detailed temporal patterns were further captured using data from regulatory monitors of the Environmental Protection Agency, which collect data on an every-day or every-third-day schedule. We computed the amount/density of candidate spatial predictors (such as traffic- and land use-related variables, derived from the New York City Department of City Planning's taxlot database) within 4 different buffer areas at 100, 300, 500 and 1000 meters around each monitor site. We also included several temporal predictors and computed daily city-wide averages, including temperature, wind speed, and relative humidity. We additionally calculated the air-mass trajectory and mixing depth for each day. We used an 80%-20% split to divide the data into training and test data sets and then employed 10-fold cross validation in model building on the training set, root mean square error (RMSE), and R^2 values to identify the strongest model. Gradient boosting machine models were the best fitting models for $PM_{2.5}$. The cross validation R^2 for $PM_{2.5}$ was 79% (RMSE 2.24 μg/m^3). When the model was applied to the test dataset, overall R^2 was 74% (RMSE 2.38 μg/m^3).

Personalized Measures of Prenatal PAH Exposure

Prenatal airborne PAH exposure was quantified through the summation of 8 specific nonvolatile PAH compounds: benzo[a]anthracene, chrysene/iso-chrysene, benzo[b]fluoranthene, benzo[k]fluoranthene, benzo[a]pyrene, indeno[1,2,3-c,d]pyrene, dibenzo[a,h]anthracene, and benzo[g,h,i]perylene. These were measured using personal air monitors worn by the mothers for a 48-hour duration in their third trimester [7]. The devices collected vapors and particles ≥2.5 µg in diameter on both a pre-cleaned quartz microfiber filter and a backup polyurethane foam cartridge. The Southwest Research Institute in San Antonio, TX, analyzed the samples for concentrations of the 8 PAH compounds. Each personal monitoring device's flow rate, time, and documentation completeness were evaluated to ensure accuracy, which confirmed that all samples were of satisfactory quality.

Measures from 48-hour personal air monitoring for prenatal exposure to these 8 PAH compounds[8] were validated against 2-week residential air sample monitoring in a representative subset of homes (n=101) over 6 weeks during the last trimester.[9] The pollutants measured in the initial two weeks showed significant correlation with those measured in the subsequent four weeks (r=0.57-0.76, p<0.001). Additionally, the 6-week indoor air pollutant levels significantly correlated with the levels in the single 48-hour prenatal personal air sample (r=0.44, p<0.001). Concurrent indoor, outdoor, and personal monitoring of PAH in a separate study of pregnant women also showed a high inter-correlation (pairwise Spearman's coefficients for 9 PAHs ≥ 0.84, p < 0.01),[10] indicating that our single PAH measure in prenatal personal air was a reliable indicator of chronic prenatal inhalation exposure.

Cotinine Levels These were measured to provide an index of maternal exposure to second-hand tobacco smoke. Maternal blood (30-35 ml) was collected in the hospital within 24 hours of delivery, and umbilical cord blood (30-60 ml) was collected at delivery. Samples were transported to the laboratory immediately, where buffy coat, packed red

© 2025 Peterson BS et al. *JAMA Neurology*.

blood cells, and plasma samples were separated and stored at -70°C. A portion of each sample was shipped to the Centers for Disease Control for analysis of plasma cotinine using high-performance liquid chromatography atmospheric-pressure ionization tandem mass spectrometry.[11] The maternal and cord plasma concentrations of cotinine were significantly intercorrelated ($r=0.88$, $p<0.001$), and maternal values were substituted for missing cord cotinine values.

Exposure to Second-Hand Tobacco Smoke Mothers reported whether anyone in the household smoked tobacco products during the pregnancy. Responses were coded dichotomously as yes or no. Cord cotinine levels at birth correlated significantly with the report of second-hand smoke exposure prenatally in the household ($r=0.52$, $p<10^{23}$), validating the maternal report.

Youth Neuropsychological Tests

We assessed cognitive performance in domains previously implicated in prior preclinical and clinical studies of CPF exposure, including attention, memory, inhibitory control, and sensorimotor functioning. Tests were administered over a single 3-hour session within 6 weeks of the MRI scan by a research assistant who was trained and supervised by a developmental neuropsychologist. Breaks were provided to minimize fatigue.

We administered: the *NEPSY-II*[12] subtests for Fingertip Tapping (sensorimotor, motor programming), Design Copy (visuomotor), Geometric Puzzles (visuospatial), Visuomotor Precision (psychomotor fluency), Auditory Attention (attention), Response Set (cognitive flexibility), Inhibition (inhibition, cognitive flexibility), Clocks (visual reasoning); *Purdue Pegboard Task*[13] (fine and gross motor dexterity and coordination); *Conners Continuous Performance Test-II (CPT-II)*[14] (inattention, impulsivity, discrimination of signal from noise); *Wechsler Intelligence Scale for Children-IV*[15] (general intelligence).

Youth Mental Health Symptom Ratings

Parents completed the Child Behavior Checklist[16] to provide measures of mood, ADHD, thought disorder, oppositional, and conduct disorder symptoms. Mood disturbances were further assessed with the Children's Depression Rating Scale,[17] anxiety symptoms with Revised Children's Manifest Anxiety Scale,[18] ADHD symptoms using the ADHD Rating Scale,[19] and social processing with the Social Responsiveness Scale.[20]

MRI Scanning

MRI Scanning was acquired with a 3 Tesla GE Signa HDx system (Milwaukee, Wisconsin) equipped with an 8-channel receive-only head coil. A 3-plane localizer was used to ensure subsequent images were aligned with the anterior–posterior commissure line. We dedicated extra time on the morning of the scan to help participants acclimate to the MRI environment and its noise. Techniques such as gentle reminders, praise, and relaxation exercises were employed to ease anxious children, facilitating the capture of images without motion artifacts. The entire scanning process required approximately 75 minutes, including breaks for participants to rest, move around, and to repeat sequences if motion artifacts were identified.

Throughout the MRI scan acquisition, we meticulously monitored the data for each sequence in real-time as images materialized and were displayed on the scanner console. Any instances of visible motion prompted an immediate reacquisition of the affected sequence. Within 48 hours post-scan, we conducted a detailed assessment of image quality across individual pulse sequences as part of our preprocessing regimen. We strove to reschedule participants for repeat scans using the pertinent pulse sequences whenever their images exhibited motion artifacts.

MRI Pulse Sequences

Fewer DTI and ASL datasets were acquired (202 and 175, respectively) than anatomical (262) and MRS (211), for several reasons. We prioritized the anatomical sequence by positioning it first in the acquisition series, so it was most often acquired successfully. DTI was positioned last because the reconstruction time was prohibitively long, which would have increased the total time for participants to be in the scanner had it been placed earlier. Because DTI was the last sequence, it was more often unable to be acquired, either for technical or practical reasons (e.g., the limit of the scheduled scan time was reached). The ASL sequence became available after the study was underway, and so it was unable to be acquired in participants who were scanned earlier in the study.

Anatomical MRI We acquired high-resolution T1-weighted (T1w) images using a 3D Fast Spoiled Gradient Recall (FSPGR) sequence with sagittal reconstruction. The parameters were as follows: repetition time (TR)=2170 msec, echo time (TE)=1.3 msec, inversion time=500 msec, flip angle=11°, matrix=256x256, field of view=25 cm, phase field of view=100%, slice thickness=1.0 mm, acceleration factor=2, with 160 slices and a voxel size of $1x1x1mm^3$. We acquired two images at NEX=1, which were then averaged offline.

© 2025 Peterson BS et al. *JAMA Neurology*.

Diffusion Tensor Imaging (DTI) DTI data were acquired in oblique slices aligned to the AC-PC line using single-shot echo-planar imaging sequences, with specifications including a matrix=132x128 zero-padded to 256x256, TR=8000 ms, TE= ~74 ms, FOV=24 cm, Flip=900, Slices=60, Slice thickness=2.5 mm, Slice Spacing=0 mm; NEX=2, PFOV=1.0. We acquired 3 baseline images at b=0 s/mm^2 and 15 diffusion-weighted images (DWIs) at b=1000 s/mm^2 in uniformly distributed directions, applying phase correction and an ASSET acceleration factor of 2.
Multiplanar Chemical Shift Imaging (MPCSI) data were gathered in 6 axial oblique slices aligned with the AC-PC line, ensuring the second bottom-most slice captured the AC-PC plane. The sequence settings were TE=144 ms, TR=2800 ms, field of view=24 cm, slice thickness=10.0 mm, slice spacing=2.0, number of phase encoding steps=24x24, NEX=1, spectral width=2000 Hz, with 512 complex data points. Water signals were minimized using the CHESS sequence, and lipid signals from outside the brain were reduced with 8 angulated saturation bands. MPCSI images were aligned with a template brain using a localizer image of high in-plane resolution, acquired with the same orientation and slice positions as the MPCSI data, with parameters including TR=300 ms, TE=10 ms, FOV=24 cm, slice thickness=10.0 mm, spacing=2.0 mm, acquisition matrix=256x128, image zero-padded to 256x256.
Arterial Spin Labeling (ASL) Our Pulsed Arterial Spin Labeling (PASL) perfusion sequence was optimized for parallel imaging at 3T with a PICORE (Proximal Inversion with Control for Off-Resonance Effects) QUIPSS II sequence.[21] A 9-cm tagging slab was positioned 16-mm below the proximal edge of the imaging volume. Control images were captured using off-resonance adiabatic hyperbolic secant RF pulses matching the frequency offset of the labeled images, without the slice-selective gradient, to manage off-resonance effects. Image acquisition utilized a single-shot, gradient-echo, echo planar imaging (EPI) sequence, with parameters of FOV 24 cm, 64x64 matrix, TE/TR=24/2300ms, flip angle 90°, slice thickness 6 mm, inter-slice spacing 0.5 mm, achieving a spatial resolution of 3.75x3.75x6.5 mm. We collected 18 slices from inferior to superior sequentially. Each ASL scan, including 151 acquisitions plus 5 dummy images, lasted 5 min 59 sec. Additionally, an M0 scan using gradient-echo EPI with a TR of 15sec was acquired at the same resolution and slice position as the ASL data. The M0wm from white matter was measured and utilized in the offline calculation of rCBF.[21] Furthermore, a T1-weighted localizer image with high in-plane resolution, matching the slice locations of the ASL data, was acquired for aligning each participant's ASL data with their T1-weighted anatomical MRI image. The localizer image was captured using a 2D, fast spin echo pulse sequence with an echo train length=9, TR=2150ms, TE=9.94ms, TI=840ms, flip angle=90°, in-plane resolution=0.94x0.94 mm^2, slice thickness=6.5mm, with a total acquisition time of 1 min, 46 sec.
Resting-State fMRI (rs-fMRI) We employed a multi-slice, single-shot, echoplanar imaging sequence in the axial orientation, with slices oriented parallel to the AC-PC plane, with TE = 30 ms, flip angle= 90º, TR = 2200 ms, slice thickness = 3.5 mm, slice gap = 0 mm, 34 slices, FOV = 24 x 24 cm^2, matrix size = 64 x 64, two runs each with 128 dynamic volumes and 6 dummy volumes. During image acquisition participants were instructed to remain still with their eyes closed and to let their minds wander freely. Total scan time: 9 min 50 sec.
Scans Attempted and Usable In all 270 participants we acquired usable MRI scans, of which 264 were anatomical(270 attempted) 204 DTI(244 attempted), 176 ASL(210 attempted), 213 MRSI(235 attempted), and 168 rs-fMRI (233 attempted, with 14 excluded for quality and 41 for not having all ROIs required for connectivity analyses. Differing numbers of sequences attempted was determined primarily by participant tolerance to stay in the scanner for the time needed to acquire all sequences, and the ASL pulse sequence became available after scanning had begun).

Image Processing Methods

Anatomical Image Processing

Quality Control We evaluated the sharpness, ringing, and contrast-to-noise ratio between subcortical nuclei and the gray/white matter interface. Of 270 anatomical scans attempted, 7 were excluded because of quality, leaving 264 for analyses.
Preprocessing Morphometric analyses were performed using ANALYZE 7.5 software (Biomedical Imaging Resource, Mayo Foundation, Rochester, Minnesota), with our operators remaining unaware of infant characteristics. We first removed large-scale intensity variations in the images[22] and then aligned them to a standard orientation using midline landmarks to correct for any head rotation and tilt. To ensure unbiased segmentation, images were randomly flipped left-right before segmentation, then reverted to their original orientation after segmentation but before spatial coregistration. Connecting dura was meticulously removed manually in the transverse view and verified in orthogonal views. The brain was divided into hemispheres using a curvilinear plane positioned through standard midline landmarks. The cerebellum was removed where the peduncles join the brainstem, the brainstem was transected at the pontomedullary junction, and the brain was split into two hemispheres.

© 2025 Peterson BS et al. *JAMA Neurology*.

The high resolution T1-weighted anatomical images were segmented in native imaging space into gray and white matter. Brain was isolated from non-brain tissue using an automated brain extraction tool (BrainSuite, RRID:SCR_006623)[23] followed by manual editing in the axial, coronal, and sagittal views using an in-house generated program to remove any dura that remained connected to the brain. We used a semi-automated method to segment brain tissue as gray or white matter, with an expert neuroanatomist sampling gray-scale values of both cortical gray matter and white matter at 4 standard locations throughout the brain. The sampled values were averaged to generate mean values for gray and white matter; these values were used to threshold and generate initial tissue segmentations, which were then edited to remove the subcortical gray matter. The test-retest intraclass correlation coefficient (ICC)[24] for the cortex was >0.98.

Cortical Thickness Measurement We spatially normalized all brains to a template brain and then applied to each coregistered brain a 3D morphological operator to distance-transform each brain without its cortical mantle to the surface of the cerebrum.[25] We calculated cortical thickness as the smallest distance of each point on the cerebral surface to the outermost surface of the white matter. Cortical thickness was calculated with brains scaled to the template, thereby accounting for overall scaling effects on thickness measures.

Deformation-Based Measures of Brain Morphology

Overview We used previously validated tools for deformation-based morphometry, developed in-house, to perform fine-grained analyses of localized morphological features across the cerebral surface.[26] We first spatially coregistered each brain to a template brain to identify corresponding points across the surfaces of each brain and the template, and then calculated the distance of each point on the surface of each brain to the corresponding point on the surface of a template brain.[27] These distances were encoded as positive for outward and negative for inward deformations relative to the template. The distances formed a set of continuous variables at each point on the surface of the template brain that were then subjected to statistical modeling. These distances were calculated separately for each point on the surface of the cerebrum and for each point on the surface of white matter.

Morphological effects in statistical analyses of these distances at a given location represent, strictly speaking, distances of the surfaces of participant brains from the corresponding location on the template brain. These distances in turn reflect shape features of the surface – outward and inward deformations (protrusions and indentations) relative to the surface of the template. For the sake of simplicity and brevity, we refer to these effects using less precise but more intuitive terminology, as increases or decreases in *"local volumes"*.

Spatial Co-Registration We first brought each participant brain into the same overall volume and into close spatial approximation of the template by treating the brains as rigid bodies, applying to them a similarity transformation (consisting of 7 parameters -- 3 translations, 3 rotations, and 1 global scaling) that maximized the mutual information in the anatomical images' gray scale values between each brain and the template.[28]

Identification of Point Correspondences Across Brain Surfaces Next we treated each participant brain as a viscous fluid that molded its surface into the identical size and shape of the template: each brain was nonlinearly transformed to the template using a high-dimensional, non-rigid warping algorithm based on fluid dynamics.[29] The identical surfaces allowed us to label each point on each surface, establishing point correspondences across each brain surface with the template. The nonlinear deformation was then reversed, retaining the point-to-point correspondence across the surfaces of all spatially coregisered brains in the sample.

Calculation of Distances Used in Statistical Modeling We next calculated the Euclidean distance of each point on the surface of each spatially coregistered brain from the corresponding point on the template brain.

Selection of the Template Brain We used a single representative brain for the template, rather than one derived by averaging brains across multiple participants, because a single brain has well-defined tissue interfaces, including CSF-gray matter or gray-white matter interfaces, that improve the precision of spatial co-registration and the identification of corresponding points across brains. Moreover, gyri and sulci on the cerebral surface differ markedly across individuals,[30] and not all individuals have every gyrus or sulcus, complicating the generation of a synthetic, average cerebral surface.

We employed a rigorous, 2-step procedure to select a template brain to ensure that findings were not unduly influenced by selection of a non-representative template. First, we identified, as a preliminary template, the brain of one participant whose age and overall brain size were nearest the group averages. The brains for all remaining participants in the sample were coregistered to that preliminary template, the point correspondences across their surfaces were determined (described above), and then the distances of those points from the corresponding points on the preliminary template surface were calculated. The brain for which all points across its surface were closest (in the least squares sense) to the average of the distances across those points for the entire sample was selected as the final template, thereby yielding a template brain that is specific to and morphologically most representative of all brains in this cohort. The use of a morphologically representative brain minimizes registration errors and yields the most accurate point correspondences across brain surfaces. Additionally, we visually assessed the accuracy of each

© 2025 Peterson BS et al. *JAMA Neurology*.

step in coregistration and the point correspondences with the template, adjusting coregistration parameters manually by an expert if indicated. The same brain was used as the template across all MRI modalities. Despite the care taken in selection of the most representative template brain, we also note that our findings are robust with respect to the specific template used, as the findings differed minimally when using any randomly selected brain from our sample as the template. Finally, for more accurate anatomical localization, we mapped the anatomical findings onto the freely available Colin27 brain[31] with enhanced sulcal definitions (https://www.bic.mni.mcgill.ca/ServicesAtlases/Colin27). The mapping included first an affine (3 translations, 3 rotations, and 3 scaling parameters) coregistration of the Colin27 brain to our template brain. Subsequently, findings on the template brain were transferred onto the coregistered Colin27 brain using a 2nd nearest neighbor mapping.
Consideration of Alternative Deformation-Based Metrics Our anatomical measures are not strictly "volumes", but instead are distances of from locations on participant brains to the corresponding location on the template brain. We considered alternative measures commonly used in deformation-based analyses of brain morphology, such as the Jacobian of the 3D warping matrix. The Jacobian, however, is a nonlinear transform: it decreases from 1 to 0 as the local volume decreases to a point, but it increases from 1 to an unbounded value as the local volume increases. These properties undermine intuitive biological interpretation of the Jacobian. For example, a change in Jacobian from 1 to 1.5 represents a change in volume that differs from a change in the Jacobian from 1.5 to 2.0. Thus, the nonlinear changes in Jacobians provide findings that are exceedingly difficult to interpret in biological terms. Our surface distance measures, in contrast, represent local indentations or protrusions of the cerebral surface, which are easily understood and that can be intuitively interpreted as local volume effects. Moreover, our distance measures are generally normally distributed, whereas distribution for the Jacobian are usually highly skewed,[32] which is undesirable for the dependent variable in parametric statistical analyses.

DTI Processing

Quality Control We implemented quality control steps to filter out datasets marred by excessive motion. Each exam underwent a rigid body transformation to align all diffusion-weighted images (DWIs) with their B0 image, utilizing the derived 3 translation and 3 rotation parameters to calculate two summary statistics for head motion assessment: (1) the Root Mean Squared (RMS),[33] estimating the root of the mean squared displacement for a head modeled as a 50 mm radius sphere; and (2) Mean Framewise Displacement (FD),[34] measuring head movement by translating rotational angular displacements to translational displacements on the surface of a 50 mm radius sphere. DWIs with motion estimates exceeding 0.5 mm by either RMS or FD were excluded; exams with more than 10% of images meeting this criterion were removed from subsequent processing steps. Spatial distortions caused by eddy currents along the anterior-posterior axis were corrected using quadratic warping.[35]

Rapid changes in magnetic susceptibility from brain tissue to the nasal cavities create B_0-field inhomogeneities that cause spatial distortions of the MRI signal in inferior frontal brain regions. Spatial distortions depend upon the frequency encoding direction and visually either stretch or indent the inferior frontal gyri. These spatial distortions can be modeled and accounted for if the reference MR images have been acquired with frequency encoding applied in both the anterior-to-posterior and posterior-to-anterior directions. Top-up procedures use these reference scans to correct for the spatial distortions that B_0-field inhomogeneities create. We did not acquire these reference scans, however, and therefore could not account for these spatial distortions. Nevertheless, these distortions are largely limited to gray matter of the inferior frontal gyrus, which we delimited manually in the template coordinate space and then masked out in our statistical analyses. Finally, we assessed motion visually by constructing tensor color maps from the retained images and displaying the principal eigenvectors throughout the brain, which show a color bias in the presence of motion artifact. Of 244 DTI scans attempted, 40 were excluded for quality, leaving 204 for analyses.
Maps of DTI Scalar Indices In DSI Studio (RRID:SCR_009557), we estimated the diffusion tensor (D) at each voxel of the pre-processed DTI data.[36-38] We ensured that the fitted tensor D was positive definite by first decomposing the tensor as the product $D = A{*}A^T$ and estimating the matrix A, then using the estimated matrix $\tilde{A}$ to compute the positive definite tensor $\tilde{D} = \tilde{A} * \tilde{A}^T$. We decomposed this positive definite tensor into its eigenvalues $(\lambda_1, \lambda_2, \lambda_3)$ and eigenvectors (v_1, v_2, v_3), which we then used to compute the scalar indices fractional anisotropy (FA), average diffusivity coefficient (ADC), axial diffusivity (AD), and radial diffusivity (RD):

$$FA = \frac{\sqrt{(\lambda_1-\lambda_2)^2+(\lambda_2-\lambda_3)^2+(\lambda_3-\lambda_1)^2}}{\sqrt{2*(\lambda_1^2+\lambda_2^2+\lambda_3^2)}}, ADC = \frac{1}{3}(\lambda_1+\lambda_2+\lambda_3), AD = \lambda_1. \text{ and } RD = \frac{1}{2}(\lambda_2+\lambda_3)$$

FA indexes the degree of directional diffusion of water and is a marker for local organization and integrity of white matter fibers.[39] ADC represents the directionless magnitude of water diffusion. AD and RD measure the magnitude of water diffusion in the direction parallel and perpendicular, respectively, to the primary axis of the diffusion

© 2025 Peterson BS et al. *JAMA Neurology*.

tensor. When considered together, the maps of FA, ADC, RD, and AD aid interpretation of the biological basis for DTI findings.

Spatial Normalization of DTI Maps Next, we edited the ADC maps to remove nonbrain tissue, then thresholded the edited map to remove background noise. We used this edited ADC map to mask out nonbrain tissue in the FA, AD, and RD maps. The edited maps for each participant were coregistered using a rigid body transformation (3 translations and 3 rotations) to the participant's T1-weighted (T1w) anatomical image. The participant's T1w image was in turn normalized to the template T1w image as described above for anatomical image co-registration. Finally, we applied the same affine transformation and nonlinear warping to the coregistered FA, AD, ADC, and RA maps to spatially normalize those maps into the coordinate space of the template brain. The maps of FA, AD, ADC, and RA were subsequently smoothed using a Gaussian kernel with FWHM = 4 mm.

Tissue-Specific Maps We generated tissue-specific maps for the DTI scalar indices to minimize partial volume effects that can be a consequence of subtle morphological differences across brains when normalizing DTI data into the template space, particularly at the interface of differing tissue types. These tissue-specific maps provided greater confidence in locating statistical effects as being in either gray or white matter of the brain. We used FA values to threshold gray and white matter. FA values, however, will be lower in regions of crossing fiber tracts or sharply curving fiber trajectories, and therefore use of a fixed FA threshold value could incorrectly classify some white matter voxels as gray matter and vice versa. To minimize the effects of these misclassifications, we defined gray matter as voxels with an FA lower <0.35 and white matter as voxels with an FA >0.40. Though this use of a non-overlapping range for GM and WM suppressed some findings, it enhanced the validity of tissue classification at the interface of gray and white matter and in regions of crossing fibers or sharply curving fiber trajectories. Finally, we used tissue-specific FA maps to segment tissue-specific maps for AD, RD, and ADC in the same brain.

Because some voxels in the tissue-specific maps for gray and white matter have scalar indices from fewer than all study participants (due to excluding data at voxels with FA values between the bounds defining gray and white matter), we present statistical findings at only voxels that had data from at least 60% of all participants.

MPCSI Processing

Quality Control We assessed MRS data quality by reconstructing the data, assessing it for excess noise, and then examining the spectrum in each voxel for baseline distortions, signal contamination by lipid signal from the scalp, incorrect placement of suppression bands, or broadening of line width. Datasets exhibiting any of these problems were excluded from further processing. Of 235 MPSI datasets attempted, 22 were excluded for quality, leaving 213 for further analyses.

Signal Processing We processed the signal from each coil of the 8-channel head coil separately before combining their processed MRS signals to generate the spectroscopic images.[40] First we phase-aligned signals, then smoothed the aligned signals using a Hamming window filter, spatially reconstructed the time-domain free induction decay (FID) signal in each slice with a 2D Fourier transform, suppressed residual water signal by applying a high pass filter to the FID signal, performed line broadening using a 4 Hz Gaussian filter, and then transferred the time-domain signal into the frequency domain using a 1D Fourier transform.[41] Finally, the processed frequency-domain signals from each of the 8 coils were combined by computing their weighted sum (MATLAB, RRID:SCR_01622). The combined signal was then loaded into the software *3DiCSI* (3D Interactive Chemical Shift Imaging, RRID:SCR_002581) to identify MRS voxels within the brain and save spectral data for those voxels. Spectral fitting was applied to the frequency-domain signal by identifying peaks for N-acetylaspartate (NAA), creatine (Cr), choline (Ch), glutamate + glutamine (Glx), and lipids, with a modeling spectrum for those peaks of Gaussian-Lorentzian curves. The areas under the fitted curves provided metabolite concentration estimates for each voxel of the brain.

Normalization of Metabolite Levels Because water signal was suppressed when acquiring MPCSI data, we were unable to calculate metabolite ratios using water as the reference. For several important reasons, we were reluctant for our primary analyses of MRS data to follow the common practice of using creatine levels as the reference for other metabolites (i.e., calculating their ratios to creatine). First, ratios tend to amplify noise in both the numerator and denominator, making them generally unsuitable for statistical analysis. Second, the use of ratios can lead to misleading conclusions in developmental research, since both components of the ratio may change with age. Therefore, to interpret the age-related significance of a ratio, one must separately evaluate the impact of both the numerator and denominator to ascertain which metabolite is influencing the observations. Third, exposure to CPF could potentially affect creatine levels, thereby confounding analyses of metabolite-to-creatine ratios.

Therefore, our primary analyses of MRS data instead normalized metabolite concentrations to noise levels, thereby obviating problems with normalizing to creatine and afford clearer insight into the pathophysiological significance of brain metabolite concentrations. Influences on noise in the MRI signal, and consequently in its SNR (signal-to-noise ratio), include: the magnetic field strength (B_0); the coil's quality factor Q, representing the energy

© 2025 Peterson BS et al. *JAMA Neurology*.

an RF coil stores compared the energy it dissipates per cycle; the geometry factor g, pertaining to the coil's physical design, the imaging plane's location, and the field of view; and the filling factor η, indicating the energy stored in the head relative to total energy in the RF coil.[42] Additional influences include potential shifts in the static magnetic field over time or following updates to software or hardware,[43] which could significantly alter both signal and noise levels in collected data. Although variations in the filling factor could influence SNR, the comparable ages of our participants and the consistent use of an 8-channel head coil ensured a relatively stable filling factor across participants. Thus, the primary factor affecting signal variation was likely was a gradual shift in both signal and noise equally over the years of data collection. We therefore accounted for this gradual shift in our analysis by calculating the ratio of metabolite peak to background noise for each voxel and using this ratio in our primary statistical analyses. We also conducted sensitivity analyses, however, in which we used the more conventional ratios of metabolite concentrations to creatine (NAA/Cr, Cho/Cr, and Glx/Cr) in assessing associations with prenatal CPF exposure levels.

Background noise for the MRS spectrum was computed as the standard deviation of the real part of the complex spectrum free from the metabolite signal. We then calculated the signal-to-noise ratio (SNR) for each metabolite concentration. The average SNR for the NAA metabolite was greater than 280, which is an excellent SNR attributable to use of the 8-channel head coil. A spectroscopic image (SI) for each metabolite was generated next as the ratio of the peak area to the background noise for each MRS voxel within the brain.

Correcting Partial Volume Effects The spectroscopic images were also processed to correct for both partial volume effects within each MRS voxel and the spread of MRS signal of each voxel to its neighboring voxels (i.e., the point spread function, PSF). The spread of MRS signal from one voxel to its neighbors derives from the use of a small number of k-space samples when acquiring the spectroscopic data and from smoothing the data with a Hamming window prior to spatial reconstruction. We estimated the PSF by simulating the acquisition of MRS data within k-space on a 24x24 grid and then using a Hamming window to spatially filter the simulated data. The resulting 24x24 complex array was interpolated to 256x256 to match the spatial resolution of the T1-weighted MR images.

Partial volume effects reference the fact that an MRS voxel may contain more than one tissue type – i.e., gray matter (GM) and white matter (WM) -- in varying proportions; therefore, the signal in that voxel is a proportionate combination of signals from each of those tissues. To estimate the proportions of GM and WM within an MRS voxel, brain tissue was segmented as either GM or WM, as described above, within each participant's high-resolution (1mm^3 voxel) T1-weighted image and then coregistered to the MRS data of that participant (detailed below). We then convolved the coregistered tissue definitions with the PSF function and calculated the fractions of GM and WM within each MRS voxel. We next used a linear regression model[44] at each voxel i and for each metabolite j, along with the concentrations S_{ij} of that metabolite in the neighboring voxels, to estimate the concentration of that metabolite within gray matter M_{ij}^{G} and white matter M_{ij}^{W}:

$$S_{ij} = \left| c_i^G * M_{ij}^G + c_i^W * M_{ij}^W \right| + n$$

where c_i^G and c_i^W are the proportions of GM and WM, respectively, at voxel i and n is noise. We then tri-linearly resampled the metabolite concentrations M_{ij}^{G} and M_{ij}^{W} from low resolution MRS data to high resolution anatomical data for spatial normalization across study participants. e**Figure 1** shows a representative spectrum in a voxel of the MPCSI dataset.

Spatial Normalization of MPCSI Data MPCSI data for each participant were coregistered into the coordinate space of a T1-weighted image of a template brain. Each participant's localizer image was coregistered to its high-resolution T1w image using a similarity transformation such that the transformation maximized mutual information[45] across the localizer and its corresponding high resolution T1w image. Second, we spatially transformed the localizer image using the similarity transformation that coregistered the T1w image of the participant into the coordinate space of the template brain. Third, we warped the coregistered localizer by applying to it the high-dimension, nonlinear deformation that warped the participant's T1w image to the T1w template image. We applied these 3 coregistration procedures to each of the metabolite images.

Effects of Saturation Bands The MPCSI saturation bands applied to suppress lipid signal from the scalp were not as precisely shaped as the scalp, and they unavoidably suppressed metabolite signals from several portions of cortical gray matter. Moreover, lipid signal from the small portions of scalp that were unsuppressed contaminated MRS signal to some degree within the brain, and those voxels were censored from further analyses during the detailed visual inspection of the spectra recorded from each of the 8 channels of the multi-channel head coil. Consequently, metabolite measures for many participants were available only in voxels of WM and deep gray matter nuclei. The metabolite images were subsequently smoothed using a Gaussian kernel with FWHM= 4mm. We show in **eFigure 1** a gray scale image representing the number of participants who had usable data at each voxel. We suppressed display of results at any voxel that did not have usable data from at least 75% the participants.

© 2025 Peterson BS et al. *JAMA Neurology*.

Processing of Arterial Spin Labeling Data

ASL provides absolute and reproducible measures of rCBF[46,47] by magnetically labeling water in arterial blood as a diffusible tracer, analogous to the use of ^{15}O water in PET scanning.[48] Unlike PET, ASL does not require the use of a radioactive tracer and therefore can be used in children, and it is a less costly and procedurally less complicated set of procedures. Moreover, because perfusion signals are obtained by pair-wise subtraction of adjacently acquired tagged and control images, ASL is less prone to motion artifact, low frequency physiological noise, and baseline drift than more conventional BOLD-based functional imaging.[49]

We aligned the PASL brain images and the M_{0_WM} image to the first PASL image for each participant in native imaging space to correct for head motion. We spatially smoothed the coregistered PASL images using a Gaussian kernel of 6mm FWHM (Full Width at Half Maximum) to improve signal-to-noise ratio while avoiding loss of spatial precision in locating our effects of interest. We generated a brain mask for each participant based on the mean PASL image. We constructed for each participant a voxel-wise map of rCBF from the PASL time series and M_{0_WM} image using in-house software: (1) We pair-wise subtracted the control images from the labeled images; (2) From the average of the subtracted images, we calculated rCBF at each voxel as $rCBF = \frac{6000*\Delta I}{2\alpha*M_{0\_B}*TI_1*exp(-TI_2/T_{1\_B})}$, where ΔI is the image difference obtained in step 1; α is the tagging efficiency, for which we used the default value of 0.9; $TI_1 = 600$ ms is the time to QUIPSS saturation; $TI_2 = 1300$ ms is the inversion time of the first slice and is slice time corrected for the rest of the slices in the imaging volume; and T_{1B} is the T_1 of blood, for which we used the default value of 1664 ms;[50] M_{0_B} is the MR signal from a voxel filled with arterial blood, estimated from the M_{0_WM} map as $M_{0\_B} = rM_{0\_WM}e^{(1/T_{2WM}-1/T_{2B})TE}$ where *r* is the proton density ratio of blood, for which we used a default value of 1.06; and where the default values for T_{2WM} and T_{2B} were 70 ms and 200 ms, respectively.[21,51,52] We used a 6 degrees-of-freedom rigid-body transformation such that mutual information[45] is increased to coregister the anatomical images to the localizer images for each participant. Subsequently, each participant's anatomical image is used as an intermediary source to coregister the rCBF images to a template brain by applying a similarity[45] followed with a nonlinear transformation based on fluid flow.[53] We selected a single individual brain as the template brain, which was morphologically the most representative of participant brains.[54] Finally, the perfusion maps normalized into the template space were smoothed using a Guassian kernel with FWHM = 2mm.

Quality Control We quantified the amount of head motion in the ASL data of each participant using two summary statistics, Root Mean Squared (RMS)[33] and Mean Frame-wise Displacement (FD) metric sums differentiated realignment estimates,[55] derived from three translational (x,y,z) and three angular rotational (roll, pitch, yaw) measures. These two motion indices were highly intercorrelated (r=0.99). Of 210 ASL datasets attempted, 34 were excluded for quality, leaving 176 for further analyses.

Resting-State fMRI Processing

We used CONN functional connectivity toolbox[56] within SPM-12 to process the rs-fMRI data. fMRI data were coregistered and resampled using b-spline interpolation to the first imaging volume as the reference. This procedure also corrected susceptibility distortion and distortion-by-motion interactions by resampling the functional data to match the deformation field of the reference, as previously described.[57] Slice times were corrected using Sinc interpolation to the middle slice of the volume.[58] Images were spatially normalized into standard MNI space using SPM's algorithm for iterative segmentation and nonlinear spatial transformation.[59] Images were then resampled 2 mm^3 isotropic voxels using a 4th order spline and spatially smoothed using a Gaussian kernel with full width half maximum (FWHM) = 6mm.

Quality Control Framewise Displacement (FD) was calculated as the largest displacement of the six control points placed at the center of a 140x180x115mm bounding box that enclosed the brain. Volumes with FD > 0.9mm or global BOLD signal > 5 standard deviations from the mean were flagged as outliers. We excluded exams that had >5% of their volumes flagged as outliers. Of the 223 rs-fMRI exams acquired, we excluded 14 exams with >5% outliers and another 41 exams that did not have fMRI data in all regions of interest (ROIs) required for connectivity analyses, leaving 168 usable exams for rs-fMRI connectivity analyses.

SUPPLEMENTAL STATISTICAL ANALYSES

Sensitivity Analysis Using the Inverse Hyperbolic Sine Transformation

© 2025 Peterson BS et al. *JAMA Neurology*.

When including the two participants with CPF outlier values, we employed an inverse hyperbolic sine (IHS) transformation to minimize the influence of those outliers[60,61] ($\mathrm{IHS(x)} = \log\left[x + \sqrt{x^2+1}\right]$), in which the transformed value approaches the log of twice the exposure for large values and is zero for no exposure.

Statistical Parametric Mapping and Multiple Comparison Corrections

We used the parametric, cluster size-based familywise error rate (FWER)[62,63] to control for Type I errors when testing multiple hypotheses all voxels across the brain within each statistical map. We first conducted statistical analyses at each voxel to generate a statistical parametric map or a random field $X(t)$ of t statistic across the brain. The statistical map was thresholded at a cluster defining threshold (CDT) of 2.0 to identify clusters of findings where the random field had values greater than the CDT. We then modeled the number of clusters m as a Poisson distribution,[62,63] i.e. $P(m = k) = \frac{1}{k!} \cdot \lambda^k \cdot e^{-\lambda}$, where λ was computed as the expected Euler characteristic[64-68] $\mathbf{E}(\chi_u) = L(S) \cdot (2\pi)^{-2} \cdot |A|^{1/2} \cdot (u^2 - 1) \cdot e^{-u^2/2}$ at CDT = u, $L(S)$ was the Lebesgue measure of the volume S, and A was the determinant of the covariance matrix for the first order partial derivatives of the random field $X(t)$. The distribution $P(n = k)$ of the cluster size n was modeled[62,69] as $P(n = k) = \frac{2\beta}{3} \cdot k^{\left(\frac{2}{3}-1\right)} \cdot \exp\left(-\beta k^{2/D}\right)$, where $\beta = \left[\Gamma\left(\frac{3}{2}+1\right) \cdot \mathbf{E}(\chi_u)/\mathbf{E}(N)\right]^{2/D}$, and N was the number of locations with values greater than the threshold u. For cluster-level inference that controls for FWER, we then computed the probability of one or more clusters having size greater than k; that is, $P(n_{\max} \geq k) = \sum_{i=1}^{\infty}\left[p(m = i) \cdot [1 - P(n < k)^i]\right] = 1 - \exp\left[-\mathbf{E}(\chi_u) \cdot \exp\left(-\beta \cdot k^{\left(\frac{2}{3}\right)}\right)\right] \approx \mathbf{E}(\chi_u) \cdot P(n \geq k)$. The clusters with $P(n_{max} \geq k) < 0.05$ were considered statistically significant.

Tests for Brain Measures Mediating Significant CPF-Behavior Associations

We used the Sobel test[70,71] to assess whether brain measures significantly mediated the association of CPF exposure levels with cognitive and symptom measures obtained at the time of MRI scan. Let X be exposure, Y be the behavioral score, and M be the brain measure. We assessed mediation at each voxel using 3 regression equations: 1) $Y = c_1X + e_1$, assessing the association of CPF exposure with behavioral score; 2) $M = aX + e_2$, assessing the association of CPF exposure with the putative brain mediator; and 3) $Y = c_2X + bM + e_3$, associating the association of CPF exposure with behavioral score when adjusting for the mediator (termed the "direct effect" of X with Y). Covariates in all three equations included age at the time of scan, sex, ethnicity, maternal education, material hardship during pregnancy, and home environmental stress. We then tested whether the indirect effect $a\ x\ b$ differed significantly from zero using a z-score, $z_{ab} = (a\ x\ b)/se_{ab}$, where $se_{ab} = \sqrt{(a^2\ x\ se_b^2) + (b^2\ x\ se_a^2)}$, and se_a, se_b were the standard errors of the regression coefficients a and b, respectively.[72] A significant mediating effect would suggest that association of X with Y in regression (1) is weakened by M in regression (3).

DTI Brain Network Construction for Graph Theoretical Measures

Overview A graph, in mathematical terms, represents a network delineating the relationships between its constituent elements. This network comprises nodes or vertices interconnected via edges. For DTI data, these edges are ascertained through the application of tractography, a technique employed to identify fiber pathways linking one brain region to another. The distance between any pair of nodes, A and B, along a given fiber pathway is determined by calculating the number of nodes traversed by the fiber pathway. The degree of a node is defined as the count of incident edges associated with it. Nodes exhibiting a high degree are presumed to engage, either structurally or functionally, with numerous other nodes within the network.

In weighted graphs, edges are assigned weights, while binary graphs only indicate edge presence or absence. The lack of a clear biological weighting metric in DTI data complicates comprehensive analysis and interpretation[73]. We therefore assessed only binary graphs for our graph theoretical measures.

DTI Tractography The diffusion data were reconstructed using Generalized Q-sampling Imaging (GQI).[74] The deterministic fiber tracking based on the Euler method[75] was aided with quantitative anisotropy (QA) to measure the amount of anisotropic diffusion along the direction of the fiber in the fiber tracking algorithm. QA is defined as QA=Z_0 (φ(aˆ)-iso(φ)), where aˆ is the fiber orientation, φ is the spin distribution function, iso(φ) is the isotropic diffusion in the background, and Z_0 is a normalization constant that normalizes diffusion to 1 for freely diffusing water. Fiber tracking was conducted throughout the brain using 50,000 seed points and a diffusion sampling length ratio of 1.25. Restricted diffusion was quantified using restricted diffusion imaging,[76] and fiber tracking was conducted with an angular threshold of 30° and step size of 0.94mm. Fibers with QA values smaller than the

© 2025 Peterson BS et al. *JAMA Neurology*.

prespecified threshold (i.e, QA<0.092) were removed to reduce noise, and tracts shorter than 10 mm or longer than 800 mm were discarded. The orientation of the currently tracked fiber was estimated as the orientation most similar to its orientation in the previous voxel, with a change in orientation smaller than the predefined threshold.

We used the Automated Anatomical Labeling atlas[77] to parcellate the brain into 116 regions in DSI studio. The atlas was normalized into the coordinate space of the diffusion-weighted images for each participant. The weighted connectivity matrix was computed as the number of tracts beginning in one region and ending in another in the diffusion tensor space. The weighted matrix can be analyzed directly or thresholded, such that two brain regions are considered connected only if they have a minimum number of connecting tracts. Connections were binarized rather than weighted to simplify the calculation and interpretation of network measures,[78] and therefore they represent only the absence or presence of a connection rather than a continuously varying measure of connectivity. We prioritized calculation of commonly used network measures.[78]

<u>Choice of Matrix Threshold</u> To obtain a binary connectivity matrix, a threshold was applied to the weighted connectivity matrix. The threshold was chosen as the fraction of the maximum number of tracts between any two regions in the weighted connectivity matrix. Connectome measures were found to vary with different thresholds,[73] so the analysis was conducted at multiple thresholds ranging from 0.05 to 0.5. We used a threshold of 0.2, as it is a good indicator of white matter connectivity. An alternative approach would have been to generate connectivity matrices with similar values for a specified network property,[79] but we elected not to do so because brain connectivity could vary systematically with exposure levels. Therefore, we applied the same set of thresholds to all participants.

<u>Quality Control</u> Although variations in thresholds affect connectome measures, the intercorrelations among connectome measures should be similar at all thresholds. We considered connectome measures whose correlations with other connectome measures vary across thresholds as invalid, but correlations for connectome measures at each threshold did not differ significantly across all 3 thresholds and therefore were considered valid.

Connectome (Graph Theoretical) Measures[73,78,80,81]

<u>Degree</u> (k_i) of a node i represents the count of edges connecting node i to any other node j in the graph. Mathematically, $k_i$is defined as the sum of a_{ij} for all j in the set N of nodes: $k_i = \sum_{j \in N} a_{ij}.$ Here, a_{ij} is assigned a value of 1 when node i is connected to node j; otherwise, a_{ij} is set to 0. In this representation, N denotes the collection of nodes, while E signifies the set of edges within the graph.

<u>Edge Density</u>, also referred to as "connectivity," quantifies the degree to which a graph's edge count approaches its maximum possible edges. A graph with few edges is deemed "sparse." Edge density is computed as:

$$Density = 2|E|/(|N|\,(|N|-1)\,)$$

Here, $|E|$denotes the total number of edges, and $|N| = n$ represents the node count within the graph.

<u>Characteristic Path Length</u>, a measure of network integration, is the average shortest path length d_{ij} between all node pairs (i,j) within a network.[82] Low values suggest shorter distances between nodes, potentially enhancing internode communication efficiency. This metric reflects the effectiveness of information transfer and is sensitive to the presence of long paths. It is defined as the average shortest path length between all pairs of nodes in the graph.[82] In a weighted graph, the shortest path length refers to the minimum sum of edge weights connecting any two nodes, while in an unweighted graph, it corresponds to the minimum number of edges traversed. Mathematically, the characteristic path length can be expressed as:

$$L = 1/n \sum_{i \in N} L_i \; = 1/n \sum_{i \in N} \left(\sum_{i \in N, j \neq i} d_{ij}\right)/(n-1)$$

where $L_i$is the average distance between node i and all other nodes. <u>Local efficiency</u> is a key topological metric that quantifies the efficiency of information transfer within the immediate neighborhood of each node in a theoretical graph, particularly relevant to the study of brain networks[83]. It is defined as the average of the inverse shortest path lengths between all pairs of neighbors for a given node, normalized by the number of possible connections in the neighborhood. In essence, local efficiency assesses the capacity of a network to maintain efficient communication between adjacent nodes even when the central node is removed. This resilience to node failure is especially important in understanding the robustness and functional segregation of brain networks, as it reflects the network's ability to adapt and reorganize in response to external perturbations or internal damages, such as lesions or neurodegenerative processes.

$$E_{loc} = \frac{1}{n}\sum_{i \in N} E_{loc,i} = \frac{1}{n}\sum_{i \in N} \frac{\sum_{j,h \in N, j \neq i} a_{ij}\left[d_{jh}(N_i)\right]^{-1}}{K_i(K_i - 1)}$$

<u>Global Efficiency</u> serves as an additional measure of network integration, reflecting potential information exchange and integrated processing based on established structural connections. It is computed as the average of the inverse shortest path length between two nodes within the network. The distance d_{ij} between nodes i and j in a graph

© 2025 Peterson BS et al. *JAMA Neurology*.

corresponds to the number of edges in their shortest path. The efficiency E between nodes i and j is defined as the inverse of d_ij. For a network G containing n nodes, Global Efficiency can be calculated as:

$$E(G) = 1/n(n-1) \sum\_(i \neq j \in G) 1/d(i,j)$$

Global Efficiency is generally favored over Characteristic Path Length, particularly for disconnected networks, as disconnected nodes possess infinite path lengths and consequently have zero efficiency.[84] While Characteristic Path Length is predominantly influenced by long paths (with infinitely long paths being the extreme case), Global Efficiency is primarily determined by short paths, which are likely most effective for internode communication.

Transitivity (T) characterizes the connectivity of nodes to their neighbors, with higher values indicating the presence of numerous strongly connected communities.[85] It is related to the clustering coefficient (described next), but it is particularly influenced by high degree nodes**.** It is calculated as the ratio of the number of existing triplets in the network to the number of possible triplets, where a triplet is a set of 3 nodes that are connected with either two (open triplet) or three (closed triplet or triangle) edges **(Figure 1)**.

Transitivity T is computed as

$$T = \frac{\sum_{i \in N} 2t_i}{\sum_{i \in N} k_i(k_i - 1)}$$

where t_i is the number of triangles around a node i, which is computed as $t_i = \frac{1}{2} \sum_{j,h \in N} a_{ij} a_{ih} a_{jh}$.

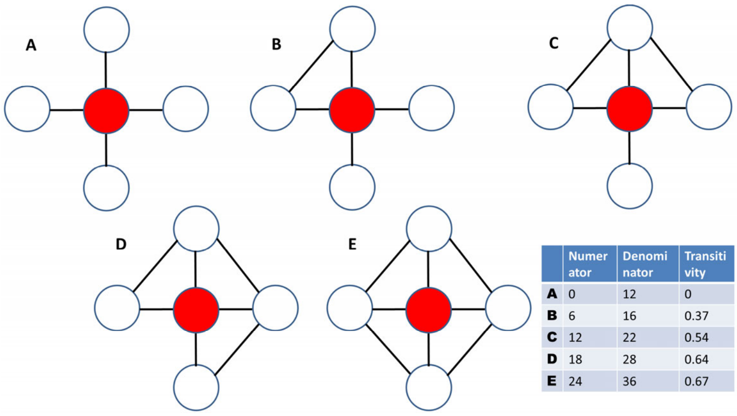

	Numerator	Denominator	Transitivity
A	0	12	0
B	6	16	0.37
C	12	22	0.54
D	18	28	0.64
E	24	36	0.67

Figure 1. Visualizing Variation in Transitivity as the number of edges between nodes varies within a network. Transitivity equals 0 when no triangles are present (**A**), and it increases with an increasing number of edges. The maximum value equals 1 for a completely connected network.

Average Clustering Coefficient measures the tendency of nodes to form clusters. It is calculated as the ratio of closed triplets (or 3 times the number of triangles) to the total possible triplets (both open and closed) surrounding a node **(Figure 2)**. A high coefficient indicates network robustness or resilience against random damage.

The clustering coefficient C_i at node i is computed as $C\_i = (2t\_i)/(k\_i\ (k\_i - 1))$, while the average clustering coefficient is given by $C = 1/n \sum\_(i \in N) C\_i$. Since the clustering coefficient is normalized for each node by the potential number of its triplets, it is disproportionately influenced by nodes with low degrees. In contrast, transitivity, which is normalized by the sum of possible triplets across all nodes, represents the network's global property.

Transitivity can be high and the average clustering coefficient low when triangles are dispersed and not interconnected. Therefore, the average clustering coefficient serves as a measure of local segregation, also known as "local efficiency",[83,84] or the average of the local efficiencies of all nodes in the graph. Neighbors that are densely interconnected form clusters around individual nodes.

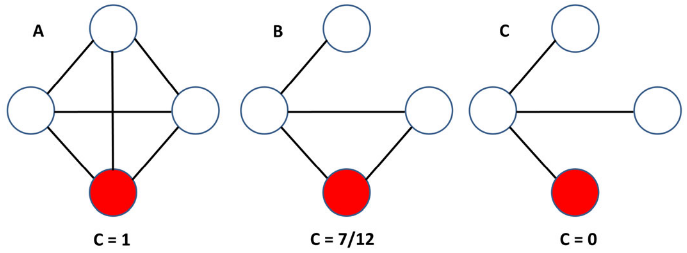

Figure 2. Visualizing Variation in Clustering Coefficient with a decreasing number of edges between nodes in the network. A completely connected network has a clustering coefficient of 1 (**A**), whereas a network with no connecting triangles has a clustering coefficient of 0 (**C**).

Rich-Club K Network quantifies the connections among higher-degree nodes. The k value determines the node count threshold (ranging from 5 to 25 in increments of 5) for calculating the rich club index. Nodes with higher degrees frequently interconnect, forming a rich club. $E\_(> k)$ represents the number of edges between nodes with a degree greater than or equal to k, while $N\_(> k)$ denotes the number of nodes with a degree greater than or equal to k. The rich-club network is computed as:

$$Rc(k) = (2E\_(> k))/(N\_(> k)\ (N\_(> k) - 1))$$

The brain's rich club is believed to facilitate a significant portion of its global communication by integrating information across separate communities and networks. Although rich club connections promote high efficiency in

global communication, they often cover long distances, consuming valuable resources such as brain volume, material, and energy.[86]

Small Worldness characterizes the extent to which a given node's neighbors are likely to be neighbors themselves while concurrently maintaining that most nodes can be reached from any other node with a minimal number of hops or steps. Consequently, Small Worldness relies on both a high clustering coefficient (or global transitivity) and average shortest path length (global efficiency). Small-world networks simultaneously exhibit high segregation and integration.

Formally, small-world networks are defined as those that possess significantly higher clustering than random networks while maintaining a characteristic path length comparable to those of random networks[82]. Small Worldness S is calculated as $S = (C/L)/(C\_rand/L\_rand\ )$, where C represents the average clustering coefficient and L denotes the characteristic path length of the brain network; C_rand i is the average clustering coefficient and L_rand is the characteristic path length of a random network with an equivalent number of nodes and edges. This measure, however, can erroneously indicate a small-world topology in highly segregated yet poorly integrated networks. Consequently, Small Worldness should not be considered a substitute for assessing integration and segregation independently.

Diameter refers to the average maximum distance between any two nodes within a graph. It can be conceptualized as the distance from a node to the most remote node in the network.

Intercorrelation Among DTI Graph Theoretical Measures

Because global efficiency in the DTI dataset correlated highly with edge density (r = 0.98), small worldness (r=0.97), and rich-club measures k10, k15, and k20 (r's>0.90), we did not include these latter measures in our analyses. We therefore retained for analyses: global efficiency, average clustering coefficient, transitivity, assortativity, diameter, radius, and degree.

rs-fMRI Graph Theory Measures

We used in-house software to calculate connectivity matrices from the smoothed rs-fMRI volumes for each participant. Briefly, we first applied the same Automated Anatomical Labeling atlas[77] to parcellate the brain into 116 ROIs, as we used for DTI connectivity analyses. We then averaged the time courses of all voxels within each ROI to yield a single time course for each ROI. We then reduced non-neuronal sources of variance[87,88] from each ROI time course by applying a temporal band-pass filter (0.01 Hz < f < 0.1 Hz) to the time course and then using multiple linear regression to remove signal from the ventricles and white matter. The residual time course was rescaled to zero mean and unit variance. Finally, we computed the connectivity matrix as pairwise Pearson's correlation coefficients of the residual time courses for all ROIs. The connectivity matrix was computed using fMRI data from the run that had the least motion artifact and outlying volumes, thereby minimizing artifactual connectivity between ROIs due to either motion artifacts or changing volume intensity across runs.

Finally, we applied the Brain Connectivity Toolbox (BCT)[89] to the connectivity matrix of each participant to compute graph theory (GT) measures. GT measures were computed for binarized matrices that were generated by thresholding the connectivity matrices at correlation coefficients that varied from 0.0 to 0.9 in 0.01 increments. We then computed the average graph theory measure as the area under the curve (AUC), thereby generating a measure that was not sensitive to the selection of a specific connectivity matrix threshold. This AUC value for each GT measure was used in statistical analyses.

Most GT measures that we calculated used the same formulae as detailed above for DTI GT measures. These were: edge density, global efficiency, network diameter, network radius, characteristic path length, transitivity, and number of edges. We also calculated the additional measure, Community Structure, as follows.

Modularity Statistic for Detecting Node Communities Community structure represents discrete clusters of nodes in a network such that, within a cluster, nodes are densely connected, but they are also loosely connected to nodes in other clusters. Thus, high community structure subdivides a network into non-overlapping clusters such that within-cluster edges are maximized and between-cluster edges are minimized. This measure was computed using Newman's spectral community detection algorithm.[90]

Intercorrelation Among rs-fMRI Graph Theoretical Measures For rs-fMRI GT measures, community structure correlated highly with maximized modularity (r>0.99), and density correlated perfectly with the number of edges (r=1.0). We therefore dropped modularity and edges from further analyses, leaving community structure, density, global efficiency, diameter, radius, characteristic path length, and transitivity for analysis.

© 2025 Peterson BS et al. *JAMA Neurology*.

ONLINE-ONLY DISCUSSION

Additional Relation of Findings to Animal Models

Cortical thickening is consistent with findings from several preclinical studies of prenatal CPF exposure. For example, CPF in cell culture promoted dendritic growth but inhibited axonal growth in developing neurons,[91-96] which could produce cortical thickening and white matter volume reductions, respectively. Further, gestational CPF exposure of fetal rats reduced neuron and glial cell counts by 20-30% in anterior cingulate and other medial prefrontal cortices,[97] and it disrupted glia and neuron distributions in the septal nucleus, striatum, somatosensory cortex, and hippocampus.[98,99] Exposure of fetal guinea pigs for 10 days[100] during a period of rapid myelination[101-103] yielded lower volumes of frontal regions (4.8%) and striatum (8.3%) and impaired learning and memory by postnatal day 10.

Somatotopy of the Internal Capsule

Fibers in the anterior limb interconnect frontal cortices with the basal ganglia and thalamus to support higher-order cognitive and executive functions: fibers in the genu project from motor cortices to cranial nerve nuclei to control head and face movement; fibers in the anterior portion of the posterior limb project from motor cortex to the medulla and spinal cord, controlling upper and lower extremity movements.[104,105] Higher prenatal CPF exposure in this same sample was previously reported to associate significantly with the presence of tremor in both arms,[106] and we found significant associations of exposure with motor performance on the finger tapping and sequencing tasks, consistent with FA and ADC findings suggesting disturbances in motor portions of the IC.

Additional Discussion of Inflammation and Oxidative Stress

Preclinical studies have demonstrated that inflammation and oxidative stress mediate many of the cellular effects of CPF,[107-113] $PM_{2.5}$,[114-116] and PAH.[117-121] Inflammation and oxidative stress during critical periods have profound consequences for fetal brain development. For example, they impair mitochondrial functioning,[122-128] which in turn produces more inflammation and oxidative stress, leading to a vicious cycle of inflammation and metabolic dysfunction.[124-126,129]

One cell type that is exquisitely sensitive to the effects of inflammation, oxidative stress, and mitochondrial dysfunction are pre-oligodendrocytes (preOLs),[129-131] which from 23-32 weeks of human gestation account for 90% of all cells in the oligodendrocyte lineage.[132] Toxicity-induced degeneration of preOLs triggers a robust, compensatory proliferation of OL progenitors that regenerates and augments the preOL pool to super-normal levels, though these new preOLs fail to differentiate into myelinating OLs despite the presence of intact nearby axons. Because OLs support axonal metabolism and functioning,[133-136] altered myelination can disrupt the maturation of axons, which in turn may disrupt activity-dependent maturation of cortical gray matter[126,132,137] and contribute to the altered gray/white matter interface we observed. Conversely, NAA contributes to signaling between neurons and oligodendrocytes, and it participates in myelin synthesis by oligodendrocytes.[138] The lower density of healthy neurons that lower NAA levels represent in our sample could alter myelin synthesis throughout development.

Consistent with this proposed model of pathogenesis, a prior study showed that CPF administered to developing rats in vivo during the period of peak of glial cell replication (PN11-14) reduced glial cell densities across the brain, with a subsequent rebound increase in glial cells by PN30.[139] Similarly, CPF exposure in newborn rats from postnatal day 11-14 activated inflammatory pathways, increased numbers of microglia and astrocytes in the substantia nigra, and reduced the numbers of dopaminergic neurons, lasting into adulthood.[140] CPF-induced inflammation and oxidative stress likely also have direct toxic effects on neuronal development, either by killing neurons or disrupting their proliferation, differentiation, and apoptosis.[96,126,141-146]

© 2025 Peterson BS et al. *JAMA Neurology*.

SUPPLEMENTAL REFERENCES

1. Whyatt RM, Camann DE, Kinney PL, et al. Residential pesticide use during pregnancy among a cohort of urban minority women. *Environmental health perspectives*. May 2002;110(5):507-14. doi:10.1289/ehp.02110507
2. Mayer SE, Jencks C. Poverty and the Distribution of Material Hardship. *The Journal of Human Resources*. 1989;24(1):88-114.
3. Bradley RH. The Home Inventory: review and reflections. *Advances in child development and behavior*. 1994;25:241-88. doi:10.1016/s0065-2407(08)60054-3
4. Jones PC, Pendergast LL, Schaefer BA, et al. Measuring home environments across cultures: Invariance of the HOME scale across eight international sites from the MAL-ED study. *J Sch Psychol*. Oct 2017;64:109-127. doi:10.1016/j.jsp.2017.06.001
5. Rubin DB. *Multiple Imputation for Nonresponse in Surveys*. John Wiley & Sons, Inc; 1987.
6. Ananth CV, Kioumourtzoglou MA, Huang Y, et al. Exposures to air pollution and risk of acute-onset placental abruption: a case-crossover study. *Epidemiology*. Sep 2018;29(5):631-638. doi:10.1097/EDE.0000000000000859
7. Perera FP, Rauh V, Tsai WY, et al. Effects of transplacental exposure to environmental pollutants on birth outcomes in a multiethnic population. *Environ Health Perspect*. Feb 2003;111(2):201-5.
8. Choi H, Jedrychowski W, Spengler J, et al. International studies of prenatal exposure to polycyclic aromatic hydrocarbons and fetal growth. *Environ Health Perspect*. Nov 2006;114(11):1744-50. doi:10.1289/ehp.8982
9. Rundle A, Hoepner L, Hassoun A, et al. Association of childhood obesity with maternal exposure to ambient air polycyclic aromatic hydrocarbons during pregnancy. *Am J Epidemiol*. Jun 1 2012;175(11):1163-72. doi:10.1093/aje/kwr455
10. Choi H, Perera F, Pac A, et al. Estimating individual-level exposure to airborne polycyclic aromatic hydrocarbons throughout the gestational period based on personal, indoor, and outdoor monitoring. *Environ Health Perspect*. Nov 2008;116(11):1509-18. doi:10.1289/ehp.10972
11. Bernert JT, Jr., Turner WE, Pirkle JL, et al. Development and validation of sensitive method for determination of serum cotinine in smokers and nonsmokers by liquid chromatography/atmospheric pressure ionization tandem mass spectrometry. *Clin Chem*. Dec 1997;43(12):2281-91.
12. Kemp S, Korkman M, Kirk U. Developmental Neuropsychological Assessment, Second Edition (NEPSY-II). Pearson, Inc.; 2007.
13. Tiffen J. *Purdue Pegboard Test*. Science Research Associates; 1968.
14. Conners CK, Staff M, Connelly V, Campbell S, MacLean M, Barnes J. Conners' continuous performance Test II (CPT II v. 5). *Multi-Health Syst Inc*. 2000;29(1):175-196.
15. Wechsler D. *Wechsler Intelligence Scale for Children-4th Edition*. Pearson; 2003.
16. Achenbach T, Rescorla L. Manual for the ASEBA School-Age Forms & Profiles. Burlington, VT: University of Vermont, Research Center for Children, Youth & Families; 2001.
17. Poznanski EO, Freeman LN, Mokros HB. Children's Depression Rating Scale Revised. *Psychopharmacology Bulletin*. 1985;21:979-989.
18. Reynolds CR, Richmond BO. Revised Children's Manifest Anxiety Scale™, Second Edition (RCMAS™-2). Los Angeles, CA: WPS; 2008.
19. DuPaul GJ, Power TJ, Anastopoulos AD, Reid R. *ADHD Rating Scale-5 for children and adolescents: Checklists, norms, and clinical interpretation*. Guilford Press; 2016.
20. Constantino JN, Davis S, Todd R, et al. Validation of a brief quantitative measure of autistic traits: Comparison of the Social Responsiveness Scale with the Autism Diagnostic Interview-Revised. *Journal of Autism and Developmental Disorders*. 2003;33:427-433.
21. Wong EC, Buxton RB, Frank LR. Quantitative imaging of perfusion using a single subtraction (QUIPSS and QUIPSS II). *Magn Reson Med*. May 1998;39(5):702-8.
22. Sled JG, Zijdenbos AP, Evans AC. A nonparametric method for automatic correction of intensity nonuniformity in MRI data. *IEEE transactions on medical imaging*. Feb 1998;17(1):87-97. doi:10.1109/42.668698
23. Shattuck DW, Leahy RM. BrainSuite: An Automated Cortical Surface Identification Tool. *Medical Image Analysis*. 2002;8(2):129-142.

© 2025 Peterson BS et al. *JAMA Neurology*.

24. Shrout PE, Fleiss JL. Intraclass correlations: uses in assessing rater reliability. *Psychol Bull*. 1979;86:420-428.
25. Haralick R, Shapiro L. *Computer and Robot Vision, volume 1*. Addison-Wesley Publishing Company; 1992.
26. Peterson BS. Form determines function: new methods for identifying the neuroanatomical loci of circuit-based disturbances in childhood disorders. *J Am Acad Child Adolesc Psychiatry*. Jun 2010;49(6):533-8. doi:10.1016/j.jaac.2010.03.010
27. Bansal R, Staib LH, Whiteman R, Wang YM, Peterson BS. ROC-based assessments of 3D cortical surface-matching algorithms. *Neuroimage*. Jan 01 2005;24(1):150-62. doi:10.1016/j.neuroimage.2004.08.054
28. Wells WM, 3rd, Viola P, Atsumi H, Nakajima S, Kikinis R. Multi-modal volume registration by maximization of mutual information. *Med Image Anal*. Mar 1996;1(1):35-51. doi:10.1016/s1361-8415(01)80004-9
29. Christensen GE, Rabbitt RD, Miller MI. Deformable templates using large deformation kinematics. *IEEE Trans Image Process*. 1996;5(10):1435-47. doi:10.1109/83.536892
30. Ono M, Kubik S, Abernathey CD. *Atlas of the Cerebral Sulci*. Thieme Medical Publishers; 1990.
31. Holmes CJ, Hoge R, Collins L, Woods R, Toga AW, Evans AC. Enhancement of MR images using registration for signal averaging. *J Comput Assist Tomogr*. Mar-Apr 1998;22(2):324-33. doi:10.1097/00004728-199803000-00032
32. Leow AD, Yanovsky I, Chiang MC, et al. Statistical properties of Jacobian maps and the realization of unbiased large-deformation nonlinear image registration. *IEEE Trans Med Imaging*. Jun 2007;26(6):822-32. doi:10.1109/TMI.2007.892646
33. Jenkinson M, Bannister P, Brady M, Smith S. Improved optimization for the robust and accurate linear registration and motion correction of brain images. *Neuroimage*. Oct 2002;17(2):825-41.
34. Power JD, Mitra A, Laumann TO, Snyder AZ, Schlaggar BL, Petersen SE. Methods to detect, characterize, and remove motion artifact in resting state fMRI. *NeuroImage*. Jan 1 2014;84:320-41. doi:10.1016/j.neuroimage.2013.08.048
35. Haselgrove JC, Moore JR. Correction for distortion of echo-planar images used to calculate the apparent diffusion coefficient. *Magnetic resonance in medicine*. Dec 1996;36(6):960-4. doi:10.1002/mrm.1910360620
36. Yeh FC, Tseng WYI. NTU-90: A high angular resolution brain atlas constructed by q-space diffeomorphic reconstruction. *NeuroImage*. Sep 1 2011;58(1):91-99. doi:10.1016/j.neuroimage.2011.06.021
37. Celtikci P, Fernandes-Cabral DT, Yeh FC, Panesar SS, Fernandez-Miranda JC. Generalized q-sampling imaging fiber tractography reveals displacement and infiltration of fiber tracts in low-grade gliomas. *Neuroradiology*. Mar 2018;60(3):267-280. doi:10.1007/s00234-018-1985-5
38. Yeh FC, Wedeen VJ, Tseng WYI. Generalized q-Sampling Imaging. *IEEE Transactions on Medical Imaging*. Sep 2010;29(9):1626-1635. doi:10.1109/Tmi.2010.2045126
39. Alexander AL, Lee JE, Lazar M, Field AS. Diffusion tensor imaging of the brain. Research Support, N.I.H., Extramural Research Support, Non-U.S. Gov't Review. *Neurotherapeutics*. Jul 2007;4(3):316-29. doi:10.1016/j.nurt.2007.05.011
40. Dong ZC, Peterson B. The rapid and automatic combination of proton MRSI data using multi-channel coils without water suppressionl. *Magnetic Resonance Imaging*. Oct 2007;25(8):1148-1154. doi:10.1016/j.mri.2007.01.005
41. Hao X, Xu D, Bansal R, et al. Multimodal magnetic resonance imaging: The coordinated use of multiple, mutually informative probes to understand brain structure and function. *Hum Brain Mapp*. Feb 2013;34(2):253-71. doi:10.1002/hbm.21440
42. Gruber B, Froeling M, Leiner T, Klomp DWJ. RF coils: A practical guide for nonphysicists. *Journal of magnetic resonance imaging : JMRI*. Jun 13 2018;48(3):590-604. doi:10.1002/jmri.26187
43. El-Sharkawy AM, Schär M, Bottomley PA, Atalar E. Monitoring and correcting spatio-temporal variations of the MR scanner's static magnetic field. *Magma (New York, NY)*. Nov 2006;19(5):223-36. doi:10.1007/s10334-006-0050-2
44. Lebon V, Petersen KF, Cline GW. Astroglial contribution to brain energy metabolism in humans revealed by 13C nuclear magnetic resonance spectroscopy: elucidation of the dominant pathway for neurotransmitter glutamate repletion and measurement of astrocytic oxidative metabolism. *J Neurosci*. 2002;22(5):1523-1531.
45. Viola P, Wells, W. M. Alignment by Maximization of Mutual Information. 1995:16-23.

© 2025 Peterson BS et al. *JAMA Neurology*.

46. Floyd TF, Ratcliffe SJ, Wang J, Resch B, Detre JA. Precision of the CASL-perfusion MRI technique for the measurement of cerebral blood flow in whole brain and vascular territories. *J Magn Reson Imaging*. Dec 2003;18(6):649-55.
47. Detre JA, Wang J. Technical aspects and utility of fMRI using BOLD and ASL. *Clin Neurophysiol*. May 2002;113(5):621-34.
48. Detre JA, Zhang W, Roberts DA, et al. Tissue specific perfusion imaging using arterial spin labeling. *NMR Biomed*. Mar 1994;7(1-2):75-82.
49. Wong EC, Buxton RB, Frank LR. Quantitative perfusion imaging using arterial spin labeling. *Neuroimaging Clin N Am*. May 1999;9(2):333-42.
50. Lu H, Clingman C, Golay X, van Zijl PC. Determining the longitudinal relaxation time (T1) of blood at 3.0 Tesla. *Magn Reson Med*. Sep 2004;52(3):679-82. doi:10.1002/mrm.20178
51. Jarnum H, Steffensen EG, Knutsson L, et al. Perfusion MRI of brain tumours: a comparative study of pseudo-continuous arterial spin labelling and dynamic susceptibility contrast imaging. *Neuroradiology*. Apr 2010;52(4):307-17. doi:10.1007/s00234-009-0616-6
52. Alsop DC, Detre JA. Reduced transit-time sensitivity in noninvasive magnetic resonance imaging of human cerebral blood flow. *J Cereb Blood Flow Metab*. Nov 1996;16(6):1236-49.
53. Christensen GE, Joshi SC, Miller MI. Volumetric Transformation of Brain Anatomy. *IEEE Transactions on Medical Imaging*. 1997;16(6):1369-1383.
54. Peterson BS, Warner V, Bansal R, et al. Cortical thinning in persons at increased familial risk for major depression. *Proc Natl Acad Sci U S A*. Apr 14 2009;106(15):6273-8. doi:0805311106 [pii]

10.1073/pnas.0805311106
55. Power JD, Barnes KA, Snyder AZ, Schlaggar BL, Petersen SE. Spurious but systematic correlations in functional connectivity MRI networks arise from subject motion. *Neuroimage*. Feb 1 2012;59(3):2142-54. doi:10.1016/j.neuroimage.2011.10.018
56. Nieto-Castanon A. *Handbook of functional connectivity Magnetic Resonance Imaging methods in CONN*. Hilbert Press; 2020.
57. Andersson JL, Hutton C, Ashburner J, Turner R, Friston K. Modeling geometric deformations in EPI time series. Research Support, Non-U.S. Gov't. *Neuroimage*. May 2001;13(5):903-19. doi:10.1006/nimg.2001.0746
58. Henson RNA, Buechel C, Josephs O, Friston KJ. The slice-timing problem in event-related fMRI. *NeuroImage*. 1999;9:125.
59. Ashburner J, Friston KJ. Unified segmentation. Research Support, Non-U.S. Gov't. *NeuroImage*. Jul 1 2005;26(3):839-51. doi:10.1016/j.neuroimage.2005.02.018
60. Johnson NL. Systems of frequency curves generated by methods of translation. *Biometrika*. Jun 1949;36(Pt. 1-2):149-76.
61. Burbidge JB, Magee L, Robb AL. Alternative transformations to handle extreme values of the dependent variable. *Journal of the American Statistical Association*. 1988;83(401):123-127. doi:10.2307/2288929
62. Friston KJ, Worsley KJ, Frackowiak RS, Mazziotta JC, Evans AC. Assessing the significance of focal activations using their spatial extent. *Hum Brain Mapp*. 1994;1(3):210-20. doi:10.1002/hbm.460010306
63. Worsley KJ, Marrett S, Neelin P, Vandal AC, Friston KJ, Evans AC. A unified statistical approach for determining significant signals in images of cerebral activation. *Hum Brain Mapp*. 1996;4(1):58-73. doi:10.1002/(SICI)1097-0193(1996)4:1<58::AID-HBM4>3.0.CO;2-O
64. Adler RJ, Hasofer AM. Level-Crossings for Random Fields. *Annals of Probability*. 1976;4(1):1-12. doi:DOI 10.1214/aop/1176996176
65. Adler RJ. Geometry of Random Fields. *Geometry of Random Fields*. 2010;62:1-280. doi:10.1137/1.9780898718980
66. Hasofer AM. Upcrossings of Random Fields. *Advances in Applied Probability*. 1978:14-21.
67. Adler RJ. Generalizing Notion of Upcrossings to Random Fields. *Advances in Applied Probability*. 1977;9(2):226-226. doi:Doi 10.2307/1426370
68. Adler RJ. Excursions above a Fixed Level by N-Dimensional Random Fields. *Journal of Applied Probability*. 1976;13(2):276-289. doi:Doi 10.2307/3212831
69. Nosko VP. The characteristics of excursions of Gaussian homogeneous random fields above a high level. 1969:216-222.
70. Zhao X, Lynch JG, Chen Q. Reconsidering Baron and Kenny: Myths and truths about mediation analysis. *Journal of Consumer Research*. 2010;37(2):197-206. doi:10.1086/651257

© 2025 Peterson BS et al. *JAMA Neurology*.

71. MacKinnon DP, Fairchild AJ, Fritz MS. Mediation analysis. *Annu Rev Psychol*. 2007;58:593-614.
72. MacKinnon DP. *Introduction to Statistical Mediation Analysis*. Lawrence Erlbaum Associates; 2008.
73. Kaiser M. A tutorial in connectome analysis: topological and spatial features of brain networks. *NeuroImage*. 2011/08// 2011;57(3):892-907. doi:10.1016/j.neuroimage.2011.05.025
74. Yeh F-C, Wedeen VJ, Tseng W-YI. Generalized ${q}$-sampling imaging. *IEEE transactions on medical imaging*. 2010;29(9):1626-1635.
75. Yeh F-C, Verstynen TD, Wang Y, Fernández-Miranda JC, Tseng W-YI. Deterministic diffusion fiber tracking improved by quantitative anisotropy. *PloS one*. 2013;8(11):e80713.
76. Yeh FC, Liu L, Hitchens TK, Wu YL. Mapping immune cell infiltration using restricted diffusion MRI. *Magnetic resonance in medicine*. 2017;77(2):603-612.
77. Tzourio-Mazoyer N, Landeau B, Papathanassiou D, et al. Automated anatomical labeling of activations in SPM using a macroscopic anatomical parcellation of the MNI MRI single-subject brain. *Neuroimage*. 2002;15(1):273-289.
78. Rubinov M, Sporns O. Complex network measures of brain connectivity: Uses and interpretations. *NeuroImage*. 2010/09/01/ 2010;52(3):1059-1069. doi:https://doi.org/10.1016/j.neuroimage.2009.10.003
79. Van Wijk BC, Stam CJ, Daffertshofer A. Comparing brain networks of different size and connectivity density using graph theory. *PloS one*. 2010;5(10):e13701.
80. Meoded A, Huisman TA, Casamassima MGS, Jallo GI, Poretti A. The structural connectome in children: basic concepts, how to build it, and synopsis of challenges for the developing pediatric brain. *Neuroradiology*. 2017;59:445-460.
81. Sporns O. Network attributes for segregation and integration in the human brain. *Current opinion in neurobiology*. 2013;23(2):162-171.
82. Watts DJ, Strogatz SH. Collective dynamics of 'small-world'networks. *nature*. 1998;393(6684):440-442.
83. Latora V, Marchiori M. Efficient Behavior of Small-World Networks. *Physical Review Letters*. 10/17/ 2001;87(19):198701. doi:10.1103/PhysRevLett.87.198701
84. Achard S, Bullmore E. Efficiency and Cost of Economical Brain Functional Networks. *PLOS Computational Biology*. 2007;3(2):e17. doi:10.1371/journal.pcbi.0030017
85. Newman MEJ. The structure and function of complex networks. *SIAM Review*. Jun 2003;45(2):167-256. doi:Pii S0036144503424804

Doi 10.1137/S003614450342480
86. Bullmore E, Sporns O. The economy of brain network organization. *Nature Reviews Neuroscience*. 2012/05/01 2012;13(5):336-349. doi:10.1038/nrn3214
87. Fox MD, Zhang DY, Snyder AZ, Raichle ME. The Global Signal and Observed Anticorrelated Resting State Brain Networks. *Journal of Neurophysiology*. Jun 2009;101(6):3270-3283. doi:10.1152/jn.90777.2008
88. Power JD, Barnes KA, Snyder AZ, Schlaggar BL, Petersen SE. Spurious but systematic correlations in functional connectivity MRI networks arise from subject motion (vol 59, pg 2142, 2012). *NeuroImage*. Nov 1 2012;63(2):999-999. doi:10.1016/j.neuroimage.2012.01.069
89. Rubinov M, Sporns O. Complex network measures of brain connectivity: uses and interpretations. Research Support, Non-U.S. Gov't. *NeuroImage*. Sep 2010;52(3):1059-69. doi:10.1016/j.neuroimage.2009.10.003
90. Newman ME. Modularity and community structure in networks. *Proc Natl Acad Sci U S A*. Jun 6 2006;103(23):8577-82. doi:10.1073/pnas.0601602103
91. Howard AS, Bucelli R, Jett DA, Bruun D, Yang D, Lein PJ. Chlorpyrifos exerts opposing effects on axonal and dendritic growth in primary neuronal cultures. *Toxicology and applied pharmacology*. Sep 01 2005;207(2):112-24. doi:10.1016/j.taap.2004.12.008
92. Yang D, Howard A, Bruun D, Ajua-Alemanj M, Pickart C, Lein PJ. Chlorpyrifos and chlorpyrifos-oxon inhibit axonal growth by interfering with the morphogenic activity of acetylcholinesterase. *Toxicology and applied pharmacology*. Apr 01 2008;228(1):32-41. doi:10.1016/j.taap.2007.11.005
93. Flaskos J, Nikolaidis E, Harris W, Sachana M, Hargreaves AJ. Effects of sub-lethal neurite outgrowth inhibitory concentrations of chlorpyrifos oxon on cytoskeletal proteins and acetylcholinesterase in differentiating N2a cells. *Toxicology and applied pharmacology*. Nov 01 2011;256(3):330-6. doi:10.1016/j.taap.2011.06.002

© 2025 Peterson BS et al. *JAMA Neurology*.

94. Das KP, Barone S, Jr. Neuronal differentiation in PC12 cells is inhibited by chlorpyrifos and its metabolites: is acetylcholinesterase inhibition the site of action? *Toxicology and applied pharmacology*. Nov 01 1999;160(3):217-30. doi:10.1006/taap.1999.8767
95. Sachana M, Flaskos J, Sidiropoulou E, Yavari CA, Hargreaves AJ. Inhibition of extension outgrowth in differentiating rat C6 glioma cells by chlorpyrifos and chlorpyrifos oxon: effects on microtubule proteins. *Toxicol In Vitro*. Aug 2008;22(5):1387-91. doi:10.1016/j.tiv.2008.02.022
96. Song X, Violin JD, Seidler FJ, Slotkin TA. Modeling the developmental neurotoxicity of chlorpyrifos in vitro: macromolecule synthesis in PC12 cells. *Toxicology and applied pharmacology*. Jul 1998;151(1):182-91. doi:10.1006/taap.1998.8424
97. Chen XP, Chao YS, Chen WZ, Dong JY. Mother gestational exposure to organophosphorus pesticide induces neuron and glia loss in daughter adult brain. *J Environ Sci Health B*. Feb 2017;52(2):77-83. doi:10.1080/03601234.2016.1239973
98. Roy TS, Seidler FJ, Slotkin TA. Morphologic effects of subtoxic neonatal chlorpyrifos exposure in developing rat brain: regionally selective alterations in neurons and glia. *Brain Res Dev Brain Res*. Feb 20 2004;148(2):197-206. doi:10.1016/j.devbrainres.2003.12.004
99. Roy TS, Sharma V, Seidler FJ, Slotkin TA. Quantitative morphological assessment reveals neuronal and glial deficits in hippocampus after a brief subtoxic exposure to chlorpyrifos in neonatal rats. *Brain Res Dev Brain Res*. Mar 22 2005;155(1):71-80. doi:10.1016/j.devbrainres.2004.12.004
100. Mullins RJ, Xu S, Pereira EF, et al. Prenatal exposure of guinea pigs to the organophosphorus pesticide chlorpyrifos disrupts the structural and functional integrity of the brain. *Neurotoxicology*. May 2015;48:9-20. doi:10.1016/j.neuro.2015.02.002
101. Dobbing J, Sands J. Growth and development of the brain and spinal cord of the guinea pig. *Brain Res*. Jan 06 1970;17(1):115-23.
102. Clancy B, Finlay BL, Darlington RB, Anand KJ. Extrapolating brain development from experimental species to humans. *Neurotoxicology*. Sep 2007;28(5):931-7. doi:10.1016/j.neuro.2007.01.014
103. Silva FMdOe, Alcantara D, Carvalho RC, et al. Development of the central nervous system in guinea pig (Cavia porcellus, Rodentia, Caviidae). *Pesquisa Veterinária Brasileira*. 2016;36:753-760.
104. Schmahmann JD, Pandya D. *Fiber Pathways of the Brain*. Oxford University Press, USA; 2009.
105. Emos MC, Khan Suheb MZ, Agarwal S. Neuroanatomy, Internal Capsule. *StatPearls*. StatPearls Publishing. Copyright © 2024, StatPearls Publishing LLC.; 2024.
106. Rauh VA, Garcia WE, Whyatt RM, Horton MK, Barr DB, Louis ED. Prenatal exposure to the organophosphate pesticide chlorpyrifos and childhood tremor. *Neurotoxicology*. Dec 2015;51:80-6. doi:10.1016/j.neuro.2015.09.004
107. Bagchi D, Bagchi M, Hassoun EA, Stohs SJ. In vitro and in vivo generation of reactive oxygen species, DNA damage and lactate dehydrogenase leakage by selected pesticides. *Toxicology*. Dec 15 1995;104(1-3):129-40.
108. Crumpton TL, Seidler FJ, Slotkin TA. Is oxidative stress involved in the developmental neurotoxicity of chlorpyrifos? *Brain Res Dev Brain Res*. Jun 30 2000;121(2):189-95.
109. Garcia SJ, Seidler FJ, Crumpton TL, Slotkin TA. Does the developmental neurotoxicity of chlorpyrifos involve glial targets? Macromolecule synthesis, adenylyl cyclase signaling, nuclear transcription factors, and formation of reactive oxygen in C6 glioma cells. *Brain Res*. Feb 09 2001;891(1-2):54-68.
110. Jett DA, Navoa RV. In vitro and in vivo effects of chlorpyrifos on glutathione peroxidase and catalase in developing rat brain. *Neurotoxicology*. Feb-Apr 2000;21(1-2):141-5.
111. Ranjbar A, Pasalar P, Abdollahi M. Induction of oxidative stress and acetylcholinesterase inhibition in organophosphorous pesticide manufacturing workers. *Hum Exp Toxicol*. Apr 2002;21(4):179-82. doi:10.1191/0960327102ht238oa
112. Zhou JF, Xu GB, Fang WJ. Relationship between acute organophosphorus pesticide poisoning and damages induced by free radicals. *Biomedical and environmental sciences : BES*. Jun 2002;15(2):177-86.
113. Tanaka J, Toku K, Zhang B, Ishihara K, Sakanaka M, Maeda N. Astrocytes prevent neuronal death induced by reactive oxygen and nitrogen species. *Glia*. Nov 1999;28(2):85-96.
114. Bolton JL, Marinero S, Hassanzadeh T, et al. Gestational exposure to air pollution alters cortical volume, microglial morphology, and microglia-neuron interactions in a sex-specific manner. *Frontiers in synaptic neuroscience*. 2017;9:10. doi:10.3389/fnsyn.2017.00010
115. Klocke C, Allen JL, Sobolewski M, et al. Neuropathological consequences of gestational exposure to concentrated ambient fine and ultrafine particles in the mouse. *Toxicological sciences : an official journal of the Society of Toxicology*. Apr 1 2017;156(2):492-508. doi:10.1093/toxsci/kfx010

© 2025 Peterson BS et al. *JAMA Neurology*.

116. Costa LG, Cole TB, Coburn J, Chang Y-C, Dao K, Roqué PJ. Neurotoxicity of traffic-related air pollution. *Neurotoxicology*. 2017/03/01/ 2017;59:133-139. doi:https://doi.org/10.1016/j.neuro.2015.11.008
117. Block ML, Elder A, Auten RL, et al. The outdoor air pollution and brain health workshop. *Neurotoxicology*. Oct 2012;33(5):972-84. doi:10.1016/j.neuro.2012.08.014
118. Dutta K, Ghosh D, Nazmi A, Kumawat KL, Basu A. A common carcinogen benzo[a]pyrene causes neuronal death in mouse via microglial activation. Research Support, Non-U.S. Gov't. *PLoS One*. 2010;5(4):e9984. doi:10.1371/journal.pone.0009984
119. Herbstman JB, Tang D, Zhu D, et al. Prenatal exposure to polycyclic aromatic hydrocarbons, benzo[a]pyrene-DNA adducts, and genomic DNA methylation in cord blood. *Environ Health Perspect*. May 2012;120(5):733-8. doi:10.1289/ehp.1104056
120. Saunders CR, Das SK, Ramesh A, Shockley DC, Mukherjee S. Benzo(a)pyrene-induced acute neurotoxicity in the F-344 rat: role of oxidative stress. *J Appl Toxicol*. Sep-Oct 2006;26(5):427-38.
121. Pereira RD, De Long NE, Wang RC, Yazdi FT, Holloway AC, Raha S. Angiogenesis in the placenta: the role of reactive oxygen species signaling. *BioMed research international*. 2015;2015:814543. doi:10.1155/2015/814543
122. Xu X, Liu C, Xu Z, et al. Long-term exposure to ambient fine particulate pollution induces insulin resistance and mitochondrial alteration in adipose tissue. *Toxicological sciences : an official journal of the Society of Toxicology*. Nov 2011;124(1):88-98. doi:10.1093/toxsci/kfr211
123. Xu Z, Xu X, Zhong M, et al. Ambient particulate air pollution induces oxidative stress and alterations of mitochondria and gene expression in brown and white adipose tissues. *Particle and Fibre Toxicology*. 2011/07/11 2011;8(1):20. doi:10.1186/1743-8977-8-20
124. Picca A, Calvani R, Coelho-Junior HJ, Landi F, Bernabei R, Marzetti E. Mitochondrial Dysfunction, Oxidative Stress, and Neuroinflammation: Intertwined Roads to Neurodegeneration. *Antioxidants*. 2020;9(8):647.
125. Kowalczyk P, Sulejczak D, Kleczkowska P, et al. Mitochondrial Oxidative Stress—A Causative Factor and Therapeutic Target in Many Diseases. *International journal of molecular sciences*. 2021;22(24):13384.
126. Gyllenhammer LE, Rasmussen JM, Bertele N, et al. Maternal inflammation during pregnancy and offspring brain development: the role of mitochondria. *Biological Psychiatry: Cognitive Neuroscience and Neuroimaging*. 2022/05/01/ 2022;7(5):498-509. doi:https://doi.org/10.1016/j.bpsc.2021.11.003
127. Singh N, Lawana V, Luo J, et al. Organophosphate pesticide chlorpyrifos impairs STAT1 signaling to induce dopaminergic neurotoxicity: Implications for mitochondria mediated oxidative stress signaling events. *Neurobiol Dis*. Sep 2018;117:82-113. doi:10.1016/j.nbd.2018.05.019
128. Yamada S, Kubo Y, Yamazaki D, Sekino Y, Kanda Y. Chlorpyrifos inhibits neural induction via Mfn1-mediated mitochondrial dysfunction in human induced pluripotent stem cells. *Scientific reports*. Jan 23 2017;7:40925. doi:10.1038/srep40925
129. Spaas J, van Veggel L, Schepers M, et al. Oxidative stress and impaired oligodendrocyte precursor cell differentiation in neurological disorders. *Cellular and Molecular Life Sciences*. 2021/05/01 2021;78(10):4615-4637. doi:10.1007/s00018-021-03802-0
130. Back SA, Han BH, Luo NL, et al. Selective vulnerability of late oligodendrocyte progenitors to hypoxia-ischemia. *J Neurosci*. Jan 15 2002;22(2):455-63. doi:10.1523/jneurosci.22-02-00455.2002
131. Butts BD, Houde C, Mehmet H. Maturation-dependent sensitivity of oligodendrocyte lineage cells to apoptosis: implications for normal development and disease. *Cell Death & Differentiation*. 2008/07/01 2008;15(7):1178-1186. doi:10.1038/cdd.2008.70
132. Volpe JJ. Dysmaturation of premature brain: importance, cellular mechanisms, and potential interventions. *Pediatric Neurology*. 2019/06/01/ 2019;95:42-66. doi:https://doi.org/10.1016/j.pediatrneurol.2019.02.016
133. Harris JJ, Attwell D. The energetics of CNS white matter. *J Neurosci*. Jan 4 2012;32(1):356-71. doi:10.1523/JNEUROSCI.3430-11.2012
134. Beirowski B. Concepts for regulation of axon integrity by enwrapping glia. *Frontiers in cellular neuroscience*. Dec 19 2013;7:256. doi:10.3389/fncel.2013.00256
135. Fruhbeis C, Frohlich D, Kuo WP, et al. Neurotransmitter-triggered transfer of exosomes mediates oligodendrocyte-neuron communication. *PLoS Biol*. Jul 2013;11(7):e1001604. doi:10.1371/journal.pbio.1001604
136. Funfschilling U, Supplie LM, Mahad D, et al. Glycolytic oligodendrocytes maintain myelin and long-term axonal integrity. *Nature*. Apr 29 2012;485(7399):517-21. doi:10.1038/nature11007
137. Ophelders DRMG, Gussenhoven R, Klein L, et al. Preterm brain injury, antenatal triggers, and therapeutics: timing Is key. *Cells*. 2020;9(8):1871.

© 2025 Peterson BS et al. *JAMA Neurology*.

138. Moffett JR, Ross B, Arun P, Madhavarao CN, Namboodiri AM. N-Acetylaspartate in the CNS: from neurodiagnostics to neurobiology. *Prog Neurobiol*. Feb 2007;81(2):89-131. doi:10.1016/j.pneurobio.2006.12.003
139. Garcia SJ, Seidler FJ, Qiao D, Slotkin TA. Chlorpyrifos targets developing glia: effects on glial fibrillary acidic protein. *Brain Res Dev Brain Res*. Feb 28 2002;133(2):151-61.
140. Zhang J, Dai H, Deng Y, et al. Neonatal chlorpyrifos exposure induces loss of dopaminergic neurons in young adult rats. *Toxicology*. Oct 02 2015;336:17-25. doi:10.1016/j.tox.2015.07.014
141. Crumpton TL, Seidler FJ, Slotkin TA. Developmental neurotoxicity of chlorpyrifos in vivo and in vitro: effects on nuclear transcription factors involved in cell replication and differentiation. *Brain Res*. Feb 28 2000;857(1-2):87-98.
142. Qiao D, Seidler FJ, Slotkin TA. Developmental neurotoxicity of chlorpyrifos modeled in vitro: comparative effects of metabolites and other cholinesterase inhibitors on DNA synthesis in PC12 and C6 cells. *Environ Health Perspect*. Sep 2001;109(9):909-13.
143. Rush T, Liu XQ, Hjelmhaug J, Lobner D. Mechanisms of chlorpyrifos and diazinon induced neurotoxicity in cortical culture. *Neuroscience*. Mar 31 2010;166(3):899-906. doi:10.1016/j.neuroscience.2010.01.025
144. Slotkin TA, Seidler FJ. Developmental neurotoxicity of organophosphates targets cell cycle and apoptosis, revealed by transcriptional profiles in vivo and in vitro. *Neurotoxicol Teratol*. Mar 2012;34(2):232-41. doi:10.1016/j.ntt.2011.12.001
145. Chen XP, Chen WF, Wang DW. Prenatal organophosphates exposure alternates the cleavage plane orientation of apical neural progenitor in developing neocortex. *PLoS One*. 2014;9(4):e95343. doi:10.1371/journal.pone.0095343
146. Chen XP, Wang TT, Wu XZ, Wang DW, Chao YS. An in vivo study in mice: mother's gestational exposure to organophosphorus pesticide retards the division and migration process of neural progenitors in the fetal developing brain. *Toxicol Res*. 2019;5(5):1359–1370.
147. *NEPSY--II Administration Manual and the NEPSY--II Clinical and Interpretation Manual*. Pearson, Inc; 2007.
148. Tuddenham RD. Theoretical regularities and individual idiosyncrasies. *Measurement and Piaget*. McGraw Hill; 1971.
149. Achenbach TM, Howell CT, McConaughy SH, Stanger C. Six-year predictors of problems in a national sample: III. Transitions to young adult syndromes. *Journal of the American Academy of Child and Adolescent Psychiatry*. 1995;34:658-669.
150. Achenbach TM, Howell CT, McConaughy SM, Stanger C. Six-year predictors of problems in a national sample of children and youth: II. Signs of disturbance. *Journal of the American Academy of Child and Adolescent Psychiatry*. 1995;34(4):488-498.
151. Perrin S, Last CG. Do childhood anxiety measures measure anxiety? *J Abn Child Psych*. 1992;20:567-578.
152. DuPaul GJ. Parent and teacher ratings of ADHD symptoms: psychometric properties in a community-based sample. *J Clin Child Psychology*. 1991;20:245-253.
153. Constantino JN. *The Social Responsiveness Scale*. Western Psychological Service; 2005.
154. Constantino JN, Davis SA, Todd RD, et al. Validation of a brief quantitative measure of austistic traits: Comparison of the Social Responsiveness Scale with the Autsim Diagnostic Interview-Revised. *J Autism Dev Disord*. 2003;33:427-433.
155. Schisterman EF, Vexler A, Whitcomb BW, Liu A. The limitations due to exposure detection limits for regression models. *Am J Epidemiol*. Feb 15 2006;163(4):374-83. doi:10.1093/aje/kwj039
156. Finkelstein MM, Verma DK. Exposure estimation in the presence of nondetectable values: another look. *Aihaj*. Mar-Apr 2001;62(2):195-8. doi:10.1080/15298660108984622
157. Leith KF, Bowerman WW, Wierda MR, Best DA, Grubb TG, Sikarske JG. A comparison of techniques for assessing central tendency in left-censored data using PCB and p,p'DDE contaminant concentrations from Michigan's Bald Eagle Biosentinel Program. *Chemosphere*. Jun 2010;80(1):7-12. doi:10.1016/j.chemosphere.2010.03.056

© 2025 Peterson BS et al. *JAMA Neurology*.

SUPPLEMENTAL TABLES

© 2025 Peterson BS et al. *JAMA Neurology*.

eTable 1: Correlation Matrix for Model Variables

		sex	Maternal Ethnicity	Maternal Education	Material Hardship	Home Stress	CPF	tPAH	LntPAH	$PM_{2.5}$	cotinine	ETS
**age**	r	.06	.03	-.03	.04	.001	.12	-.008	.03	.29	.15	.18
	p-value	.33	.67	.68	.54	.98	.06	.90	.60	<0.0001	.02	.003
	N	264	264	264	264	250	264	255	255	262	260	264
**sex**	r		-.04	-.06	-.01	-.15	.02	.04	-.01	-.04	-.06	-.008
	p-value		.56	.37	.82	.02	.81	.48	.85	.56	.37	.90
	N		264	264	264	250	264	255	255	262	260	264
**Maternal Ethnicity**	r			-.02	-.26	.19	.10	-.04	-.02	-.26	.43**	.24
	p-value			.79	<0.0001	.003	.11	.51	.70	<0.0001	<0.0001	<0.0001
	N			264	264	250	264	255	255	262	260	264
**Maternal Education**	r				-.05	.24	-.02	-.06	-.11	-.006	-.11	-.05
	p-value				.38	<0.0001	.70	.30	.08	.92	.07	.38
	N				264	250	264	255	255	262	260	264
**Material Hardship**	r					-.22	-.002	.02	.06	.22	-.07	.08
	p-value					<0.0001	.97	.82	.31	<0.0001	.23	.18
	N					250	264	255	255	262	260	264
**Home Stress**	r						-.02	-.003	-.06	-.19	.02	-.02
	p-value						.73	.96	.39	.003	.79	.80
	N						250	242	242	248	246	250
**CPF**	r							.08	.13	.03	.02	.10
	p-value							.22	.04	.63	.80	.10
	N							255	255	262	260	264
**tPAH**	r								.81	.11	.10	-.04
	p-value								<0.0001	.08	.129	.49
	N								255	253	251	255
**LntPAH**	r									.18	.13*	-.01
	p-value									.004	.05	.82
	N									253	251	255
**$PM_{2.5}$**	r										.01	.04
	p-value										.84	.56
	N										258	262
**cotinine**	r											.54
	p-value											<0.0001
	N											260

Correlations with p<0.05 are shaded. ETS=Environmental Tobacco Smoke. Serum cotinine was measured from cord blood at birth as a measure of maternal second-hand ETS exposure (maternal smoking was exclusionary at initial recruitment). Including cotinine or ETS as additional covariates in our CPF exposure models did not alter our findings.

© 2025 Peterson BS et al. *JAMA Neurology*.

eTable 2: Correlation Matrix for DTI Graph Theoretical Measures

		clustering coeff average	transitivity	characteristic path length	small-worldness	global efficiency	diameter	radius	local efficiency	assortativity coefficient	rich club k10	rich club k15	rich club k20	rich club k25	degree	cluster coef
density	R	.85**	-.15*	-.86**	.97**	.98**	-.52**	-.37**	.75**	-.46**	.97**	.97**	.93**	.83**	.55**	.30**
	P-value	.0001	.01	.0001	.0001	.0001	.0001	.0001	.0001	.0001	.0001	.0001	.0001	.0001	.0001	.0001
	N	261	261	261	261	261	261	261	261	261	261	261	261	261	261	261
clustering coeff average	R		-.15*	-.83**	.95**	.87**	-.53**	-.41**	.93**	-.56**	.78**	.85**	.84**	.83**	.57**	.36**
	P-value		.01	.0001	.0001	.0001	.0001	.0001	.0001	.0001	.0001	.0001	.0001	.0001	.0001	.0001
	N		261	261	261	261	261	261	261	261	261	261	261	261	261	261
transitivity	R			.14*	-.16**	-.16*	.04	.03	-.11	-.02	-.20**	-.23**	-.26**	-.27**	-.14*	.02
	P-value			.03	.008	.01	.54	.63	.07	.76	.001	.0001	.0001	.0001	.03	.76
	N			261	261	261	261	261	261	261	261	261	261	261	261	261
characteristic path length	R				-.90**	-.94**	.72**	.54**	-.89**	.52**	-.73**	-.87**	-.86**	-.82**	-.46**	-.36**
	P-value				.0001	.0001	.0001	.0001	.0001	.0001	.0001	.0001	.0001	.0001	.0001	.0001
	N				261	261	261	261	261	261	261	261	261	261	261	261
small-worldness	R					.98**	-.57**	-.43**	.86**	-.55**	.91**	.96**	.92**	.86**	.58**	.34**
	P-value					.0001	.0001	.0001	.0001	.0001	.0001	.0001	.0001	.0001	.0001	.0001
	N					261	261	261	261	261	261	261	261	261	261	261
global efficiency	R						-.61**	-.45**	.81**	-.50**	.92**	.97**	.93**	.85**	.54**	.33**
	P-value						.0001	.0001	.0001	.0001	.0001	.0001	.0001	.0001	.0001	.0001
	N						261	261	261	261	261	261	261	261	261	261
diameter of graph	R							.47**	-.64**	.37**	-.39**	-.52**	-.52**	-.49**	-.29**	-.28**
	P-value							.0001	.0001	.0001	.0001	.0001	.0001	.0001	.0001	.0001
	N							261	261	261	261	261	261	261	261	261
radius of graph	R								-.45**	.25**	-.28**	-.35**	-.33**	-.29**	-.20**	-.21**
	P-value								.0001	.0001	.0001	.0001	.0001	.0001	.001	.001
	N								261	261	261	261	261	261	261	261
local efficiency	R									-.54**	.61**	.76**	.76**	.78**	.48**	.38**
	P-value									.0001	.0001	.0001	.0001	.0001	.0001	.0001
	N									261	261	261	261	261	261	261
assortativity coefficient	R										-.41**	-.45**	-.42**	-.39**	-.38**	-.23**
	P-value										.0001	.0001	.0001	.0001	.0001	.0001
	N										261	261	261	261	261	261
rich club k10	R											.95**	.90**	.79**	.54**	.24**
	P-value											.0001	.0001	.0001	.0001	.0001
	N											261	261	261	261	261
rich club k15	R												.98**	.90**	.55**	.31**
	P-value												.0001	.0001	.0001	.0001
	N												261	261	261	261
rich club k20	R													.94**	.53**	.30**
	P-value													.0001	.0001	.0001
	N													261	261	261
rich club k25	R														.50**	.33**

© 2025 Peterson BS et al. *JAMA Neurology*.

	P-value														.0001	.0001
	N														261	261
degree	R															-.38**
	P-value															.0001
	N															261

Shaded cells indicate Pearson’s r>0.90

© 2025 Peterson BS et al. *JAMA Neurology*.

eTable 3: Correlation Matrix for rs-fMRI Graph Theoretical Measures

Correlations AUC									
		Density	Global Efficiency	Maximized Modularity	Network Diameter	Network Radius	Characteristic Path Length	Transitivity	# Edges
Community Structure Statistic	R	-.87**	-.85**	1.00**	.43**	-.27**	.39**	-.41**	-.87**
	P-value	.0001	.0001	.0001	.0001	.0001	.0001	.0001	.0001
	N	297	297	297	297	297	297	297	297
Density	R		.99**	-.87**	-.49**	.31**	-.46**	.31**	1.000**
	P-value		.0001	.0001	.0001	.0001	.0001	.0001	.0001
	N		297	297	297	297	297	297	297
Global Efficiency	R			-.86**	-.48**	.33**	-.42**	.29**	.99**
	P-value			.0001	.0001	.0001	.0001	.0001	.0001
	N			297	297	297	297	297	297
Maximized Modularity	R				.43**	-.27**	.39**	-.41**	-.87**
	P-value				.0001	.0001	.0001	.0001	.0001
	N				297	297	297	297	297
Network Diameter	R					-.24**	.88**	-.08	-.49**
	P-value					.0001	.0001	.183	.0001
	N					297	297	297	297
Network Radius	R						-.16**	.05	.31**
	P-value						.005	.405	.0001
	N						297	297	297
Network Characteristic Path Length	R							-.17**	-.46**
	P-value							.004	.0001
	N							297	297
Transitivity	R								.31**
	P-value								.0001
	N								297

Shaded cells indicate Pearson's r>0.90

© 2025 Peterson BS et al. *JAMA Neurology*.

eTable 4: Association of CPF Exposure with Neuropsychological Test Scores

Test	Interpretation of Higher Scores	Construct Assessed	Unstandardized Coefficient	Standardized Coefficient	95% Confidence Interval for B		Test Statistics		
			B	Std. Error	Lower Bound	Upper Bound	Beta	t	p-value
**Sensorimotor Functioning -- NEPSY Finger Tapping[147]**									
Dominant Hand Combined Completion Time	Better	Dominant hand fine motor control and programming	-.21	0.04	-.29	-.13	-.30	-5.0	<0.0001
NonDominant Hand Combined Completion Time	Better	Non-dominant hand fine motor control and programming	-.22	0.04	-.30	-.13	-.30	-4.84	<0.0001
Dominant + Non-dominant Repetitions Completion Time Combined	Better	Overall fine motor control and programming	-.20	0.04	-.28	-.12	-.29	-4.71	<0.0001
Dominant vs Nondominant Contrast	dominant < non-dominant hand	Imbalance between hands	-.10	0.05	-.20	-0.006	-.13	-2.10	0.04
Dominant + Non-dominant Sequences Completion Time Combined	Better	Motor programming	-.20	0.04	-.28	-.11	-.27	-4.36	<0.0001
Repetitions vs Sequences Contrast	motor programming > motor control	Motor control relative to Motor programming	-.13	0.05	-.23	-0.03	-.16	-2.57	0.01
**Motor Dexterity -- Purdue Pegboard Task[13]**									
Average # pins placed - dominant hand	Better	Dexterity & Gross Motor	.01	.03	-.04	.06	.03	0.47	0.64
Average # pins placed - nondominant hand	Better	Dexterity & Gross Motor	-.007	.03	-.06	.04	-.02	-0.27	0.79
Average # pins picked – dominant hand	Better	Dexterity & Gross Motor	-.02	.02	-.05	.02	-.74	-1.11	0.27
Average # pins picked – nondominant hand	Better	Dexterity & Gross Motor	-.001	.01	-.03	.03	-.004	-0.05	0.96
Average # pins dropped – dominant hand	Worse	Dexterity	.01	.01	-.009	.03	.07	1.06	0.29
Average # pins dropped – nondominant hand	Worse	Dexterity	.02	.01	-.001	.04	.12	1.92	0.06
**Visuomotor Processing – NEPSY Design Copy[147]**									
Design Copying General - Total Score	Better	Visuo-construction Skills	.003	.04	-.08	.08	.005	0.07	0.94
Design Copying Process - Total Score	Better	Visuo-construction Skills	.11	.15	-.19	.41	.04	0.72	0.47
Design Copying Process Motor Score	Better	Fine Motor Control	.03	.06	-.08	.14	.03	0.52	0.61
Design Copying Global Score	Better	Gestalt	.03	.06	-.09	.16	.03	0.54	0.59
Design Copying Process Local Score	Better	Design Features	.02	.06	-.10	.15	.02	0.38	0.71
**Visuospatial Processing – NEPSY Geometric Puzzles[147]**									

© 2025 Peterson BS et al. *JAMA Neurology*.

Geometric Puzzles Total	Better	Visuospatial Perception	.11	0.05	0.005	.22	.13	2.07	0.04
**Visuospatial Processing - Water Level Task[148]**									
Water Jars correct	Better	Visuospatial Processing	-.02	.02	-.06	.02	-.06	-0.88	0.38
Water jars completed	Better	Visuospatial Processing Speed	.005	.003	-.001	.0	.10	1.56	0.12
**Psychomotor Fluency – NEPSY Visuomotor Precision Task[147]**									
Visuomotor Precision Combined	Better	Visuomotor Ability	.09	0.04	0.006	.18	.13	2.10	0.04
Visuomotor Precision Total Completion Time	Better	Visuomotor Performance Speed	-.11	0.05	-.20	-0.02	-.16	-2.51	0.01
**Attention -- NEPSY Auditory Attention[147]**									
Auditory Attention Combined	Better	Auditory Attention	-.05	.05	-.15	.05	-.07	-1.01	0.32
Auditory Attention Total Correct	Better	Auditory Attention	-.02	.03	-.09	.04	-.05	-0.70	0.56
Auditory Attention Total Commission Errors	Poorer	Auditory Attention	-.07	.05	-.17	.04	-.09	-1.30	0.19
Auditory Attention Total Omission Errors		Auditory Attention	.03	.03	-.04	.09	.05	0.79	0.54
Auditory Attention Total Inhibitory Errors	Worse	Auditory Attention	-.05	.05	-.14	.04	-.07	-1.06	0.29
**Cognitive Flexibility -- NEPSY Response Set[147]**									
Response Set Combined Scaled Score	Better	Selective and sustained attention	.08	.05	-.01	.17	.11	1.75	0.08
Response Set Total Correct	Better	Cognitive flexibility	.12	.06	-.008	.24	.12	1.84	0.07
Response Set Commission Errors	Better	auditory selective and sustained attention	-.09	.06	-.21	.03	-.10	-1.50	0.13
Response Set Total Inhibitory Errors	Worse	Impulsivity	-.11	.05	-.21	-.02	-.14	-2.27	0.02
Response Set Total Omission Errors	Worse	Inattention	-.11	.06	-.22	.008	-.12	-1.83	0.07
Auditory Attention vs Response Set Contrast	Better when cognitive demands high	Attention in context of high demand	.12	.05	.02	.22	.15	2.31	0.02
**Cognitive Flexibility -- NEPSY Inhibition Switching[147]**									
Inhibition (Condition 3) Switching Combined	Better	Attention Shifting	-.08	.05	-.18	.02	-.10	-1.53	0.13
Total Completion Time	Better	Impulsivity & Cognitive Flexibility	1.07	.62	-.16	2.30	.11	1.72	0.09
Total Errors	Worse	Attention Shifting	.18	.15	-.12	.47	.08	1.18	0.24
Total Uncorrected Errors	Worse	Attention Shifting	.12	.15	-.18	.42	.05	0.80	0.43
Total Self-Corrected Errors	Worse	Attention Shifting	.06	.06	-.06	.18	.06	1.00	0.32
Inhibition vs Switching Contrast	Better switching relative to level of inhibitory control	Attention Shifting	-.06	.05	-.15	.04	-.07	-1.09	0.27
**Naming Speed -- NEPSY Inhibition: Naming[147]**									

© 2025 Peterson BS et al. *JAMA Neurology*.

Naming (Condition 1) Combined Scaled Score	Better	Slow speed or poor accuracy	-.08	.06	-.20	.03	-.09	-1.45	0.15
Naming Total Completion Time	Better	Poor naming ability or slow processing speed	.18	.22	-.25	.60	.05	0.82	0.41
Naming Total Errors	Worse	Impulsivity	.07	.04	-.008	.14	.12	1.77	0.08
Naming Self-Corrected Errors	Worse	Impulsivity	.06	.03	.006	.11	.14	2.20	0.03
Naming Uncorrected Errors	Worse	Poor language or self-monitoring skills	.01	.03	-.04	.07	.02	0.37	0.71
**Resolution of Cognitive Conflict -- NEPSY Inhibition: Inhibition[147]**									
Inhibition (Condition 2) Combined Scaled Score	Better	Inhibitory Control & Cognitive Conflict Resolution	-.06	.06	-.17	.05	-.07	-1.08	0.28
Inhibition Total Completion Time	Better	Processing Speed	.48	.35	-.21	1.16	.09	1.37	0.17
Total Errors	Worse	Cognitive Conflict Resolution	.06	.09	-.12	.25	.04	0.65	0.52
Inhibition item 1: Shapes Total Errors	Worse	Cognitive Conflict Resolution	.25	.12	.01	.48	.14	2.08	0.04
Inhibition item 2: Arrows Total Errors	Worse	Cognitive Conflict Resolution	.05	.11	-.22	.31	.02	0.35	0.73
Total Uncorrected Errors	Worse	Cognitive Conflict Resolution	.004	.078	-.15	.16	.004	0.05	0.96
Total Self-Corrected Errors	Worse	Cognitive Conflict Resolution	.06	.047	-.04	.15	.08	1.19	0.23
Naming vs Inhibition Contrast	inhibition > naming speed	Inhibitory Control	-.03	.06	-.14	.09	-.03	-0.43	0.67
**Attention & Impulsivity -- Connors CPT-II T-Scores[14]**									
Overall Omission	Worse	Inattention	-.22	.31	-.83	.39	-.05	-0.72	0.48
Overall Commission	Worse	Impulsivity	.03	.15	-.27	.32	.01	0.19	0.85
Overall Hit Reaction Time	Worse	Response speed	-.36	.19	-.74	.02	-.12	-1.89	0.06
Overall Detectability	Worse	Discrimination of targets from non-targets	.03	.16	-.28	.33	.01	0.16	0.87
Overall Hit Standard Error Block Change	Worse	Sustained Attention: change in reaction time across the test duration	.25	.23	-.20	.70	.07	1.11	0.27
**Visual Reasoning NEPSY – Clocks[147]**									
Clocks Total Correct Score	Better	Visual reasoning	.06	.05	-.03	.15	.08	1.24	0.21
Number of Clocks Completed	Better	Visual reasoning	.10	.08	-.07	.27	.08	1.19	0.24
**Intelligence -- WISC-IV[15]**									
Full Scale IQ	Better	Full Scale IQ	-.15	.18	-.50	.20	-.05	-0.84	0.40
Verbal Comprehension	Better	Verbal IQ	-.05	.17	-.39	.29	-.02	-0.29	0.77
Perceptual Reasoning	Better	Performance IQ	-.12	.20	-.53	.28	-.04	-0.61	0.54
Working Memory	Better	Working Memory	-.22	.20	-.62	.18	-.07	-1.07	0.29
Processing Speed	Better	Processing Speed	-.04	.21	-.45	.36	-.01	-0.20	0.84

© 2025 Peterson BS et al. *JAMA Neurology*.

The dependent variable was test score, the independent variable prenatal was prenatal CPF exposure level, and covariates were Age, Sex, Maternal Ethnicity, Maternal Education, Material Hardship, and HOME score. The 2 CPF outlier values were excluded from analysis. P-values are uncorrected, but only those <0.005 were considered statistically significant after correction for multiple comparisons.

© 2025 Peterson BS et al. *JAMA Neurology*.

eTable 5: Associations of CPF Exposure with Symptom Severity Scores							
Test	Unstandardized Coefficient	Standardized Coefficient	95% Confidence Interval for B		Test Statistics		
	B	Std. Error	Lower Bound	Upper Bound	Beta	t	p-value
**Child Behavioral Checklist (CBCL)[149,150] – T-Scores**							
Total Score	-.20	.18	-.55	.16	-.07	-1.09	0.28
Anxious/Depressed	-.06	.09	-.23	.11	-.04	-0.68	0.50
Withdrawn	-.10	.10	-.30	.10	-.06	-0.98	0.33
Somatic Complaints	-.16	.11	-.37	.05	-.09	-1.52	0.13
Social Problems	-.01	.08	-.17	.14	-.01	-0.18	0.86
Thought Problems	-.07	.08	-.22	.08	-.06	-0.89	0.38
Attention Problems	-.09	.10	-.28	.10	-.06	-0.94	0.35
Rule-Breaking Behaviors	-.05	.08	-.21	.11	-.04	-0.60	0.55
Aggressive Behaviors	-.12	.09	-.28	.05	-.09	-1.34	0.18
Internalizing Problems	-.21	.17	-.55	.13	-.08	-1.21	0.23
Externalizing Problems	-.22	.15	-.52	.09	-.09	-1.41	0.16
DSM Affective Problems	-.09	.10	-.28	.11	-.05	-0.86	0.39
DSM Anxiety Problems	-.06	.09	-.24	.13	-.04	-0.62	0.54
DSM Somatic Problems	-.12	.11	-.34	.11	-.07	-1.04	0.30
DSM ADHD Problems	-.07	.08	-.22	.09	-.06	-0.86	0.39
DSM Oppositional Defiant Problems	-.14	.08	-.30	.02	-.11	-1.75	0.08
DSM Conduct Problems	-.06	.08	-.23	.10	-.05	-0.74	0.46
**Children's Depression Rating Scale[17]**							
Total Depression Severity	-.29	.12	-.52	-0.05	-.16	-2.42	0.02
**Revised Children's Manifest Anxiety Scale[151]**							
Total Anxiety Severity	-.01	.10	-.20	-.18	-.007	-0.11	0.92
Physiological Anxiety	-.002	.04	-.08	.07	-.004	-0.06	0.95
Worry/Oversensitivity	-.03	.05	-.12	.06	-.04	-0.66	0.51
Social Anxiety	.02	.03	-.04	.08	.05	0.72	0.47
Lie Subscale	.001	.04	-.08	.08	.001	-0.02	0.98
**ADHD Rating Scale[152]**							
Total Score - current	-.10	.15	-.39	.19	-.04	-0.67	0.50
Inattention Score - current	-.08	.08	-.25	.08	-.06	-0.99	0.32
Hyperactivity/Impulsivity Score - current	-.02	.07	-.16	.13	-.01	-0.22	0.82
Total Score - worst ever	-.37	.20	-.76	.03	-.12	-1.84	0.07
Inattention Score - worst ever	-.17	.11	-.38	.03	-.11	-1.64	0.10
Hyperactivity/Impulsivity Score - worst ever	-.19	.10	-.39	.009	-.12	-1.88	0.06
**Social Responsiveness Scale[153,154] – T-Scores**							
Total Score	-.007	.15	-.31	.30	-.003	-0.05	0.96
Awareness	-.09	.16	-.41	.23	-.04	-0.55	0.58
Cognition	.02	.16	-.30	.34	.008	0.12	0.90
Communication	-.01	.16	-.32	.30	-.006	-0.09	0.93
Motivation	-.04	.16	-.36	.29	-.01	-0.21	0.83
Mannerisms	.07	.17	-.27	.10	.03	0.39	0.70

Symptom score was the dependent variable, prenatal CPF level was the independent variable, and covariates were Age, Sex, Maternal Ethnicity, Maternal Education, Material Hardship, and HOME score. The 2 CPF outlier values were excluded from analysis. P-values are uncorrected, but only those <0.005 were considered statistically significant after correction for multiple comparisons.

© 2025 Peterson BS et al. *JAMA Neurology*.

eTable 6: Association of CPF Exposure Levels with DTI and rs-fMRI Graph Theoretical Measures

Variables (Average Thresholds)	Unstandardized Coefficient	Standardized Coefficient	95.0% Confidence Interval for B		Test Statistics		
	B	Std. Error	Lower Bound	Upper Bound	Beta	t	p-value
**DTI Graph Theoretical Measures**							
Density	-4.945E-5	.001	-.002	.002	-.004	-.06	0.95
Average Clustering Coefficient	.0001	.0001	.0001	.001	.06	.84	0.40
Local Efficiency	.03	.03	-.03	.10	.07	1.02	0.31
Characteristic Path Length	-.001	.002	-.005	.003	-.03	-.45	0.66
Small Worldness	.0001	.001	-.001	.001	.02	.33	0.74
Diameter	.003	.006	-.008	.015	.04	.57	0.57
Rich Club k 25	.0001	.001	-.001	.001	.01	.15	0.88
**rs-fMRI Graph Theoretical Measures**							
Community Structure	.001	.002	-.003	.005	.05	.56	0.58
Density	-.002	.002	-.007	.002	-.08	-.96	0.34
Global Efficiency	-.002	.002	-.006	.002	-.08	-1.00	0.32
Diameter	.01	.01	-.01	.04	.09	1.09	0.27
Radius	.001	.004	-.007	.009	.02	.30	0.77
Characteristic Path Length	.004	.005	-.006	.01	.06	.77	0.45
Transitivity	-.002	.002	-.005	.002	-.07	-.90	0.37

Each GT measure was entered separately into a linear regression model as the dependent variable, CPF level was the independent variable, and covariates were participant age at MRI scan, sex, ethnicity, maternal education, material hardship during pregnancy, and home stress at child age 3 years.

© 2025 Peterson BS et al. *JAMA Neurology*.

SUPPLEMENTAL FIGURES

© 2025 Peterson BS et al. *JAMA Neurology*.

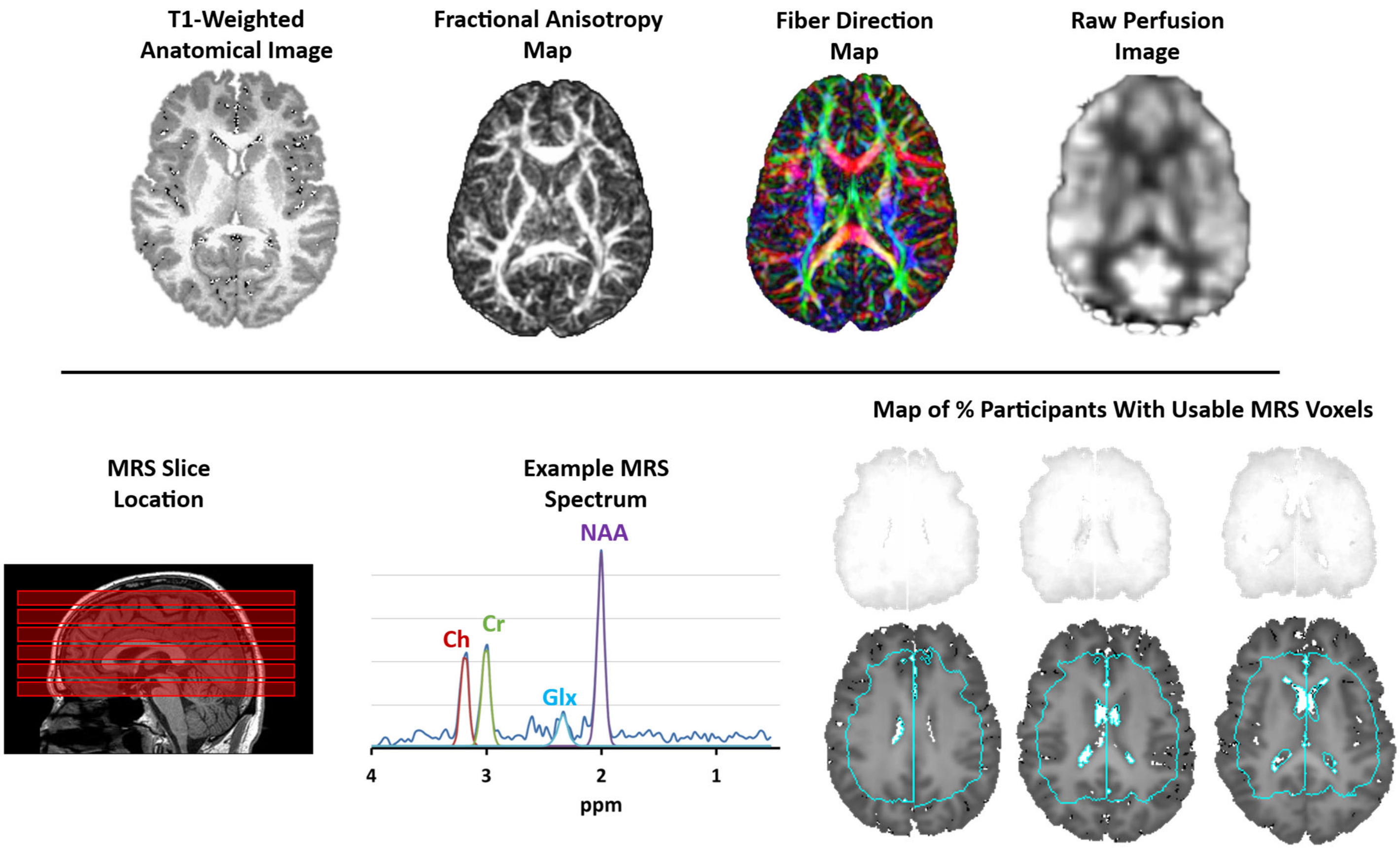

eFigure 1: Image Quality in Each MRI Modality

Upper Row: Anatomical The left-most figure shows the excellent contrast and high signal to noise in a T1-weighted anatomical image. DTI The middle 2 images show, an FA Map in a single participant and a tensor direction map for the same participant in which the principal direction of each estimated tensor is color-coded: red=left-right; green=up-down; blue=perpendicular to the 2D slice. ASL The right-most figure shows a perfusion image, with the gray scale encoding rCBF values.

Lower Row: MPSI Left-most image shows the 6 transaxial slice locations for data acquisition that are prescribed parallel to the AC-PC line in a mid-sagittal image. The second figure shows an example of a spectrum from a single MPCSI voxel in the superior parietal cortex of one participant after signals were combined across all 8 channels. The NAA, Ch, Cr, and Glx peaks are labeled and color-coded. The SNR of the combined spectrum from the 8-channel coil is approximately twice that of the SNR of spectra from a standard quadrature coil. The right-most images show, in the top row, gray scale images representing the number of participants who had usable MRS data at the location shown in that transaxial slice. In the lower row are the boundaries on 3 transaxial slices outside which fewer than 70% of participants had usable voxels retained outside the contour. Loss of usable voxels at the periphery of the brain was caused by the saturation bands used to suppress lipid signal from the scalp, which were not as precisely shaped as the brain. Therefore, the saturation bands unavoidably suppressed metabolite signals from a substantial portion of cortical gray matter in each participant.

© 2025 Peterson BS et al. *JAMA Neurology*.

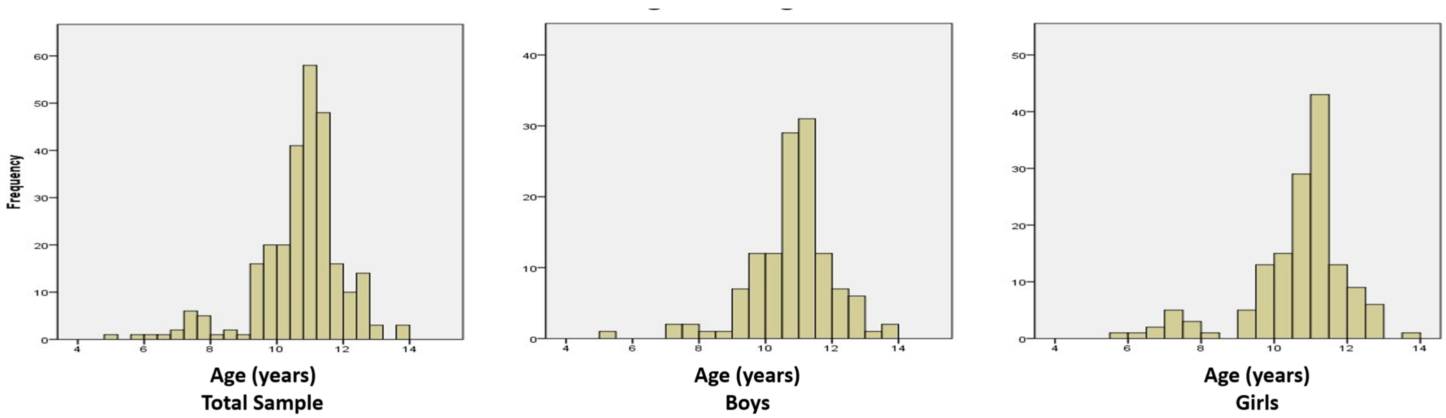

eFigure 2: Age Distributions by Participant Sex
Shown here are the histograms for participant ages in the entire sample of N=270 and for the boys and girls separately.

© 2025 Peterson BS et al. *JAMA Neurology*.

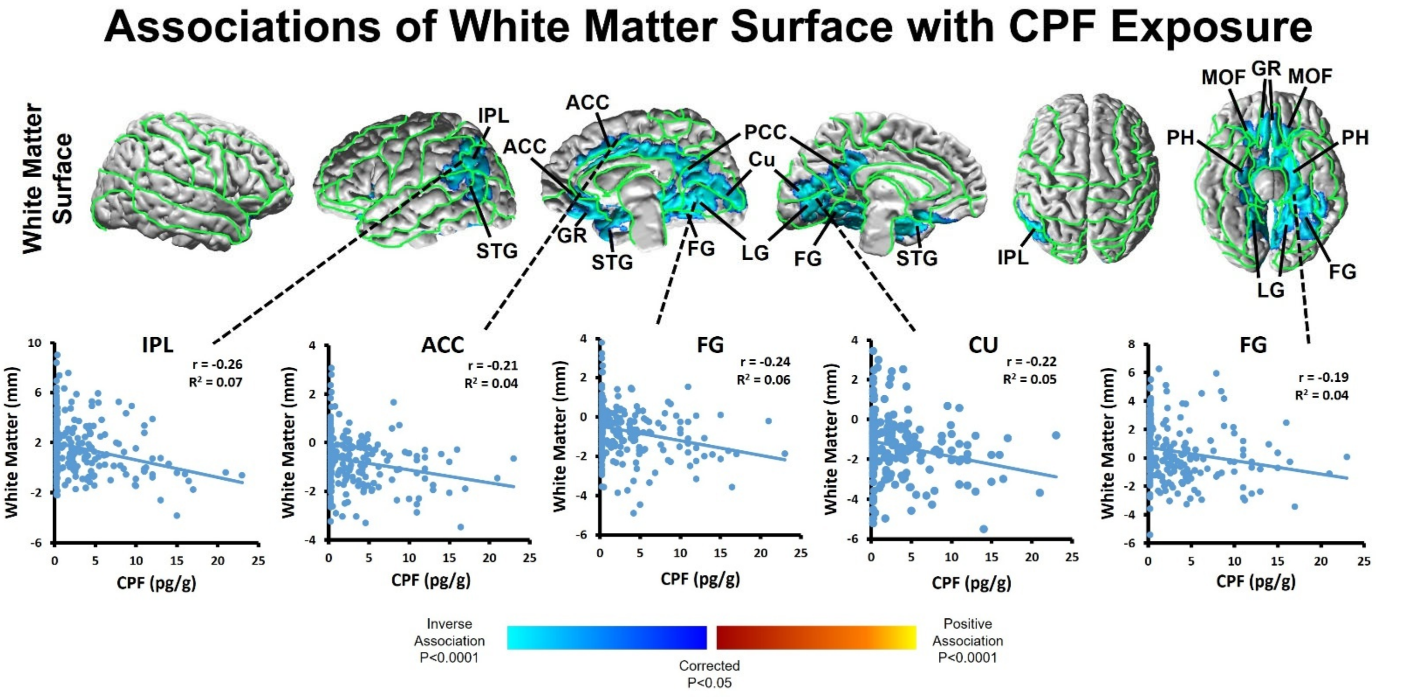

eFigure 3: Statistical Maps of Prenatal CPF Exposure Associations with White Matter Surface A regression model tested exposure effects at each point on the white matter surface: *Imaging Measure* = $\beta_0 + \beta_1 * CPF + \beta_2 * Age + \beta_3 * Sex + \beta_4 * Ethnicity + \beta_5 * Maternal\ Education + \beta_6 * Material\ Hardship + \beta_7 * Home\ Stress + \varepsilon$, with "imaging measure" being signed Euclidean distance ("local volume") of the white matter surface from the surface of a template brain. The number of participants for this analysis was 262 (Mean Age: 10.75; boys = 120; girls = 142). FWER-correction and color coding is the same as for main text Figure 2.. Only p-values that survived cluster size FWER correction are plotted, with color-coding of the p-value as shown in the color bar. Views of the brain, shown left to right, are right lateral, left lateral, right mesial, left mesial, dorsal, and ventral.

Anatomical MRI measures were sampled at representative points as indicated, and scatterplots for the association of CPF exposure with those measures are shown for each sampled point. White matter measures were adjusted for participant age at MRI scan, sex, ethnicity, maternal education, material hardship during pregnancy, and home stress at child age 3 years.

Abbreviations: ACC: anterior cingulate cortex; **Cu:** cuneus; **FG:** fusiform gyrus; **GR:** gyrus rectus; **IFG:** inferior frontal gyrus; **IOG:** inferior occipital gyrus; **IPL:** inferior parietal lobule; **ITG:** inferior temporal gyrus; **LOF:** lateral orbitofrontal gyrus; **LG:** lingual gyrus; **MFG:** middle frontal gyrus; **MOF:** middle orbitofrontal gyrus; **MTG:** middle temporal gyrus; **PCC:** posterior cingulate cortex; **SFG:** superior frontal gyrus; **SPG:** superior parietal gyrus; **STG:** superior temporal gyrus

© 2025 Peterson BS et al. *JAMA Neurology*.

FA

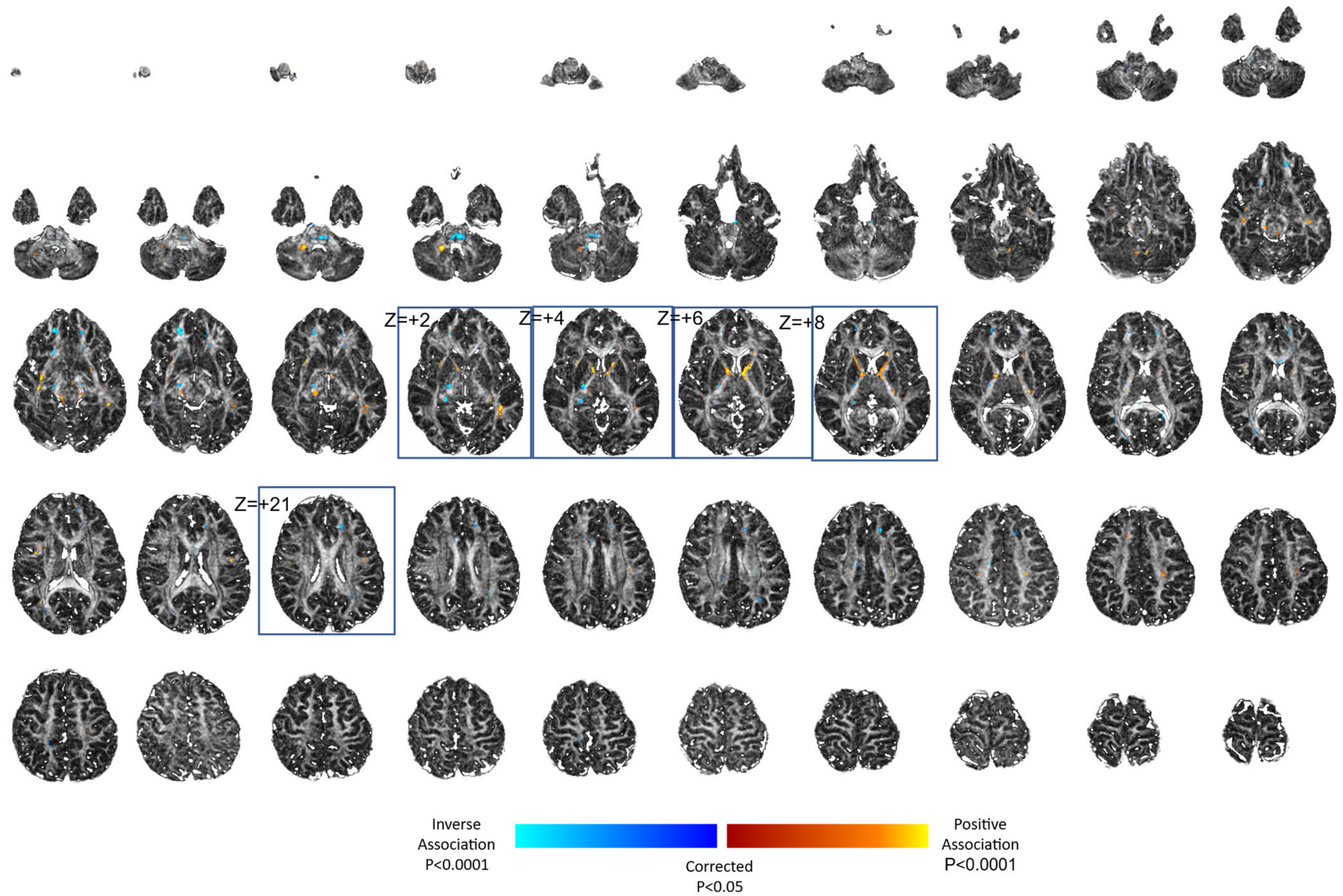

eFigure 4: CPF Exposure Associations with FA Values -- All Slices for Tissue-Specific White Matter Maps Slices selected to represent findings in the main text are shown in boxes. The regression model testing exposure associations at each voxel was FA= β_0 + $\underline{\beta_1 * CPF}$ + β_2*Age* + β_3*Sex* + β_4*Ethnicity* + β_5*Maternal Education* + β_6*Material Hardship* + β_7*Home Stress* + ε, This model controls for participant age at MRI scan, sex, and ethnicity, maternal education material hardship during pregnancy, and home stress at child age 3 years. P-values for β_1 that survived the procedure for False Discovery Rate at an FDR = 0.05 were color-coded as shown in the color bars and then displayed on the template brain. Transaxial slices are positioned parallel to the anterior commissure–posterior commissure line, with Z-levels shown from the Talairach coordinate system. The number of participants was 202 (Mean Age: 10.78; boys = 89; girls = 113).

.

© 2025 Peterson BS et al. *JAMA Neurology*.

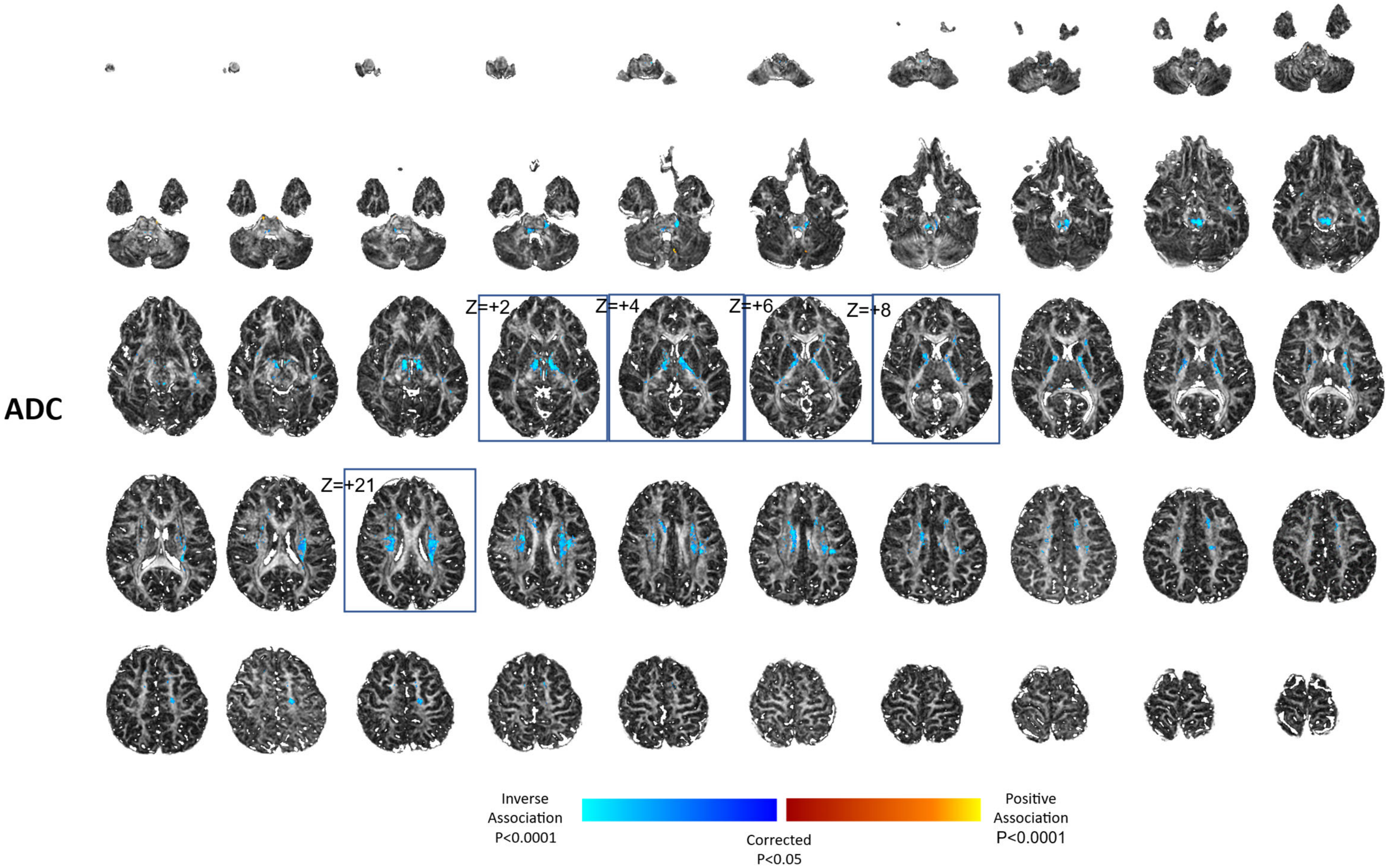

eFigure 5: CPF Exposure Associations with ADC Values -- All Slices for Tissue-Specific White Matter Maps Slices selected to represent findings in the main text are shown in boxes. The regression model testing exposure associations at each voxel was ADC= β_0 + β_1*CPF + β_2*Age + β_3*Sex + β_4*Ethnicity + β_5*Maternal Education + β_6*Material Hardship + β_7*Home Stress + ε, This model controls for participant age at MRI scan, sex, and ethnicity, maternal education material hardship during pregnancy, and home stress at child age 3 years. P-values for β_1 that survived the procedure for False Discovery Rate at an FDR = 0.05 were color-coded as shown in the color bars and then displayed on the template brain. Transaxial slices are positioned parallel to the anterior commissure–posterior commissure line, with Z-levels shown from the Talairach coordinate system. The number of participants was 202 (Mean Age: 10.78; boys = 89; girls = 113).

© 2025 Peterson BS et al. *JAMA Neurology*.

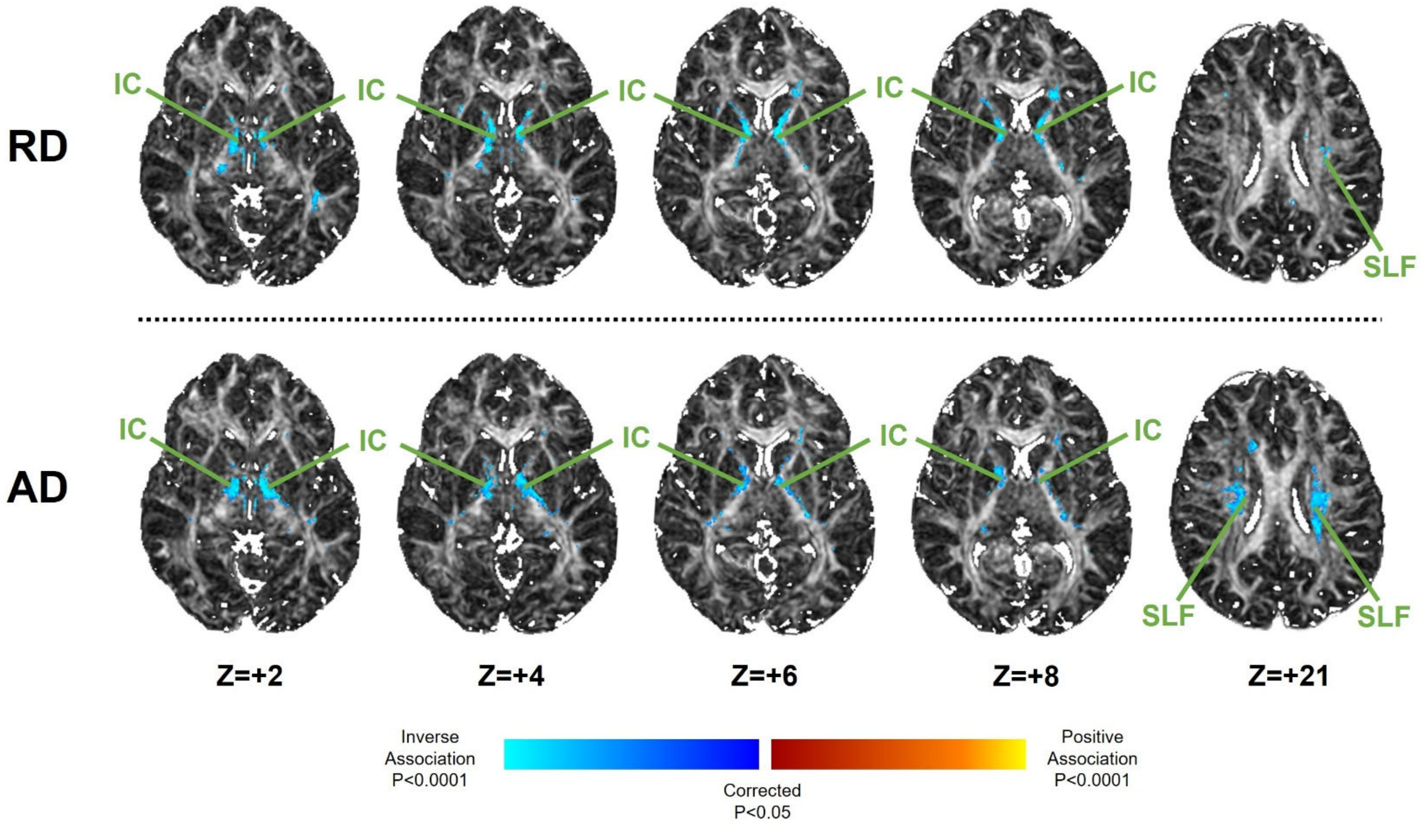

eFigure 6: Statistical Maps of CPF Prenatal Exposure Effects on Axial and Radial Diffusivity in White Matter
The regression model that tested CPF exposure effects at each white matter voxel was $DTI\ Measure = \beta_0 + \beta_1{*}CPF + \beta_2{*}Age + \beta_3{*}Sex + \beta_4{*}Ethnicity + \beta_5{*}Maternal\ Education + \beta_6{*}Material\ Hardship + \beta_7{*}Home\ Stress + \varepsilon$, with "DTI Measure" either radial diffusivity (RD) or axial diffusivity (AD). FWER-correction and color coding is identical to main text Figure 2. The number of participants in this analysis was 202 (Mean Age: 10.78; boys = 89; girls = 113). The Z-values below each column represent the Z-coordinate in Talairach space.

FA and ADC values were sampled at representative points, and scatterplots for the association of CPF exposure with the DTI values at those points are shown. DTI values are adjusted for participant age at MRI scan, sex, ethnicity, maternal education, material hardship during pregnancy, and home stress at child age 3.

Abbreviations: IC: internal capsule; **ILF:** inferior longitudinal fasciculus; **SLF:** superior longitudinal fasciculus

© 2025 Peterson BS et al. *JAMA Neurology*.

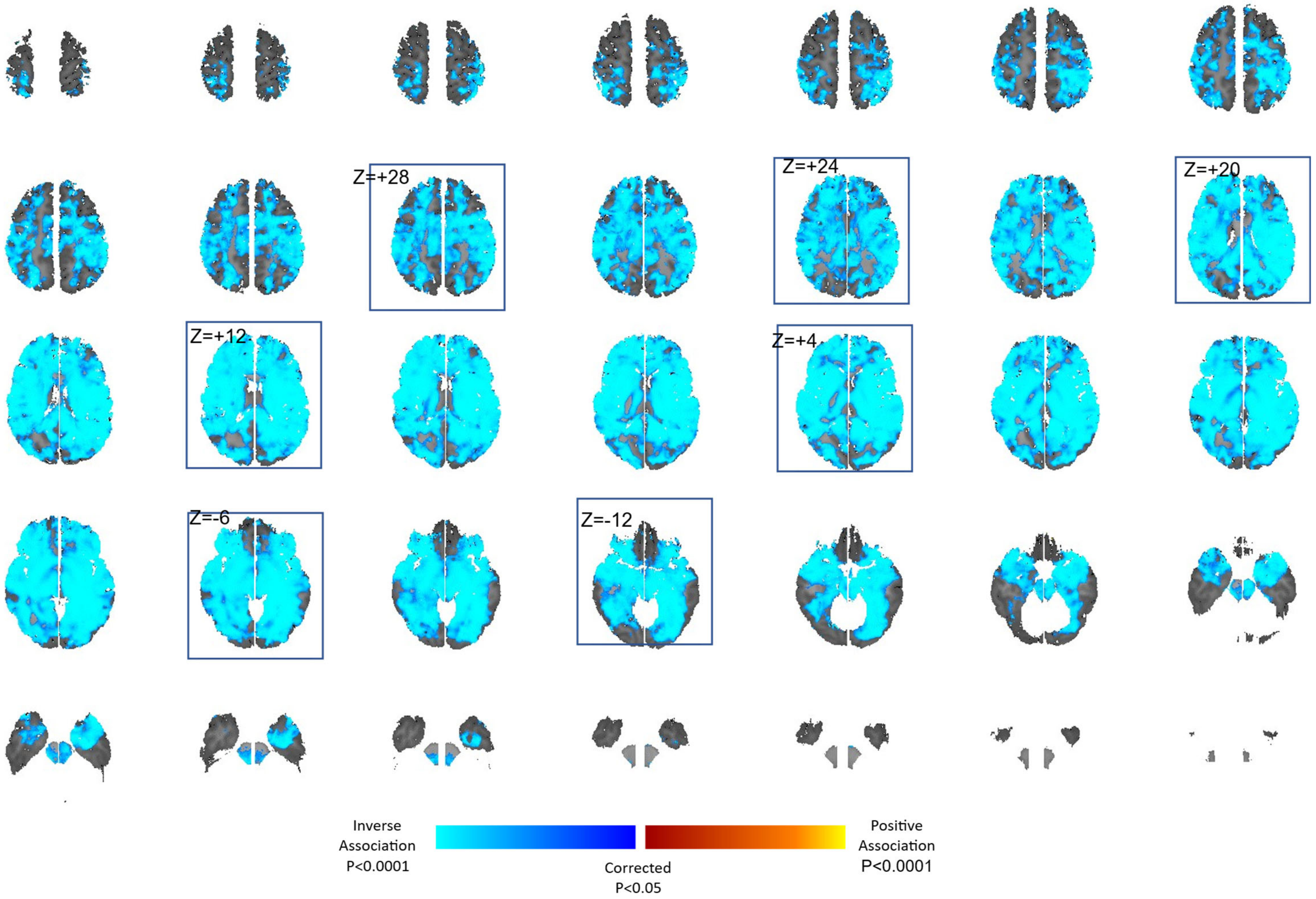

eFigure 7: All Slices for CPF Exposure Associations with rCBF The slices selected to represent findings in the main text are shown in boxes. The regression model was *rCBF values*= β_0 + β_1**CPF* + β_2**Age* + β_3**Sex* + β_4**Ethnicity* + β_5**Maternal Education* + β_6**Material Hardship* + β_7**Home Stress* + ε. This model controls for participant age at MRI scan, sex, ethnicity, maternal education, material hardship during pregnancy, and home stress at child age 3 years. P-values for β_1 that survived the procedure for False Discovery Rate at an FDR = 0.05 were color-coded as shown in the color bars and then displayed on the template brain. Transaxial slices are positioned parallel to the anterior commissure–posterior commissure line, with Z-levels shown from the Talairach coordinate system. The number of participants was 175 (Mean Age: 10.89; Males = 75; Females = 100).

© 2025 Peterson BS et al. *JAMA Neurology*.

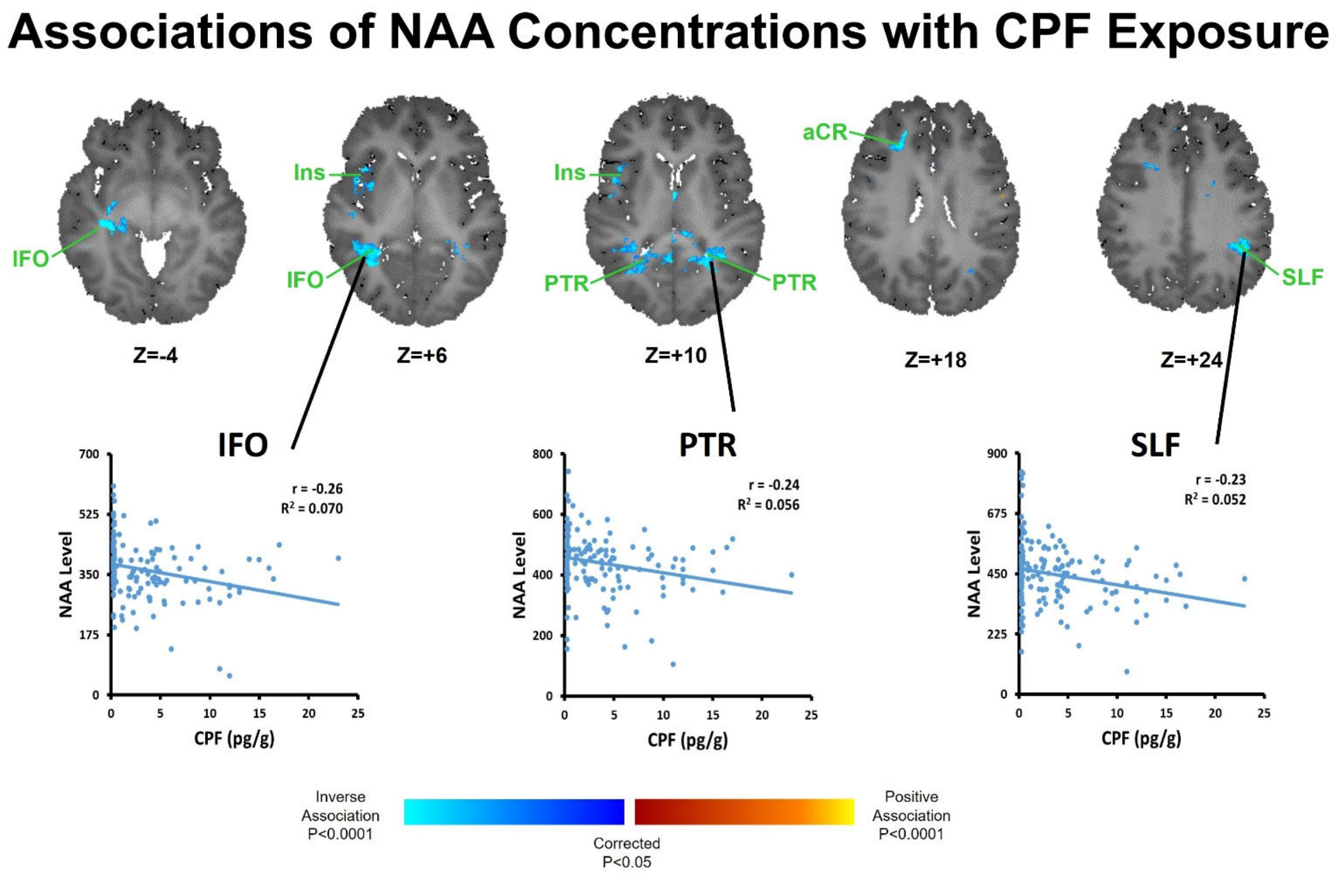

eFigure 8: Statistical Map of Prenatal CPF Exposure Associations with NAA Levels
The regression model that tested CPF exposure effects voxelwise was $NAA = \beta_0 + \beta_1 * CPF + \beta_2 * Age + \beta_3 * Sex + \beta_4 * Ethnicity + \beta_5 * Maternal\ Education + \beta_6 * Material\ Hardship + \beta_7 * Home\ Stress + \varepsilon$. FWER-correction and color coding is identical to Figure 2 in the main text. The number of participants was 211 (Mean Age: 10.78; boys = 98; girls = 113). The Z-values below each column represent the Z-coordinate in Talairach space.

NAA data were sampled at representative points. Scatterplots showing the association of CPF exposure with NAA concentrations are shown for those points. NAA values are adjusted for participant age at MRI scan, sex, ethnicity, maternal education, material hardship during pregnancy, and home stress at child age 3 years.

Abbreviations: aCR: anterior corona radiata; **IFO:** inferior fronto-occipital fasciculus; **Ins:** insula; **PTR:** posterior thalamic radiation; **SLF:** superior longitudinal fasciculus

© 2025 Peterson BS et al. *JAMA Neurology*.

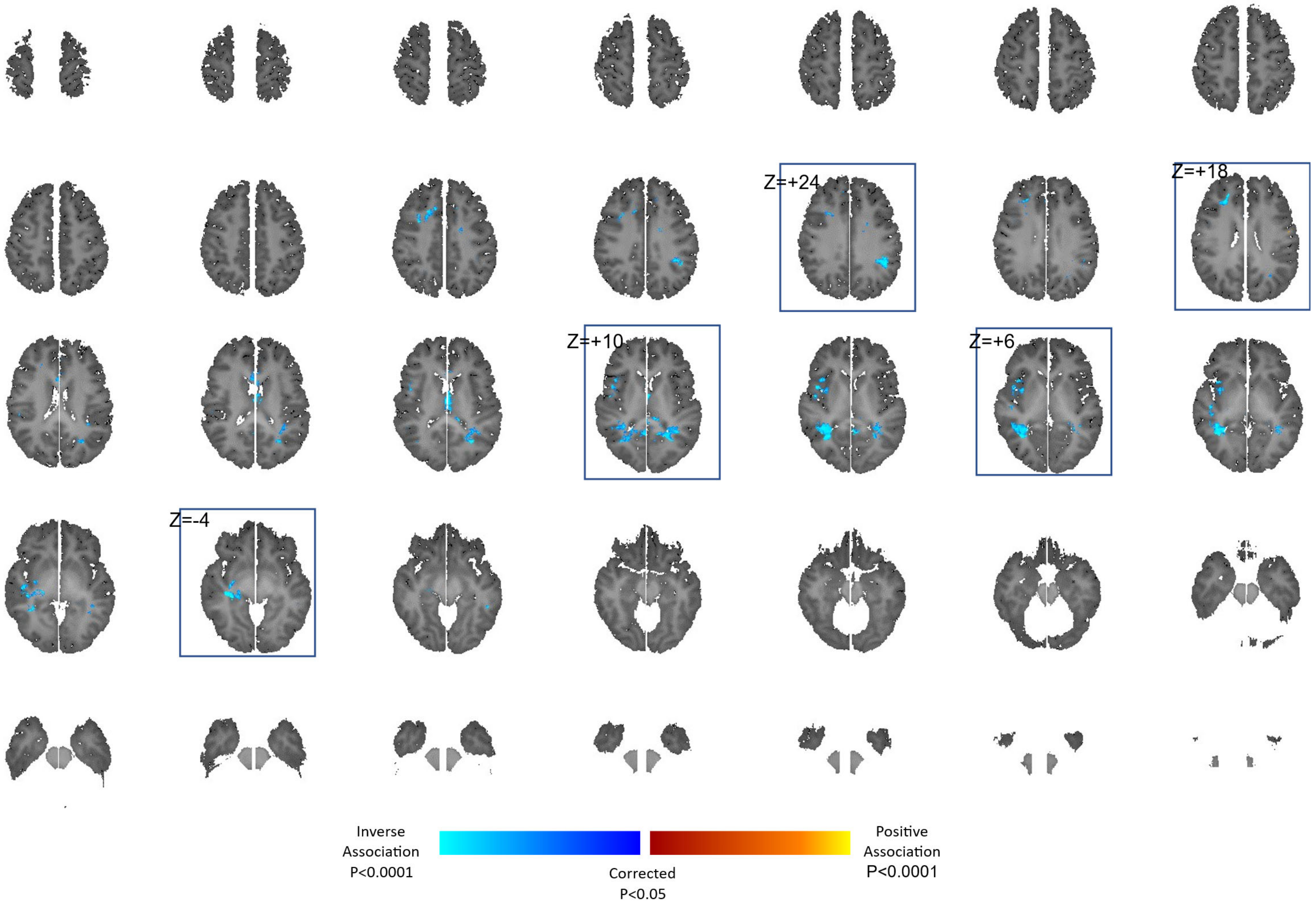

eFigure 9: All Slices for CPF Exposure Associations with NAA Levels The slices selected to represent findings in the main text are shown in boxes. The regression model was *NAA values*= $\beta_0 + \beta_1*CPF + \beta_2*Age + \beta_3*Sex + \beta_4*Ethnicity + \beta_5*Maternal\ Education + \beta_6*Material\ Hardship + \beta_7*Home\ Stress + \varepsilon$. This model controls for participant age at MRI scan, sex, ethnicity, maternal education, material hardship during pregnancy, and home stress at child age 3 years. P-values for β_1 that survived the procedure for False Discovery Rate at an FDR = 0.05 were color-coded as shown in the color bars and then displayed on the template brain. Transaxial slices are positioned parallel to the anterior commissure–posterior commissure line, with Z-levels shown from the Talairach coordinate system. The number of participants was 211 (Mean Age: 10.78; boys = 98; girls = 113).

© 2025 Peterson BS et al. *JAMA Neurology*.

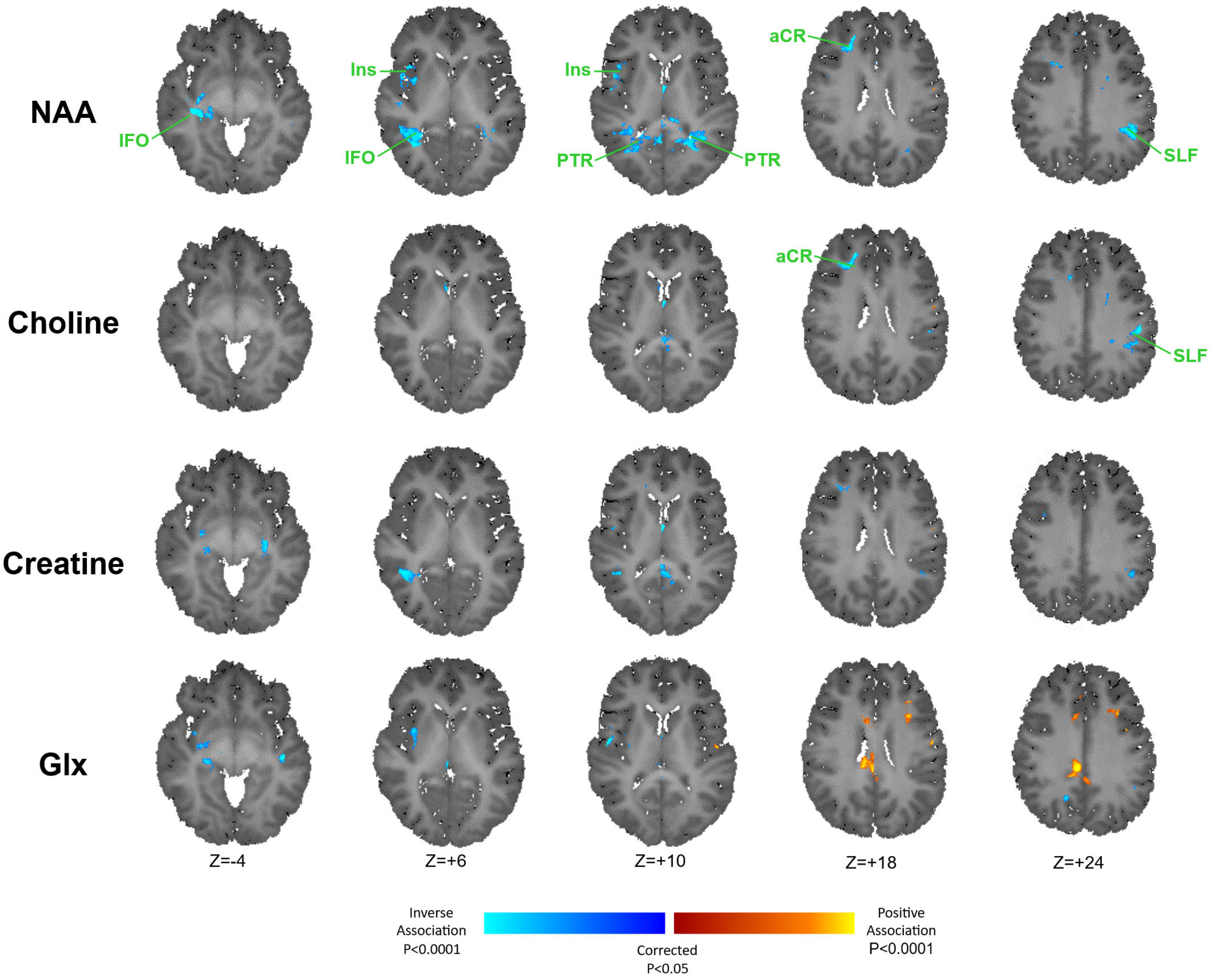

eFigure 10: Association of Prenatal CPF Levels with All Metabolite Concentrations Shown here are the associations of CPF levels with concentrations for each metabolite measured – NAA, Choline, Creatine, and Glx. The regression model was *MRS Measure* = β_0 + β_1*CPF_+ β_2*Age + β_3*Sex + β_4*Ethnicity + β_5*Maternal Education + β_6*Material Hardship + β_7*Home Stress + ε, where “MRS Measure” was either NAA, Choline, Creatine or Glx concentration. This model controls for participant age at MRI scan, sex, ethnicity, maternal education, material hardship during pregnancy, and home stress at child age 3 years. P-values for β_1 that survived the procedure for False Discovery Rate at an FDR = 0.05 were color-coded as shown in the color bars and then displayed on the template brain. Transaxial slices are positioned parallel to the anterior commissure–posterior commissure line, with Z-levels shown from the Talairach coordinate system. The number of participants was 211 (Mean Age: 10.78; boys = 98; girls = 113).

Abbreviations: aCR: anterior corona radiata; **IFO:** inferior fronto-occipital fasciculus; **Ins:** insula; **PTR:** posterior thalamic radiation; **SLF:** superior longitudinal fasciculus.

© 2025 Peterson BS et al. *JAMA Neurology*.

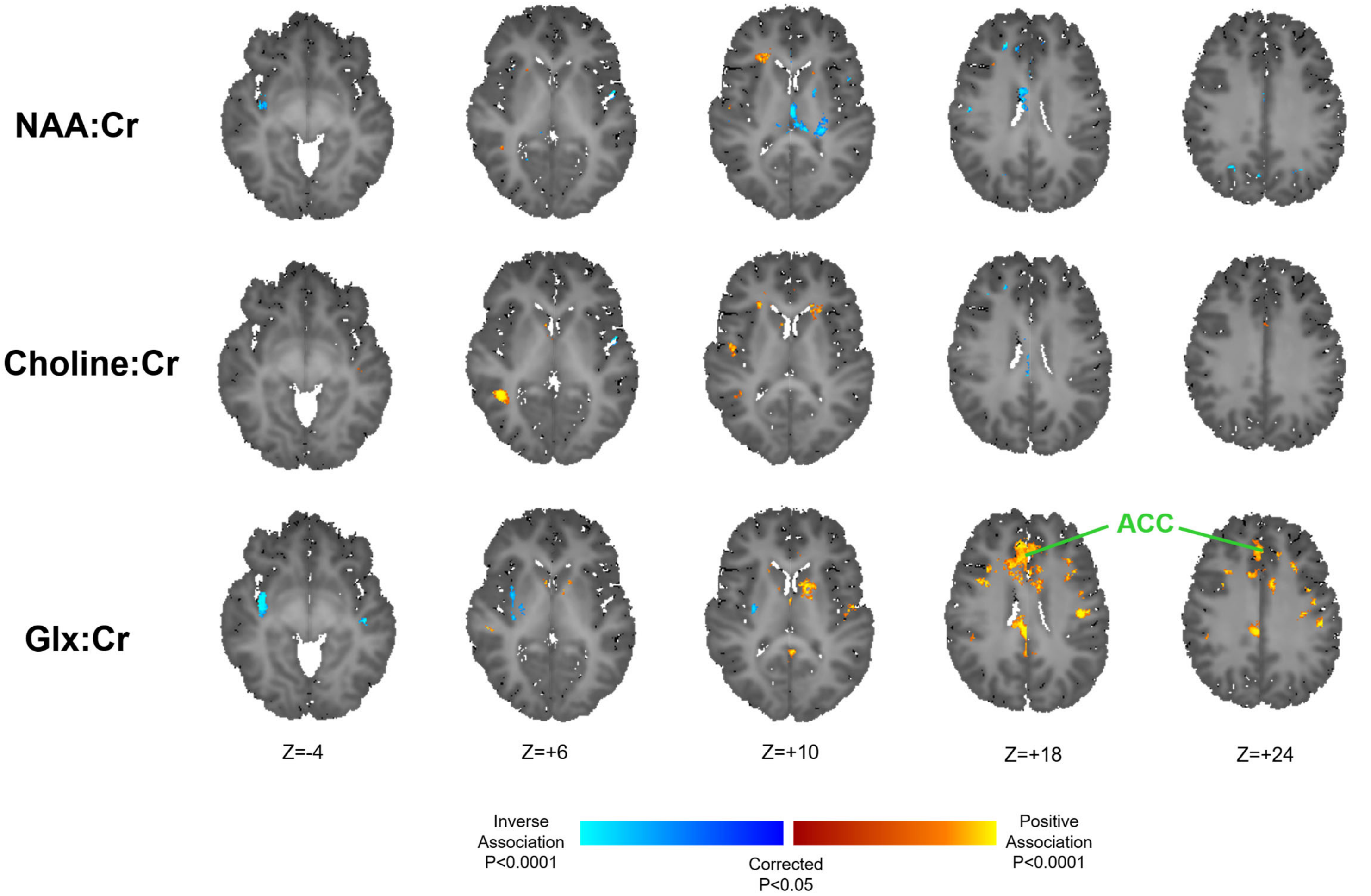

eFigure 11: Associations of CPF Exposure with Metabolite Concentrations Normalized to Creatine Shown here are the associations of CPF levels with concentrations for NAA, Choline, and Glx, each normalized to Creatine Levels. The regression model was *MRS Measure* = β_0 + β_1**CPF_*+ β_2**Age* + β_3**Sex* + β_4**Ethnicity* + β_5**Maternal Education* + β_6**Material Hardship* + β_7**Home Stress* + ε, where "MRS Measure" was either NAA:Cr, Choline:Cr, or Glx"Cr concentration. This model controls for participant age at MRI scan, sex, ethnicity, maternal education, material hardship during pregnancy, and home stress at child age 3 years. P-values for β_1 that survived the procedure for False Discovery Rate at an FDR = 0.05 were color-coded as shown in the color bars and then displayed on the template brain. Transaxial slices are positioned parallel to the anterior commissure–posterior commissure line, with Z-levels shown from the Talairach coordinate system. The number of participants was 211 (Mean Age: 10.78; boys = 98; girls = 113).
Abbreviations: ACC: anterior cingulate cortex

© 2025 Peterson BS et al. *JAMA Neurology*.

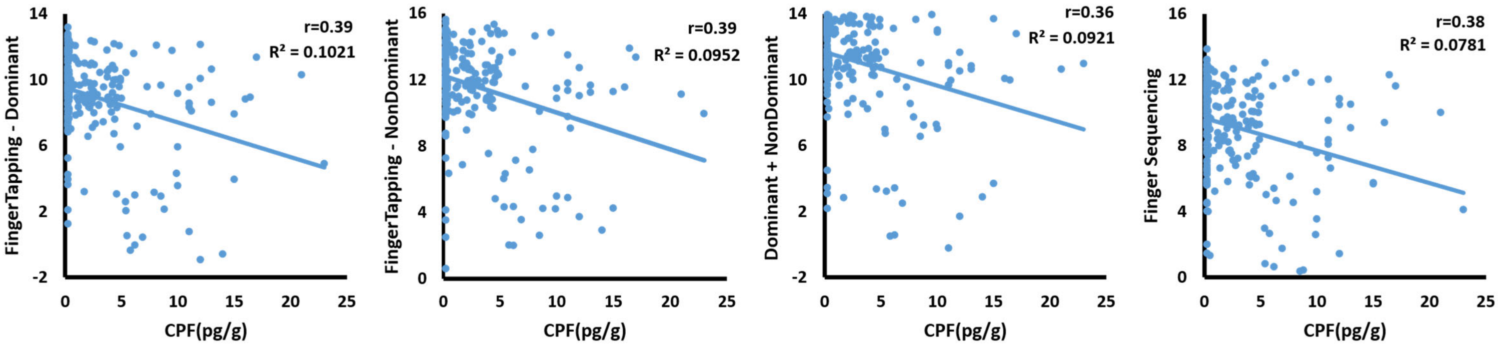

eFigure 12: Scatterplot for FingerTapping Task Shown here are the scatterplots for the associations of prenatal CPF exposures with fingertapping scores on the NEPSY-II (eTable 4) for speed of the dominant hand, nondominant hand, both hands combined, and the sequencing task. The scores are adjusted for participant age at MRI scan, sex, ethnicity, maternal education, material hardship during pregnancy, and home stress at child age 3 years.

© 2025 Peterson BS et al. *JAMA Neurology*.

Sensitivity Analysis
Air Pollution: Covarying for Prenatal PAH and PM_{25}

© 2025 Peterson BS et al. *JAMA Neurology*.

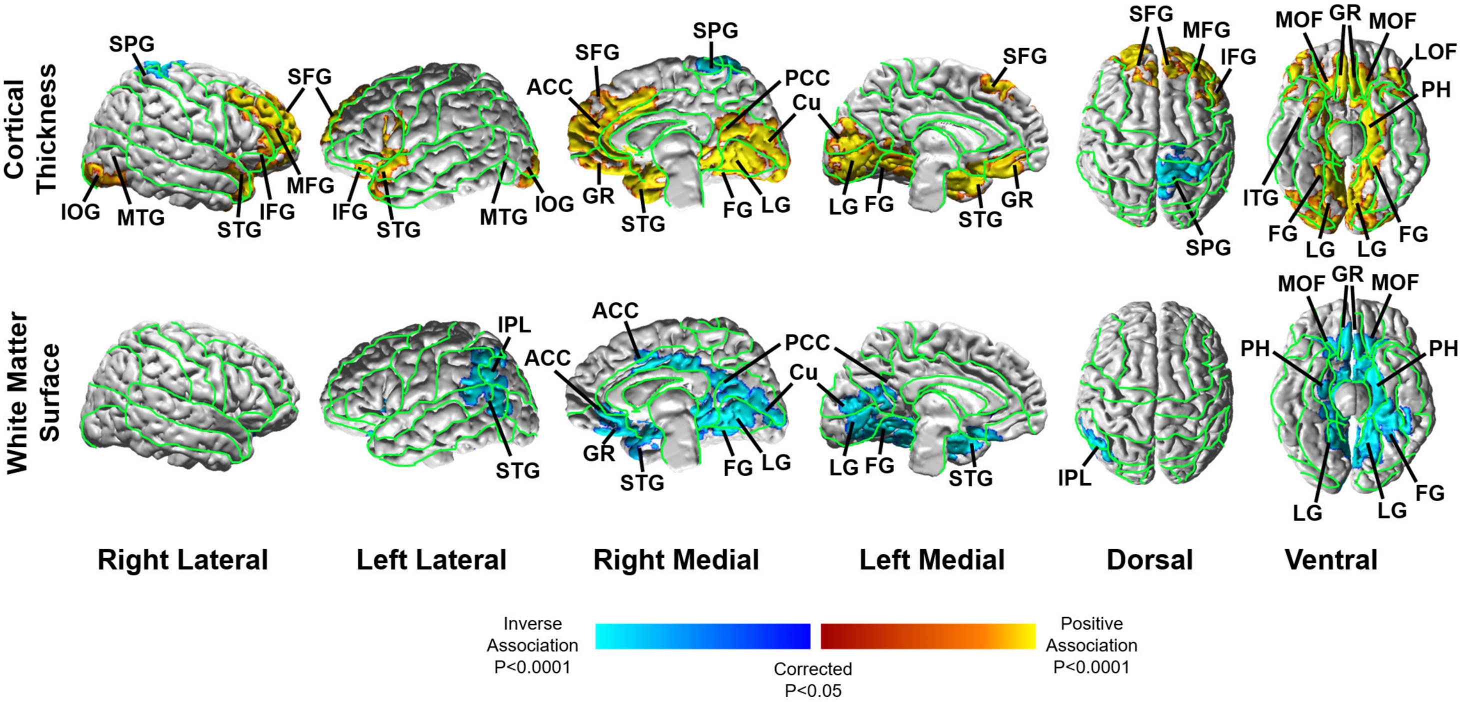

eFigure 13: CPF Exposure Associations with Anatomical Measures While Covarying for Prenatal PAH and PM_{25} Exposures The regression model was *Imaging Measure* = $\beta_0 + \beta_1$*CPF + β_2*Age + β_3*Sex + β_4*Ethnicity + β_5*Maternal Education + β_6*Material Hardship + β_7*Home Stress +β_8*PAH + β_9*PM_{25} +ε. This model controls for participant age at MRI scan, sex, ethnicity, maternal education, material hardship during pregnancy, home stress at child age 3 years, prenatal PAH exposure, and prenatal PM25 exposure. P-values for β_1 that survived the procedure for False Discovery Rate at an FDR = 0.05 were color-coded as shown in the color bars and then displayed on the template brain. The 2 outliers were excluded, and not all participants had PAH or $PM_{2.5}$ exposure estimates.The number of participants was 249 (Mean Age: 10.76; boys = 114; girls = 135).

Abbreviation: ACC, anterior cingulate cortex; **Cu,** Cuneus; **dACC,** dorsal anterior cingulate cortex; **FG,** fusiform gyrus; **GR,** gyrus rectus; **IFG,** inferior frontal gyrus; **IOG,** inferior occipital gyrus; IPL, inferior parietal lobule; **ITG,** inferior temporal gyrus; **LG,** lingual gyrus; **MFG,** middle frontal gyrus; **MOF,** middle orbitofrontal gyrus; **MTG,** middle temporal gyrus; **PCC,** posterior cingulate cortex; **PrPo,** pre/postcentral gyrus; **SFG,** superior frontal gyrus; **SPG,** superior parietal gyrus; **STG**, superior temporal gyrus; **vACC,** ventral anterior cingulate cortex.

© 2025 Peterson BS et al. *JAMA Neurology*.

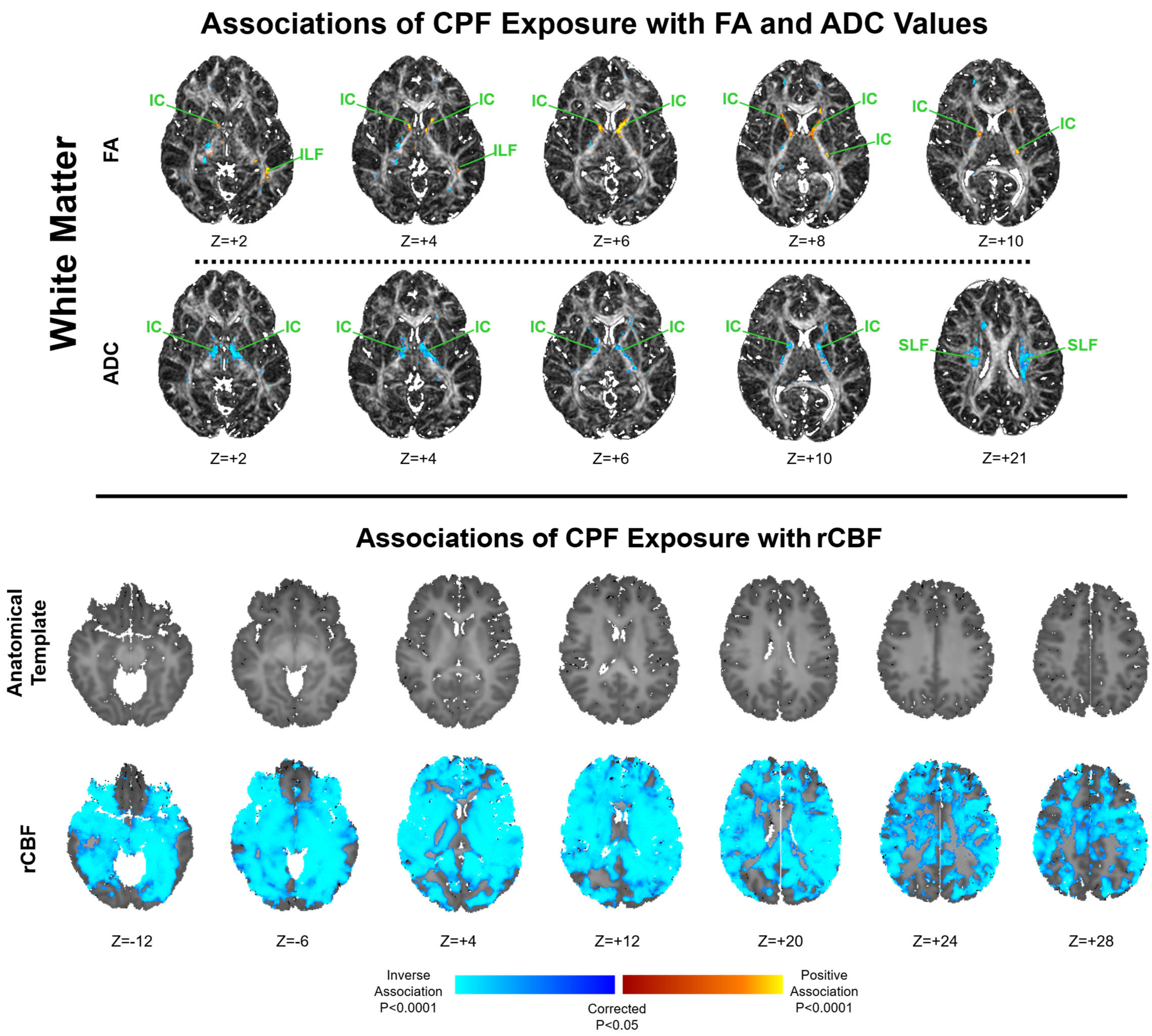

eFigure 14: CPF Exposure Associations with DTI and rCBF Measures While Covarying for Prenatal PAH and PM25 Exposures The regression model was *Imaging Measure* = β_0 + β_1*CPF + β_2*Age + β_3*Sex + β_4*Ethnicity + β_5*Maternal Education + β_6*Material Hardship + β_7*Home Stress +β_8*PAH + β_9*PM_{25} +ε. The dependent Imaging Measure was either FA, ADC, or rCBF. This model controls for participant age at MRI scan, sex, ethnicity, maternal education, material hardship during pregnancy, home stress at child age 3 years, prenatal PAH exposure, and prenatal PM25 exposure. P-values for β_1 that survived the procedure for False Discovery Rate at an FDR = 0.05 were color-coded as shown in the color bars and then displayed on the template brain. The 2 outliers were excluded, and not all participants had PAH or $PM_{2.5}$ exposure estimates.The number of participants for DTI was 192 (Mean Age: 10.78; boys = 84; girls = 108) and for rCBF was 165 (Mean Age: 10.88; boys = 70; girls = 95).

© 2025 Peterson BS et al. *JAMA Neurology*.

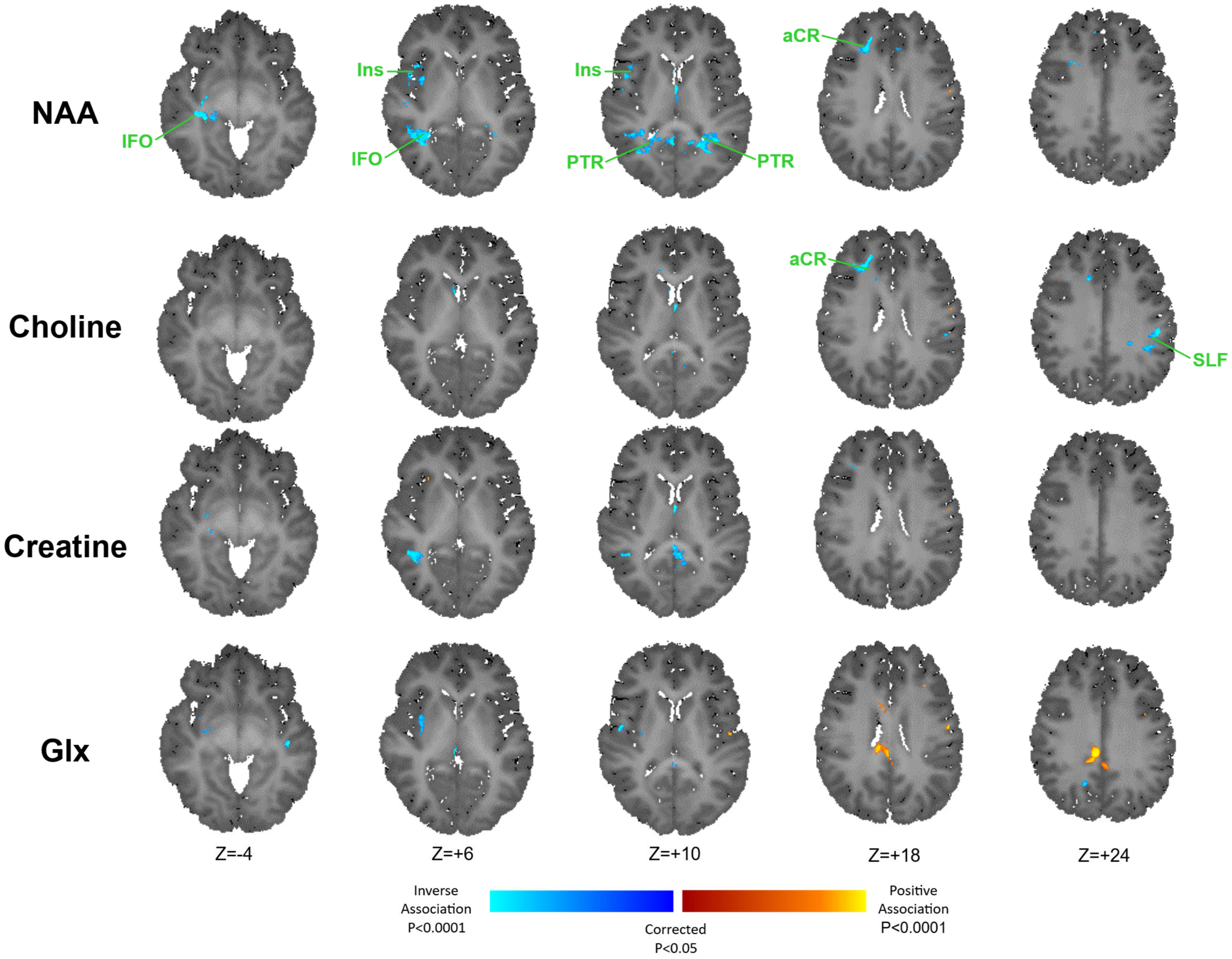

eFigure 15: CPF Exposure Associations with MRS Metabolites While Covarying for Prenatal PAH and PM25 Exposures The regression model was *Imaging Measure* = $\beta_0 + \beta_1$*CPF + β_2*Age + β_3*Sex + β_4*Ethnicity + β_5*Maternal Education + β_6*Material Hardship + β_7*Home Stress +β_8*PAH + β_9*PM_{25} +ε. The dependent Imaging Measure was NAA, Choline, Creatine, or Glx concentration. This model controls for participant age at MRI scan, sex, ethnicity, maternal education, material hardship during pregnancy, home stress at child age 3 years, prenatal PAH exposure, and prenatal PM25 exposure. P-values for β_1 that survived the procedure for False Discovery Rate at an FDR = 0.05 were color-coded as shown in the color bars and then displayed on the template brain. The 2 outliers were excluded, and not all participants had PAH or $PM_{2.5}$ exposure estimates.The number of participants was 200 (Mean Age: 10.76; boys = 93; girls = 107).

© 2025 Peterson BS et al. *JAMA Neurology*.

Sensitivity Analysis
Covarying for Maternal Age

© 2025 Peterson BS et al. *JAMA Neurology*.

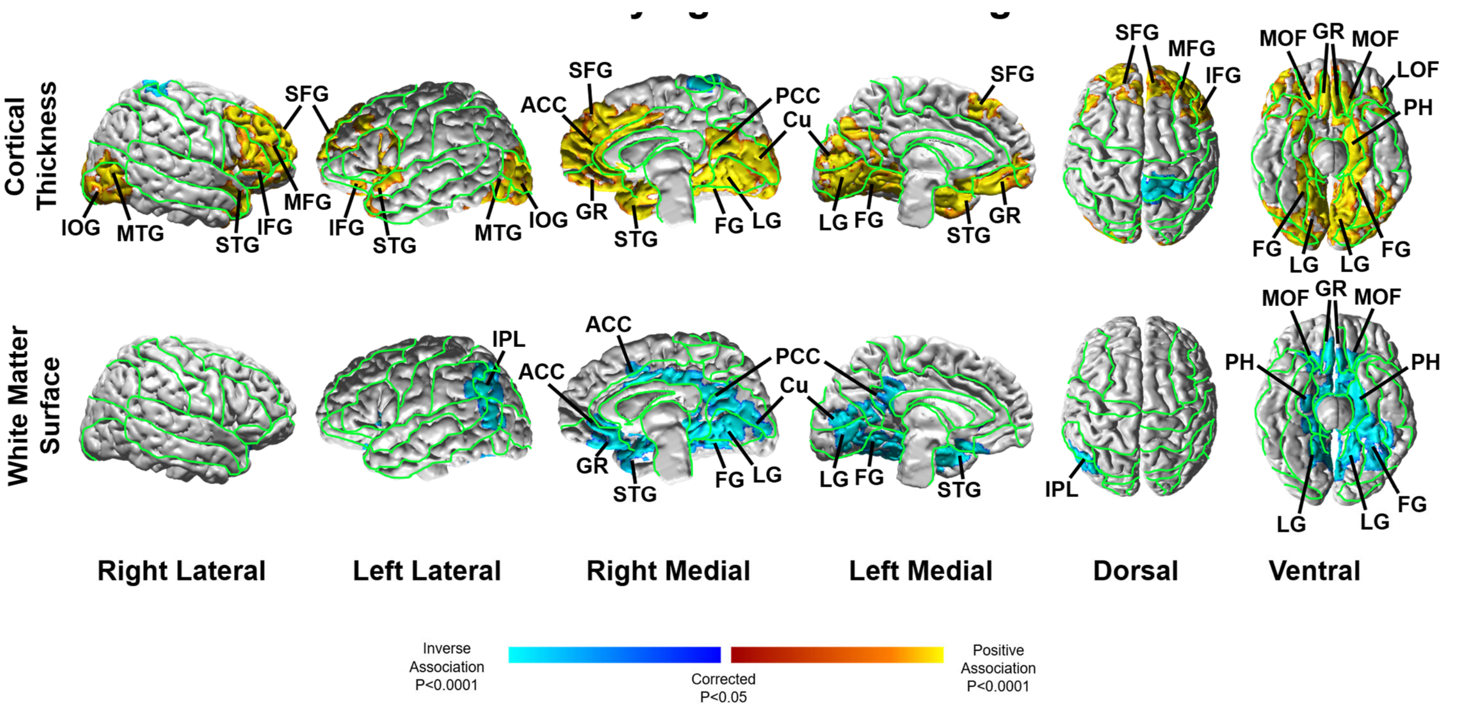

eFigure 16: Statistical Maps of Prenatal CPF Exposure Associations with Anatomical MRI Measures When Covarying for Maternal Age at Birth A regression model tested exposure effects at each point on the cortical or white matter surface: *Imaging Measure* = $\beta_0 + \beta_1$*CPF + β_2*Age + β_3*Sex + β_4*Ethnicity + β_5*Maternal Education + β_6*Material Hardship + β_7*Home Stress + β_8*Maternal Age + ε, with "imaging measure" being either cortical thickness or signed Euclidean distance ("local volume") of the white matter surface from the surface of a template brain. The number of participants for this analysis was 262 (Mean Age: 10.75; boys = 120; girls = 142). The statistical significance (cluster size FWER-corrected p-values) of the associations of exposure with measures of brain structure at each point on the surface of the brain is color-coded, with warm colors (yellow, orange, and red) representing significant positive associations and cooler colors (blue and purple) representing significant inverse associations. Only p-values that survived cluster size FWER correction are plotted, with color-coding of the p-value as shown in the color bar.

© 2025 Peterson BS et al. *JAMA Neurology*.

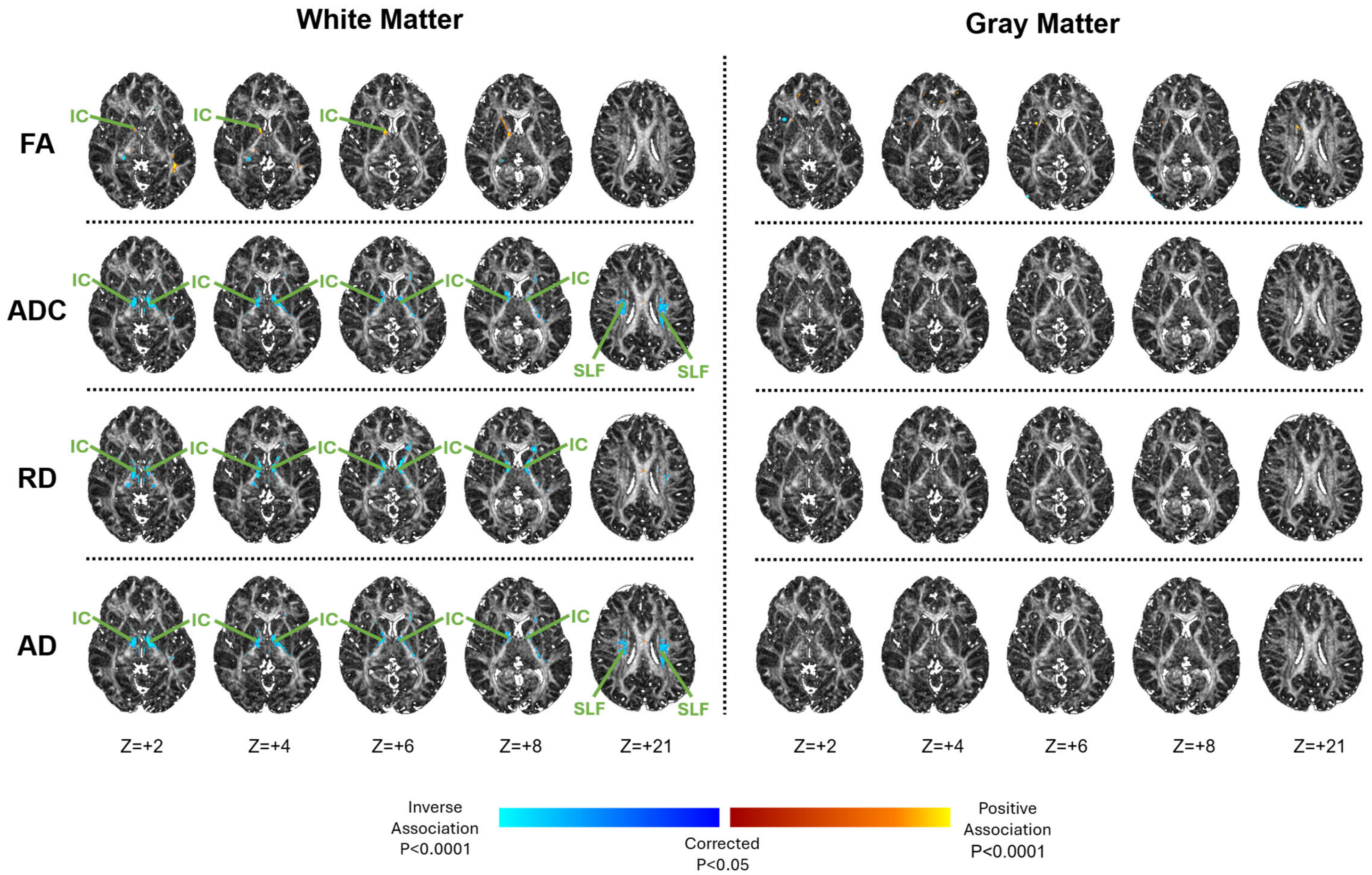

eFigure 17: Statistical Map of CPF Prenatal Exposure Effects on DTI Measures When Covarying for Maternal Age at Birth The regression model that tested CPF exposure effects at each gray or white matter voxel was *Imaging Measure* = $\beta_0 + \beta_1$*CPF* + β_2*Age* + β_3*Sex* + β_4*Ethnicity* + β_5*Maternal Education* + β_6*Material Hardship* + β_7*Home Stress* + β_8*Maternal Age* + ε, with "imaging measure" either fractional anisotropy (FA), average diffusion coefficient (ADC), radial diffusivity (RD), or average diffusion coefficient (ADC). FWER-correction and color coding is identical to Figure 1. The number of participants in this analysis was 202 (Mean Age: 10.78; boys = 89; girls = 113). The Z-values below each column represent the Z-coordinate in Talairach space.

© 2025 Peterson BS et al. *JAMA Neurology*.

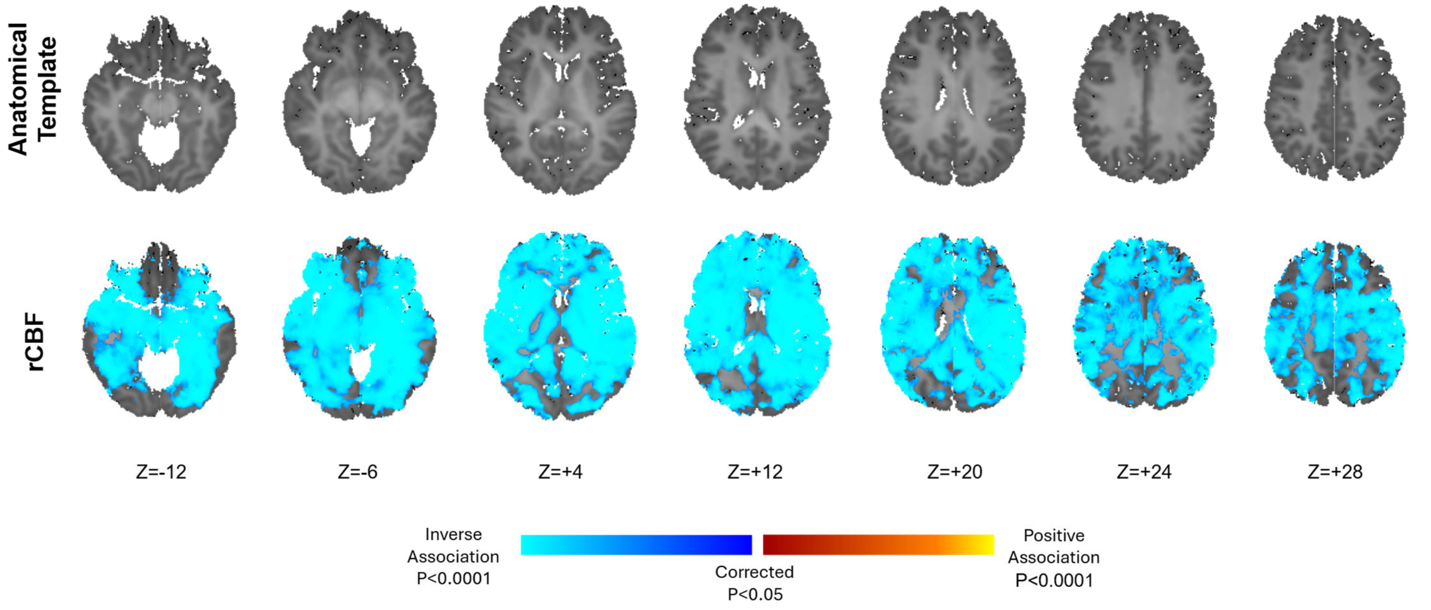

eFigure 18: Statistical Maps of Prenatal CPF Exposure Associations with rCBF When Covarying for Maternal Age at Birth The regression model that tested CPF exposure effects at each white matter voxel in each imaging modality was *Imaging Measure* = $\beta_0 + \beta_1$**CPF* + β_2**Age* + β_3**Sex* + β_4**Ethnicity* + β_5**Maternal Education* + β_6**Material Hardship* + β_7**Home Stress* + β_8**Maternal Age* + ε, with "imaging measure" either rCBF (panel A) or NAA concentration (panel B). FWER-correction and color coding is identical to Figure 1. The number of participants for ASL was 175 (Mean Age: 10.89; boys = 75; girls = 100) and for MRSI was 211 (Mean Age: 10.78; boys = 98; girls = 113). The Z-values below each column represent the Z-coordinate in Talairach space.

© 2025 Peterson BS et al. *JAMA Neurology*.

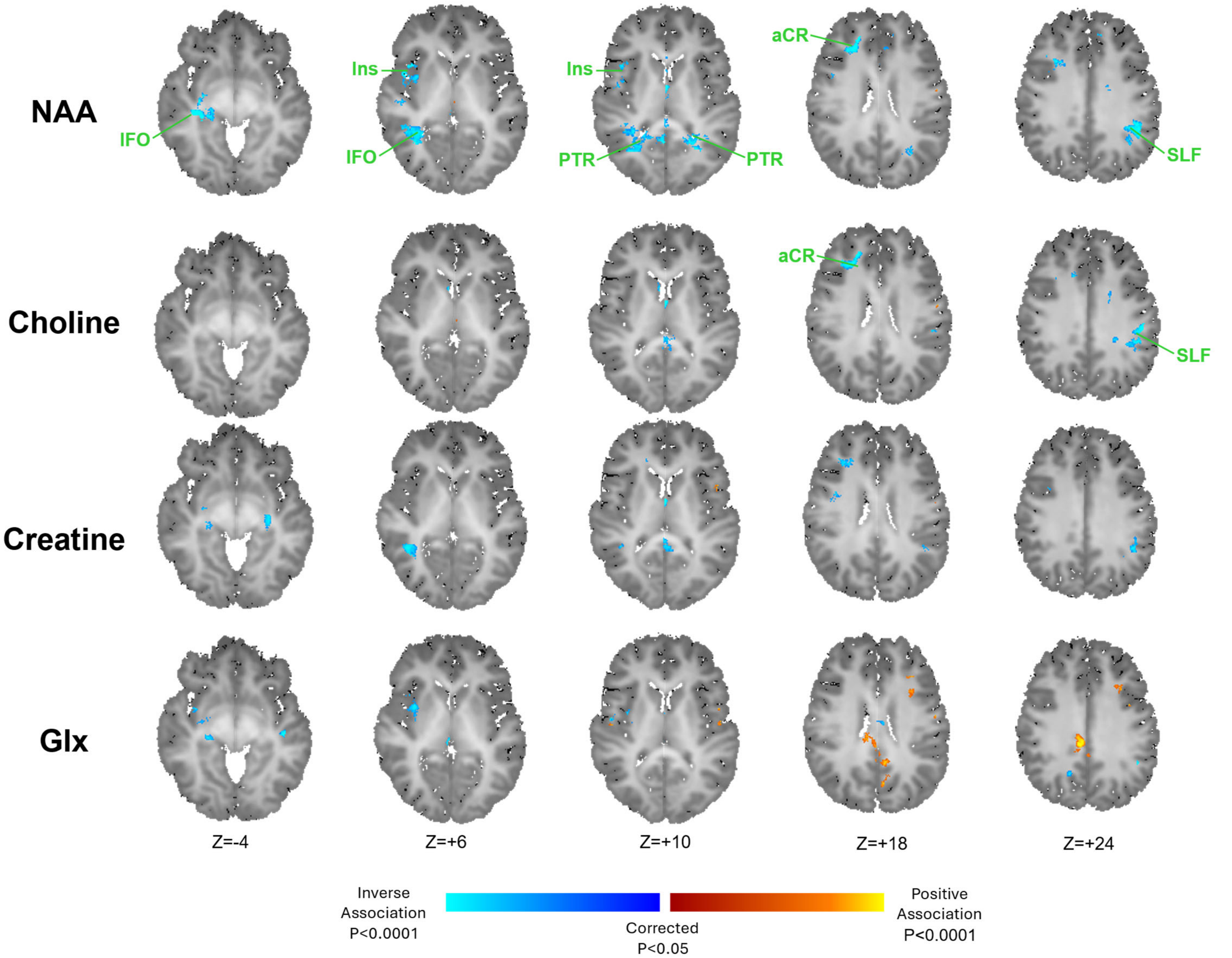

eFigure 19: Association of Prenatal CPF Levels with All Metabolite Concentrations When Covarying for Maternal Age at Birth Shown here are the associations of CPF levels with concentrations for each metabolite measured – NAA, Choline, Creatine, and Glx —when covarying for maternal age at birth. The regression model was *MRS Measure* = $\beta_0 + \beta_1$**CPF* + β_2**Age* + β_3**Sex* + β_4**Ethnicity* + β_5**Maternal Education* + β_6**Material Hardship* + β_7**Home Stress* + β_8**Maternal Age* + ε, where "MRS Measure" was either NAA, Choline, Creatine or Glx concentration. This model controls for participant age at MRI scan, sex, ethnicity, maternal education, material hardship during pregnancy, and home stress at child age 3 years. P-values for β_1 that survived the procedure for False Discovery Rate at an FDR = 0.05 were color-coded as shown in the color bars and then displayed on the template brain. Transaxial slices are positioned parallel to the anterior commissure–posterior commissure line, with Z-levels shown from the Talairach coordinate system. The number of participants was 211 (Mean Age: 10.78; boys = 98; girls = 113).
Abbreviations: aCR: anterior corona radiata; **IFO:** inferior fronto-occipital fasciculus; **Ins:** insula; **PTR:** posterior thalamic radiation; **SLF:** superior longitudinal fasciculus

© 2025 Peterson BS et al. *JAMA Neurology*.

Sensitivity Analysis
Replacing LOD with LOD/2 Values

© 2025 Peterson BS et al. *JAMA Neurology*.

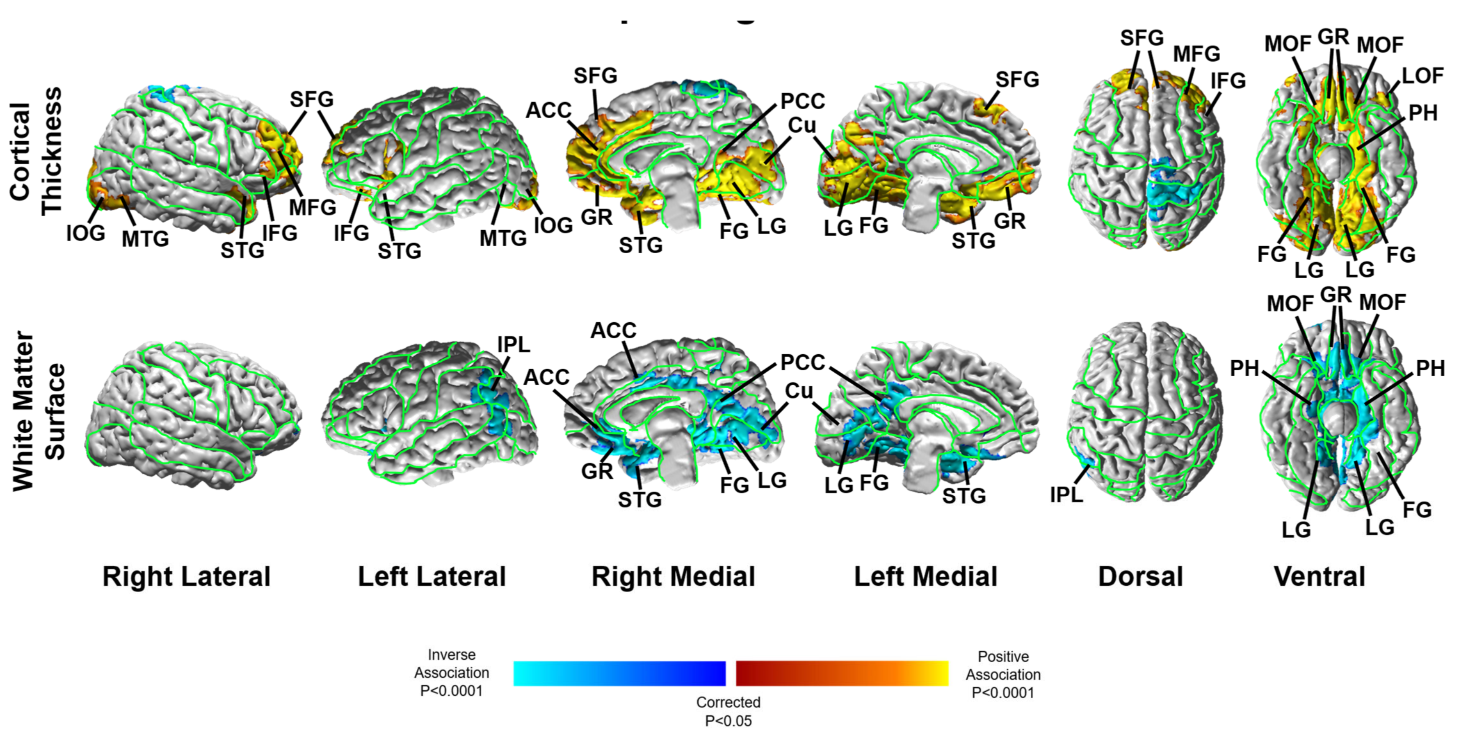

eFigure 20: Statistical Maps of Prenatal CPF Exposure Associations with Anatomical MRI Measures When Replacing LOD Values with LOD/2 CPF values for participants who were at or below the limit of detection (LOD) were replaced with LOD/2.[155-157] A regression model tested exposure effects at each point on the cortical or white matter surface: *Imaging Measure* = $\beta_0 + \beta_1$*CPF* + β_2*Age* + β_3*Sex* + β_4*Ethnicity* + β_5*Maternal Education* + β_6*Material Hardship* + β_7*Home Stress* + ε, with "imaging measure" being either cortical thickness or signed Euclidean distance ("local volume") of the white matter surface from the surface of a template brain. The number of participants for this analysis was 262 (Mean Age: 10.75; boys = 120; girls = 142). The statistical significance (cluster size FWER-corrected p-values) of the associations of exposure with measures of brain structure at each point on the surface of the brain is color-coded, with warm colors (yellow, orange, and red) representing significant positive associations and cooler colors (blue and purple) representing significant inverse associations. Only p-values that survived cluster size FWER correction are plotted, with color-coding of the p-value as shown in the color bar.

© 2025 Peterson BS et al. *JAMA Neurology*.

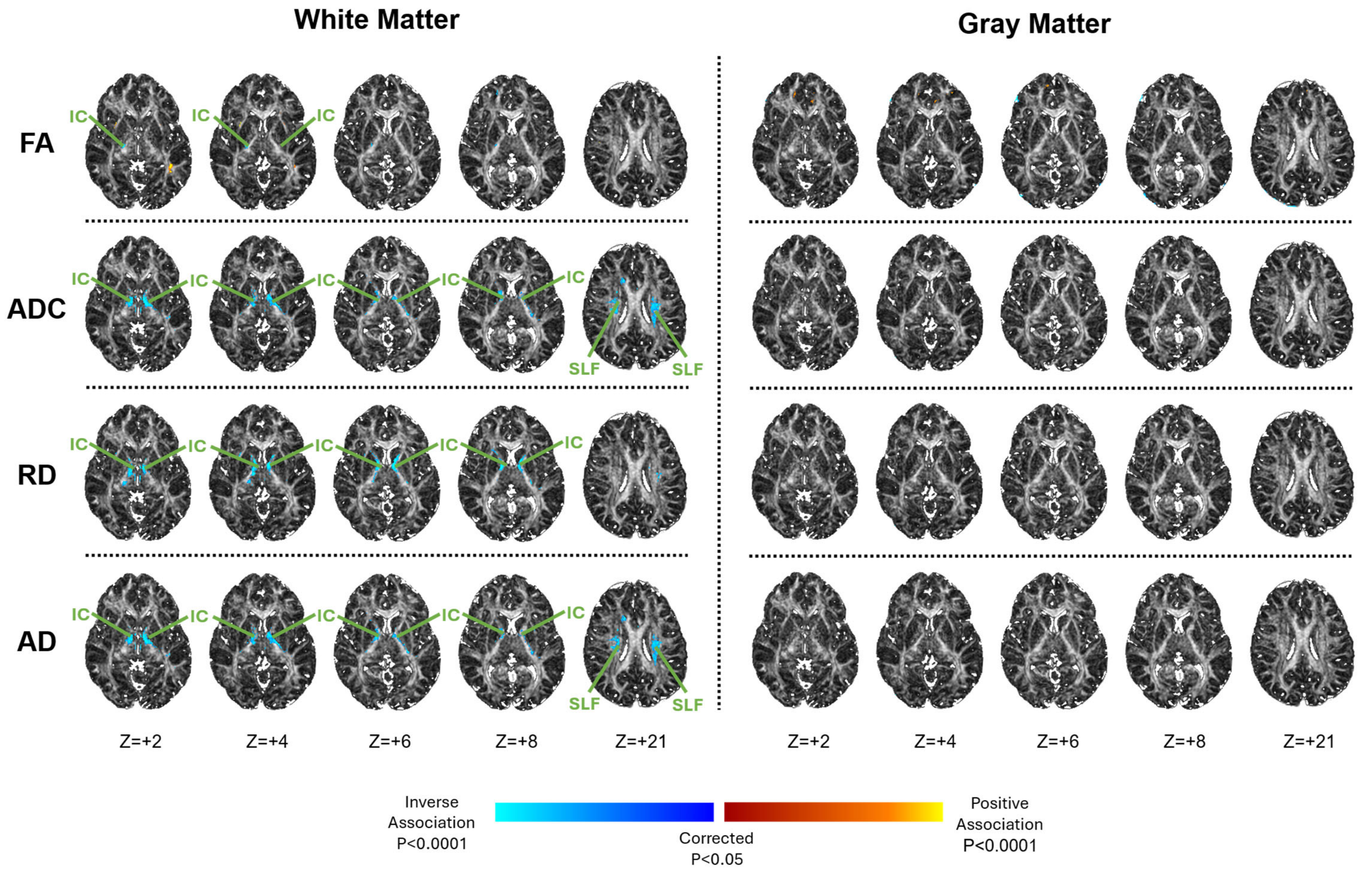

eFigure 21: Statistical Map of CPF Prenatal Exposure Effects on DTI Measures When Replacing LOD Values with LOD/2 CPF values for participants who were at or below the limit of detection (LOD) were replaced with LOD/2.[155-157] The regression model that tested CPF exposure effects at each gray or white matter voxel was *Imaging Measure* = $\beta_0 + \beta_1$*CPF* + β_2*Age* + β_3*Sex* + β_4*Ethnicity* + β_5*Maternal Education* + β_6*Material Hardship* + β_7*Home Stress* + ε, with "imaging measure" either fractional anisotropy (FA), average diffusion coefficient (ADC), radial diffusivity (RD), or average diffusion coefficient (ADC). FWER-correction and color coding is identical to Figure 1. The number of participants in this analysis was 202 (Mean Age: 10.78; boys = 89; girls = 113). The Z-values below each column represent the Z-coordinate in Talairach space.

© 2025 Peterson BS et al. *JAMA Neurology*.

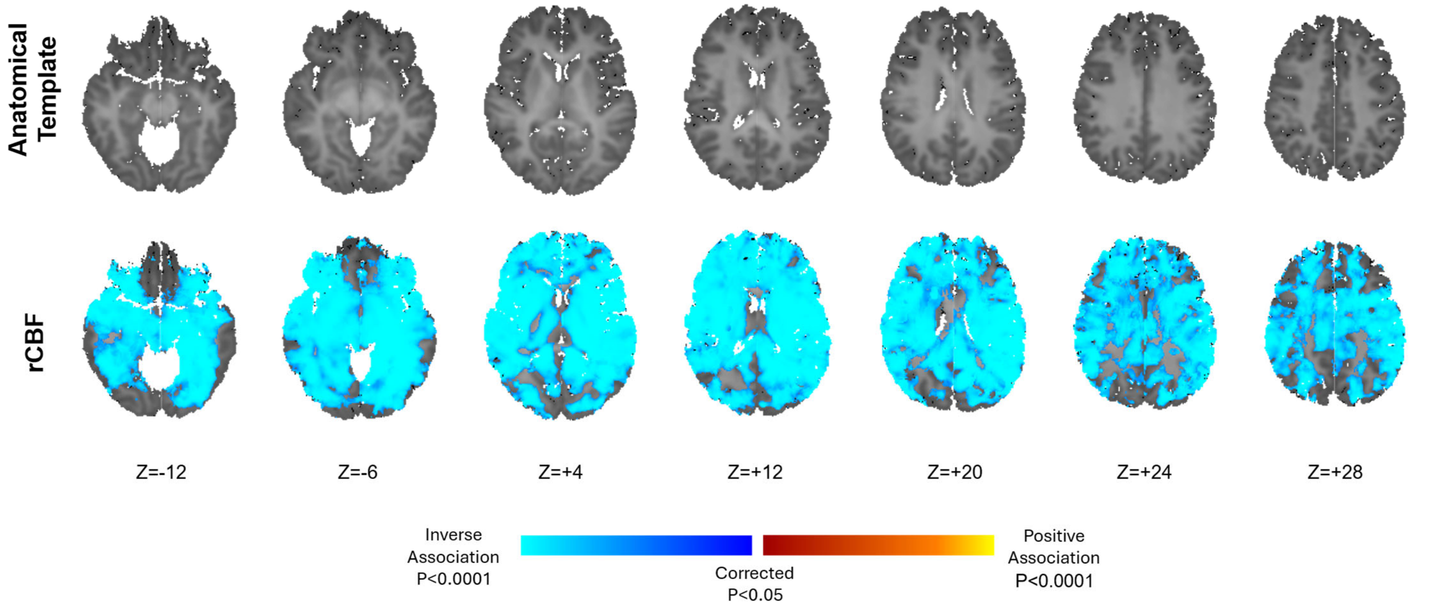

eFigure 22: Statistical Maps of Prenatal CPF Exposure Associations with ASL When Replacing LOD Values with LOD/2 CPF values for participants who were at or below the limit of detection (LOD) were replaced with LOD/2.[155-157] The regression model that tested CPF exposure effects at each white matter voxel in each imaging modality was $Imaging\ Measure = \beta_0 + \beta_1{*}CPF + \beta_2{*}Age + \beta_3{*}Sex + \beta_4{*}Ethnicity + \beta_5{*}Maternal\ Education + \beta_6{*}Material\ Hardship + \beta_7{*}Home\ Stress + \varepsilon$, with "imaging measure" either rCBF (panel A) or NAA concentration (panel B). FWER-correction and color coding is identical to Figure 1. The number of participants for ASL was 175 (Mean Age: 10.89; boys = 75; girls = 100) and for MRSI was 211 (Mean Age: 10.78; boys = 98; girls = 113). The Z-values below each column represent the Z-coordinate in Talairach space.

© 2025 Peterson BS et al. *JAMA Neurology*.

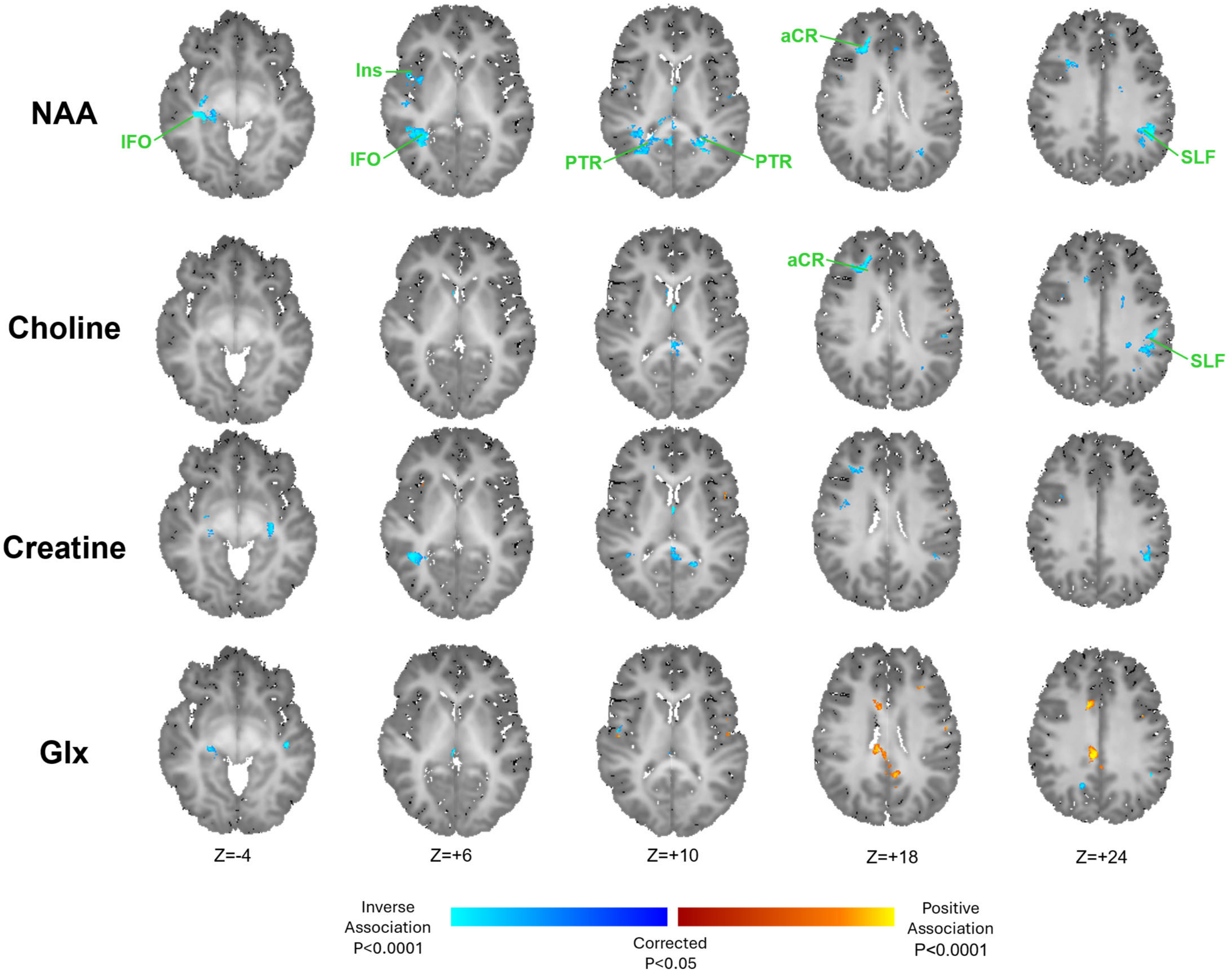

eFigure 23: Association of Prenatal CPF Levels with All Metabolite Concentrations When Replacing LOD Values with LOD/2 Shown here are the associations of CPF levels with concentrations for each metabolite measured – NAA, Choline, Creatine, and Glx -- when replacing CPF values for participants who were at or below the limit of detection (LOD) with LOD/2.[155-157] The regression model was *MRS Measure* = β_0 + β_1*CPF_+ β_2*Age + β_3*Sex + β_4*Ethnicity + β_5*Maternal Education + β_6*Material Hardship + β_7*Home Stress + ε, where "MRS Measure" was either NAA, Choline, Creatine or Glx concentration. This model controls for participant age at MRI scan, sex, ethnicity, maternal education, material hardship during pregnancy, and home stress at child age 3 years. P-values for β_1 that survived the procedure for False Discovery Rate at an FDR = 0.05 were color-coded as shown in the color bars and then displayed on the template brain. Transaxial slices are positioned parallel to the anterior commissure–posterior commissure line, with Z-levels shown from the Talairach coordinate system. The number of participants was 211 (Mean Age: 10.78; boys = 98; girls = 113).

Abbreviations: aCR: anterior corona radiata; **IFO:** inferior fronto-occipital fasciculus; **Ins:** insula; **PTR:** posterior thalamic radiation; **SLF:** superior longitudinal fasciculus.

© 2025 Peterson BS et al. *JAMA Neurology*.

Sensitivity Analysis
CPF Variable Transformations

© 2025 Peterson BS et al. *JAMA Neurology*.

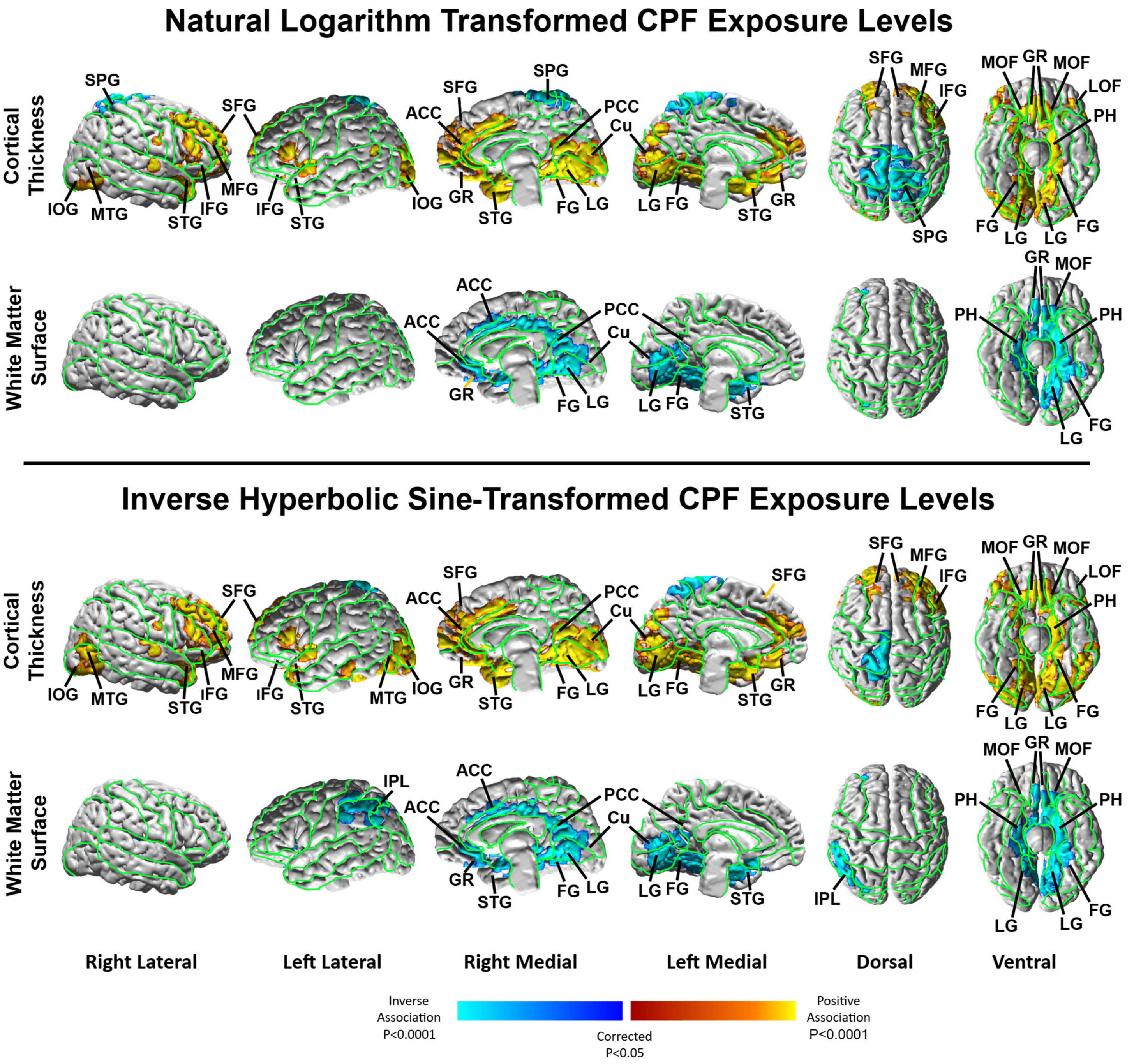

eFigure 24: Transformed CPF Exposure Levels: Associations with Cortical Thickness and White Matter Measures CPF exposure measures included the 2 outlier values that were excluded from the primary analyses, then they were transformed using either the natural logarithm or the inverse hyperbolic sine (IHS) to minimize undue influence of outliers. A regression model tested exposure effects at each point on the surface of the template brain: *Imaging Measure* = $\beta_0 + \beta_1$*transformed(CPF) + β_2*Age + β_3*Sex + β_4*Ethnicity + β_5*Maternal Education + β_6*Material Hardship + β_7*Home Stress + ε*, with the transformed CPF being either ln(CPF) or IHS(CPF) and "imaging measure" being either cortical thickness or white matter distances ("local volumes"). The number of participants for this analysis was 264 (Mean Age 10.75; boys = 121; girls = 143).

Abbreviations: ACC: anterior cingulate cortex; **Cu:** cuneus; **FG:** fusiform gyrus; **GR:** gyrus rectus; **IFG:** inferior frontal gyrus; **IOG:** inferior occipital gyrus; **IPL:** inferior parietal lobule; **ITG:** inferior temporal gyrus; **LOF:** lateral orbitofrontal gyrus; **LG:** lingual gyrus; **MFG:** middle frontal gyrus; **MOF:** middle orbitofrontal gyrus; **MTG:** middle temporal gyrus; **PCC:** posterior cingulate cortex; **SFG:** superior frontal gyrus; **SPG:** superior parietal gyrus; **STG:** superior temporal gyrus.

© 2025 Peterson BS et al. *JAMA Neurology*.

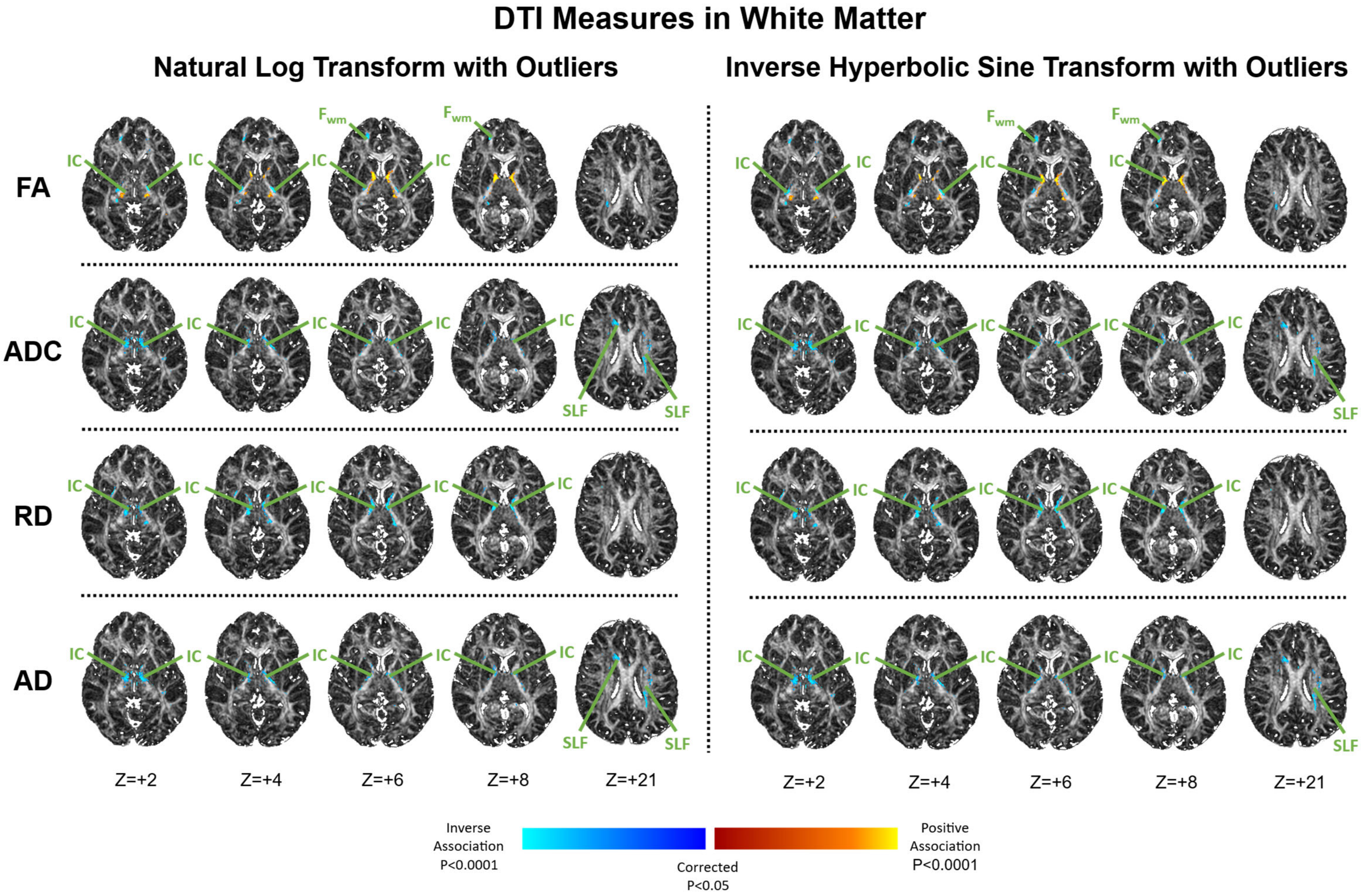

eFigure 25: Transformed CPF Exposure Levels: Associations with DTI White Matter Measures
CPF exposure measures included the 2 outlier values that were excluded from the primary analyses, then they were transformed using either the natural logarithm or the inverse hyperbolic sine (IHS) to minimize undue influence of outliers. A regression model tested exposure effects at each voxel of white matter: *Imaging Measure* = $\beta_0 + \beta_1$**transformed(CPF)* + β_2**Age* + β_3**Sex* + β_4**Ethnicity* + β_5**Maternal Education* + β_6**Material Hardship* + β_7**Home Stress* + ε, with the transformed CPF being either ln(CPF) or IHS(CPF) and "imaging measure" being FA, ADC, RD, or AD. The number of participants for this analysis was 204 (Mean Age: 10.78; boys = 90; girls = 114)

© 2025 Peterson BS et al. *JAMA Neurology*.

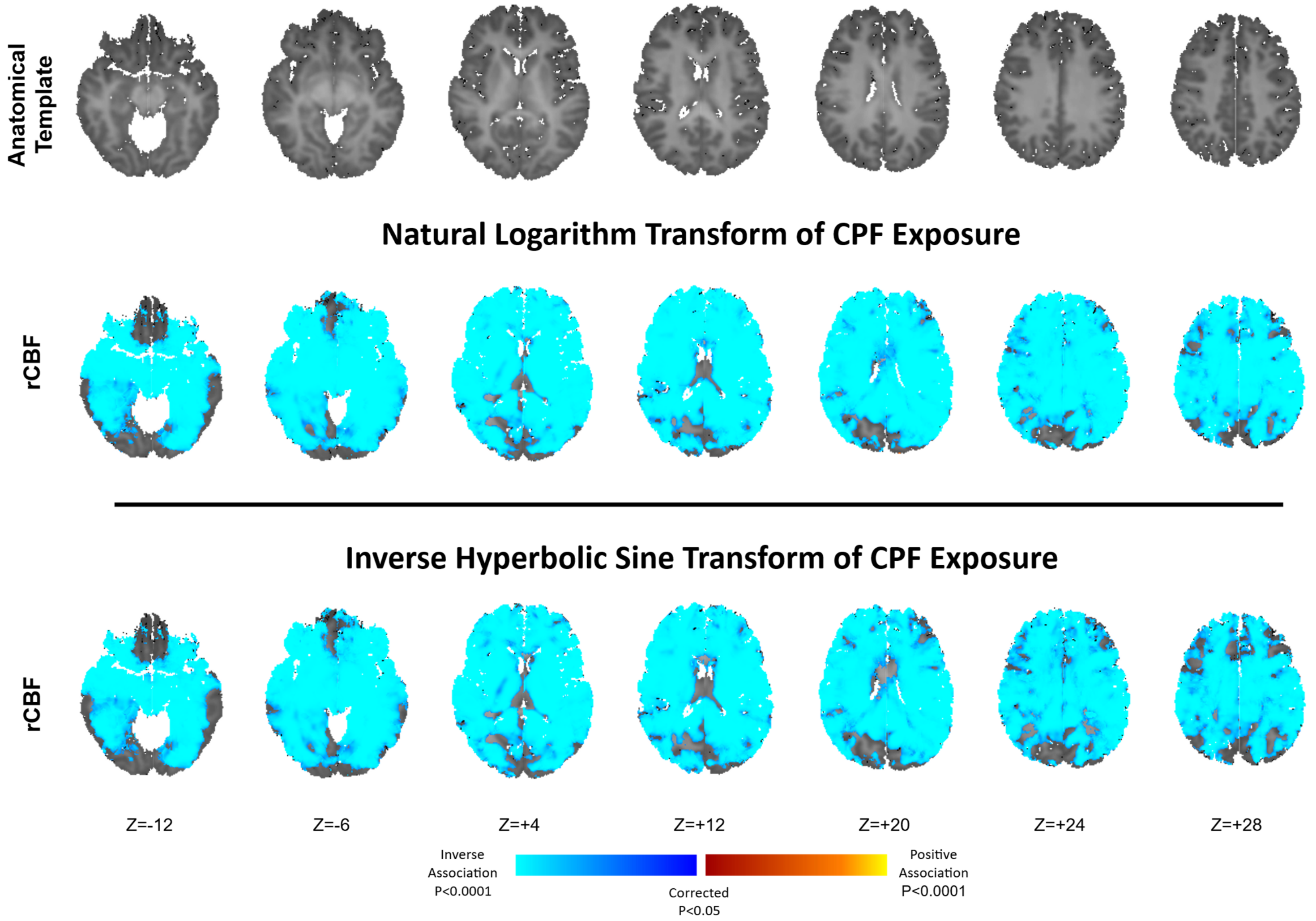

eFigure 26: Transformed CPF Exposure Levels: Associations with rCBF CPF exposure measures included the 2 outlier values that were excluded from the primary analyses, then they were transformed using either the natural logarithm or the inverse hyperbolic sine (IHS) to minimize undue influence of outliers. A regression model tested exposure effects at each voxel of the brain: *Imaging Measure* = $\beta_0 + \beta_1$**transformed(CPF)* + β_2**Age* + β_3**Sex* + β_4**Ethnicity* + β_5**Maternal Education* + β_6**Material Hardship* + β_7**Home Stress* + ε, with the transformed CPF being either ln(CPF) or IHS(CPF) and "imaging measure" being rCBF. The number of participants for this analysis was 176 (Mean Age: 10.89; boys = 76; girls = 100).

© 2025 Peterson BS et al. *JAMA Neurology*.

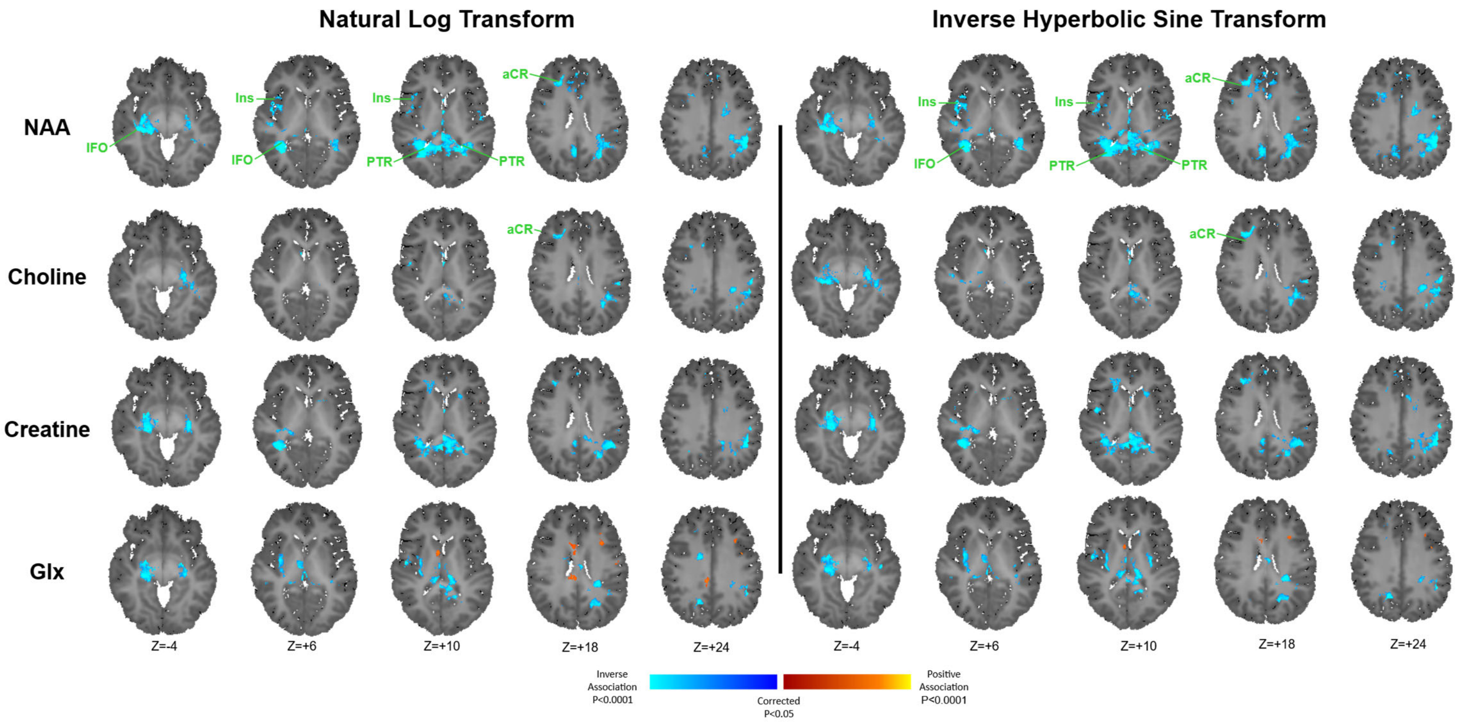

eFigure 27: Transformed CPF Exposure Levels: Associations with Metabolite Concentrations

CPF exposure measures included the 2 outlier values that were excluded from the primary analyses, then they were transformed using either the natural logarithm or the inverse hyperbolic sine (IHS) to minimize undue influence of outliers. A regression model tested exposure effects at each voxel of the brain: *Imaging Measure* = $\beta_0 + \beta_1$**transformed(CPF)* + β_2**Age* + β_3**Sex* + β_4**Ethnicity* + β_5**Maternal Education* + β_6**Material Hardship* + β_7**Home Stress* + ε, with the transformed CPF being either ln(CPF) or IHS(CPF) and "imaging measure" being NAA, Choline, Creatine, or Glx concentration. The number of participants for this analysis was 213 (Mean Age: 10.78; boys = 99; girls = 114).

© 2025 Peterson BS et al. *JAMA Neurology*.
