# Supplementary material for: Brain Abnormalities in Children Exposed Prenatally to the Pesticide Chlorpyrifos
Source: JAMA Neurol. 2025 Aug 18;82(10):1057–68. doi: 10.1001/jamaneurol.2025.2818 (PMC12362277; doi:10.1001/jamaneurol.2025.2818)
Supplement: Supplement 2. — Data sharing statement [file jamaneurol-e252818-s002.pdf]

Data Sharing Statement

Peterson. Brain Abnormalities in Children Exposed Prenatally to the Pesticide Chlorpyrifos. *JAMA Neurol*. Published August 18, 2025. doi:10.1001/jamaneurol.2025.2818

Data
Data available: Yes
Data types: Deidentified participant data, Data dictionary
How to access data: The data that support the findings of this study are available on request from the corresponding author (bpeterson@chla.usc.edu). The data are not publicly available due to privacy or ethical restrictions.
When available: With publication

Supporting Documents
Document types: None

Additional Information
Who can access the data: researchers whose proposed use of the data has been approved
Types of analyses: for a specified purpose
Mechanisms of data availability: with a signed data access agreement
